# MD001, a Novel Peroxisome Proliferator-activated Receptor α/γ Agonist, Improves Glucose and Lipid Metabolism

**DOI:** 10.1038/s41598-018-38281-0

**Published:** 2019-02-07

**Authors:** Seok-Ho Kim, Shin Hee Hong, Young-Joon Park, Jong-Hyuk Sung, Wonhee Suh, Kyeong Won Lee, Kiwon Jung, Changjin Lim, Jin-Hee Kim, Hyoungsu Kim, Kyong Soo Park, Sang Gyu Park

**Affiliations:** 1 Department of Pharmacy, College of Pharmacy and Institute of Pharmaceutical Sciences, CHA University, Pocheon, Gyeonggi-do 11160 Korea; 2 College of Pharmacy, Ajou University, Suwon, Gyeonggi-do 16499 Korea; 3 College of Pharmacy, Yonsei University, Songdo, Incheon 405-750 Korea; 4 College of Pharmacy, Chung-Ang University, Seoul, 156-756 Korea; 5 Marine Biotechnology Research Center, Korea Institute of Ocean Science & Technology 787 Haeanlo, Ansan Gyeonggi-do, 426-744 Korea; 6 The Division of Endocrinology and Metabolism, Department of Internal Medicine, Seoul National University College of Medicine, Seoul, 03080 Korea

## Abstract

Peroxisome proliferator-activated receptor (PPAR)-α/γ dual agonists have been developed to treat metabolic diseases; however, most of them exhibit side effects such as body weight gain and oedema. Therefore, we developed a novel PPARα/γ dual agonist that modulates glucose and lipid metabolism without adverse effects. We synthesised novel compounds composed of coumarine and chalcone, determined their crystal structures, and then examined their binding affinity toward PPARα/γ. We investigated the expression of PPARα and PPARγ target genes by chemicals in HepG2, differentiated 3T3-L1, and C2C12 cells. We examined the effect of chemicals on glucose and lipid metabolism in *db/db* mice. Only MD001 functions as a PPARα/γ dual agonist *in vitro*. MD001 increased the transcriptional activity of PPARα and PPARγ, resulting in enhanced expression of genes related to β-oxidation and fatty acid and glucose uptake. MD001 significantly improved blood metabolic parameters, including triglycerides, free fatty acids, and glucose, in *db/db* mice. In addition, MD001 ameliorated hepatic steatosis by stimulating β-oxidation *in vitro* and *in vivo*. Our results demonstrated the beneficial effects of the novel compound MD001 on glucose and lipid metabolism as a PPARα/γ dual agonist. Consequently, MD001 may show potential as a novel drug candidate for the treatment of metabolic disorders.

## Introduction

Energy metabolism is maintained by three major organs - adipose tissue, liver, and muscle - and crosstalk among them is essential for homeostasis. Surplus energy due to excess nutrient ingestion, reduced energy consumption in the cell, or both induces over-production of triglycerides (TG), which are stored in adipose tissue, skeletal muscle, and the liver^1,2^. The increase in free fatty acids (FFA) gives rise to insulin resistance by desensitizing insulin-mediated signal transduction, resulting in aberrant lipid metabolism associated with metabolic diseases including type 2 diabetes, obesity, hyperglycaemia, hyperlipidaemia, hepatic steatosis, atherosclerosis, and cardiovascular diseases^2^. Over the last few decades, a variety of small molecules have been developed to improve abnormal lipid metabolism.

Peroxisome proliferator-activated receptors (PPARs), members of the nuclear receptor family comprised of PPARα, PPARβ/δ, and PPARγ, are known to be critical regulators of lipid metabolism^3^. The activation of PPARα or PPARβ/δ stimulates fat consumption by inducing the expression of genes associated with beta-oxidation, resulting in the improvement of hyperlipidaemia^4,5^. PPARγ, on the other hand, promotes the mobilisation of fatty acid into adipocytes by encouraging adipogenesis and upregulating the expression of genes related to fatty acid transport, including those for CD36 and adipocyte fatty acid-binding protein (FABP), to reduce lipotoxicity^3,6^.

Peroxisome proliferator-activated receptor agonists have been widely used to treat metabolic diseases such as hyperglycaemia and lipid dysregulation^7,8^. Fibrate, a selective PPARα agonist, relieves hyperlipidaemia by promoting β-oxidation and induces a minor reduction in body weight^5,9^. However, fibrates are known to cause severe side effects, including gallstone formation and hepatotoxicity^10,11^. Thiazolidinedione (TZD), a synthetic PPARγ agonist, has been approved to reduce blood glucose levels and lipotoxicity by improving insulin resistance. TZD upregulates PPARγ target genes involved in lipid and glucose metabolism, insulin signal transduction, and adipocyte differentiation. For instance, PPARγ activation by TZD increases the expression of CD36 and FABP for lipid storage and the expression of glucose transporter for glucose uptake in adipocytes, liver, and skeletal muscle, thereby reducing the amount of fatty acid and glucose levels in blood by increasing insulin sensitivity. However, since TZD induces storage of fatty acids and glucose rather than consumption in the cell, it has been associated with several concerns, including body weight gain and myocardial infarction^12–14^. Therefore, the dual activation of PPARα and PPARγ is proposed to be more beneficial with limited adverse effects^15–17^. A number of PPARα/γ dual agonists have been developed and tested^18,19^. However, a majority of these drugs showed severe adverse effects, including heart failure, renal failure, urinary cancer, body weight gain, stroke, and anemia^18–22^, necessitating the development of novel PPARα/γ dual agonists without side-effects.

In a previous study, we isolated interruptin B (Int B) from *Cyclosorus terminans*, and showed its stimulatory effect on adipogenic differentiation and glucose consumption^23^. In the current study, we tried to synthesise various constitutional isomers of Int B to find a novel agonist targeting PPARs and investigate its ability to improve metabolic disorders *in vitro* and *in vivo*.

## Results

### Chemical synthesis

**Figure 1:**
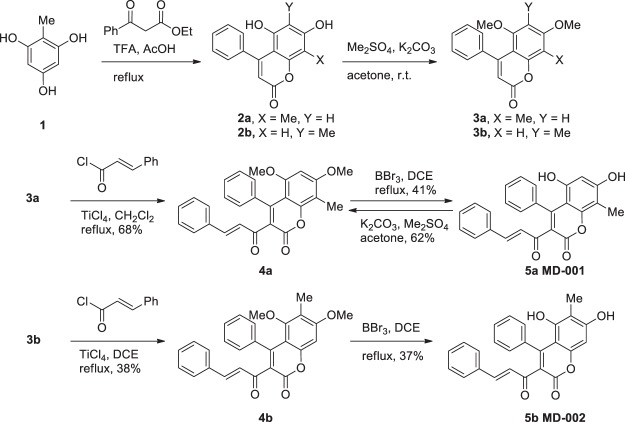
Scheme of Int B isoform synthesis.

Our synthetic procedure is summarised in Fig. 1. The synthesis commenced with a well-known Pechmann condensation of commercially available (**1**)^24^ and ethyl benzoyl acetate to afford a roughly 1:1 mixture of **2a** and **2b** owing to the presence of two isomeric ortho and para hydroxyl groups to the methyl group. Compounds **2a** and **2b** proved to be labile during SiO_2_ column chromatography and insoluble in various solvents probably due to the 1,3-dihydroxy group. Thus, the mixture of **2a** and **2b** was methylated in the presence of K_2_CO_3_ and Me_2_SO_4_^25,26^ to give rise to **3a** and **3b**, pleasingly, which were readily dissociable and stable during the separation process. The structure of **3b** was confirmed by the X-ray crystallography (Supplementary Fig. S1a). Having a secured route to the two isomers **3a** and **3b**, we next turned our attention to the fundamental Friedel-Crafts acylation. Given that acetyl groups are easily condensed with the benzaldehyde to afford the cinnamoyl groups under basic conditions, we initially attempted Friedel-Crafts acetylation. However, despite our efforts to force the reaction with various Lewis acids such as BF_3_∙OEt_2_, SnCl_4_, and TiCl_4_, all failed to yield any of the desired products. We then attempted to directly introduce a cinnamoyl group. Friedel-Crafts cinnamoylation of **3a** in the presence of BF_3_∙OEt_2_ as a Lewis acid did not yield **4a**. However, to our delight, the reaction of **3a** with SnCl_4_ gave rise to the desired product **4a** even though 50% of the starting material **3a** remained. Finally, the treatment of **3a** with TiCl_4_ in refluxing CH_2_Cl_2_ furnished **4a** at a good yield (68%) (Supplementary Table S2). The reaction of **3b** with TiCl_4_ in refluxing CH_2_Cl_2_ did not provide **4b**, but in refluxing dichloroethane gave rise to **4b** at a moderate yield (38%, 70% conversion of **3a**). The structure of **4a** was verified from by X-ray crystallography (Supplementary Fig. S1b). Finally, global deprotection by the action of BBr_3_ in refluxing dichloroethane provided **5a** (41%) and **5b** (37%) respectively^27–29^.

Representative spectral data for **5a** (MD001): yield −41%; yellow solid; m.p. 274–278 °C; ^1^H-NMR (600 MHz, acetone-*d*_6_): *δ* 9.31 (s, 1H), 8.58 (s, 1H), 7.55–7.54 (m, 2H), 7.53 (d, *J* = 16.3 Hz, 1H), 7.34–7.31 (m, 3H), 7.24–7.20 (m, 2H), 7.20–7.14 (m, 3H), 6.72 (s, 1H), 6.59 (d, *J* = 16.3 Hz, 1H), 6.30 (s, 1H), 2.16 (s, 3H); ^1^H NMR (400 MHz, DMSO-*d*_6_): *δ* 10.46 (s, 1H), 9.82 (s, 1H), 7.63 (d, *J* = 6.0 Hz, 2H), 7.49 (d, *J* = 16.3 Hz, 2H), 7.39–7.37 (m, 3H), 7.22–7.16 (m, 5H), 6.63 (d, *J* = 16.3 Hz, 1H), 6.27 (s, 1H), 2.11 (s, 3H); ^13^C-NMR (150 MHz, acetone-*d*_6_):* δ* 193.5, 171.5, 161.2, 159.9, 156.6, 155.4, 153.8, 146.6, 138.6, 136.1, 131.8, 131.1, 129.7, 129.2, 128.9, 128.8, 128.5, 122.9, 104.6, 102.8, 100.4, 8.3; IR (ATR) *ν*_max_ 3373, 2931, 1716, 1604, 1361, 1138, 1099 cm^−1^; HRMS (ESI) m/z calculated for C_25_H_19_O_5_ (M + H)^+^ 399.1232, detected 399.1223.

### MD001 activates PPARα and PPARγ

**Figure 2:**
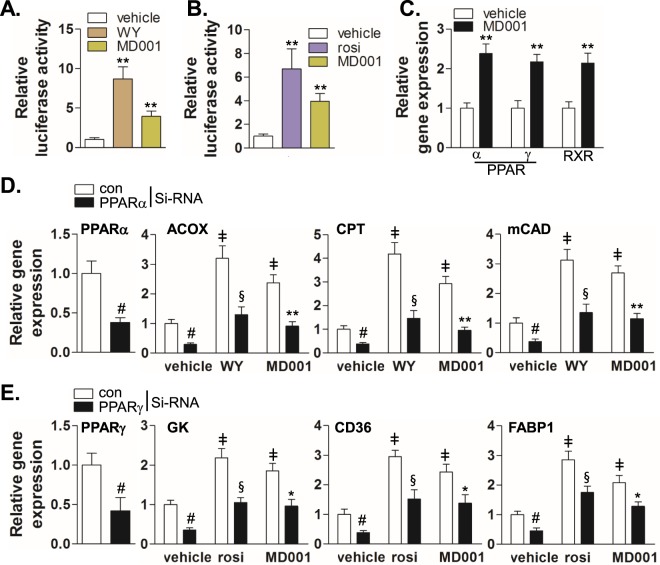
MD001 induces the expression of target genes through PPARα and PPARγ activation. HEK293 cells were transiently co-transfected with human HA-PPARα (**A**) and HA-PPARγ (**B**) expression vectors along with the reporter plasmid (PPRE-pk-Luc) or control reporter plasmid (pk-Luc) with *Renilla* vector for 24 h. Cells were treated with MD001 (10 μM), rosiglitazone (rosi) (10 μM) or WY14643 (WY) (10 μM) for 24 h. Luciferase activity was normalised to *Renilla* luciferase activity as described in the Methods section. **, vs. vehicle. (**C**) HepG2 cells were treated with MD001 (10 μM) for 24 h, and total RNA was isolated and synthesised into cDNA. Relative expression was quantitated using qRT-PCR. **, vs. vehicle. (**D**,**E**) HepG2 cells were transfected with control siRNA, PPARα siRNA (**D**), or PPARγ siRNA (**E**) for 48 h and treated with vehicle, WY14643 (10 μM), rosiglitazone (10 μM) or MD001 (10 μM) for 24 h. Subsequently, cells were harvested for total RNA isolation. Relative expression of target genes was quantitated using qRT-PCR. ^#^, vs. control siRNA; ^‡^, vs. control siRNA/vehicle; ^§^, vs. control siRNA/WY; * and **, vs. control siRNA/MD001. Data represent the mean ± SD of three independent experiments. *P < 0.05, **P < 0.01, ^#^*P* < 0.01, ^‡^*P* < 0.01, and ^§^*P* < 0.01.

First, we assessed whether these compounds could increase the transcriptional activity of PPARα or PPARγ. Out of all of the compounds synthesised, only **5a** (MD001) promoted simultaneous transcriptional activity of PPARα and PPARγ (Supplementary Fig. S2). Therefore, we assessed the binding of synthesised MD001 to PPARα, PPARβ/δ, and PPARγ using surface plasmon resonance (SPR) analysis. As presented in Supplementary Table S3, MD001 was shown to bind to PPARα (K_D_ =  9.55 ± 0.8 μM) and PPARγ (K_D_ = 0.14  ± 0.03 μM), but not PPARβ/δ. In addition, we compared the stimulatory effect of MD001 on PPARα and PPARγ with that of WY14643 and rosiglitazone, agonists of PPARα and PPARγ, respectively. A luciferase activity assay using PPRE in HEK293 cells showed that MD001 significantly increased transcription via PPARα and PPARγ activation (Fig. 2A,B). Next, we examined whether MD001 could activate or increase the expression of PPARα and PPARγ in HepG2 cells and found that MD001 significantly increased the expression of PPARα, PPARγ, and retinoid X receptor (RXR) genes (Fig. 2C). To confirm the MD001-mediated activation of PPARα and PPARγ in hepatocytes, we assessed the expression of their target genes in HepG2 cells. As shown in Fig. 2D,E, MD001 significantly increased the expression of PPARα target genes related to β-oxidation, such as acyl-CoA oxidase (*ACOX*), carnitine-palmitoyl transferase (*CPT*), and middle-chain acyl-CoA dehydrogenase (*mCAD*); the siRNA–mediated knockdown of PPARα showed the opposite effect (Fig. 2D, Supplementary Fig. S3A). In addition, MD001 significantly increased the expression of glycerol kinase (GK), *CD36*, and *FABP1*, all of which are target genes of PPARγ. The opposite effect was observed upon siRNA–mediated knockdown of PPARγ (Fig. 2E, Supplementary Fig. S3B). These results suggest that MD001 may act as a dual agonist and regulate metabolism via specific activation of PPARα and PPARγ.

**Figure 3:**
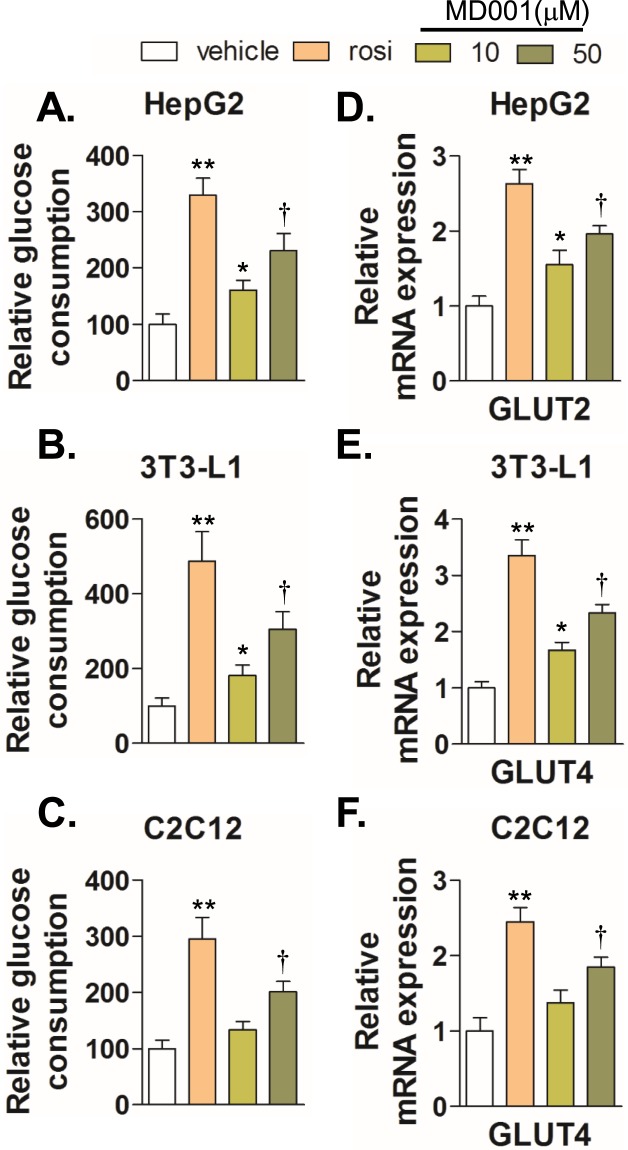
MD001 promotes glucose consumption by induction of GLUT expression. HepG2 (**A**), differentiated 3T3-L1 adipocytes (**B**), and differentiated C2C12 myotubes (**C**) were treated with vehicle, MD001 (10, 50 μM), or rosiglitazone (rosi, 10 μM) for 24 h; glucose consumption was examined as described in the Methods section. Cells were harvested for total RNA isolation. Relative expression levels of GLUT2 (**D**) and GLUT4 (**E**,**F**) were quantitated using qRT-PCR. *, **, and ^†^ vs. vehicle. Data represent the mean ± SD of three independent experiments. *P < 0.05, **P < 0.01, and ^†^*P* < 0.01.

Studies have proven PPARγ to be a traditional molecular target for the development of anti-diabetic drugs that improve insulin sensitivity and glucose tolerance. Therefore, we compared the ability of MD001 to enhance glucose metabolism with that of rosiglitazone in HepG2, differentiated 3T3-L1, and C2C12 myotubes. MD001 significantly increased glucose consumption in a dose-dependent manner (Fig. 3A–C). In addition, quantitative RT-PCR analysis showed that MD001 significantly increased the expression of glucose transporter GLUT2 (HepG2) and GLUT4 (3T3-L1 and C2C12), suggestive of its stimulatory effect on glucose metabolism at least in part through increased expression of glucose transporter (Fig. 3D–F).

**Figure 4:**
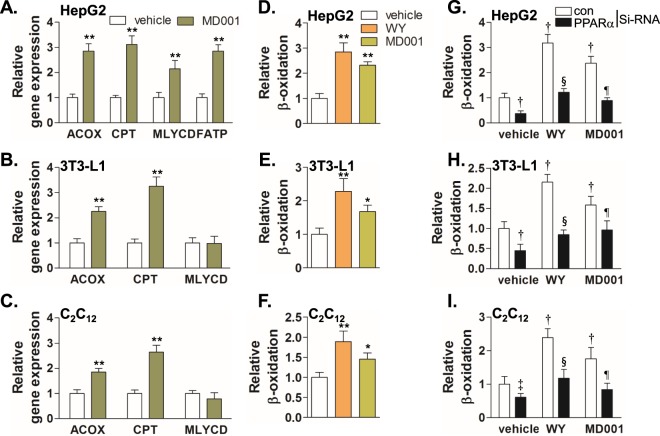
MD001 stimulates fatty acid oxidation *in vitro*. HepG2 (**A**), differentiated 3T3-L1 adipocyte (**B**), and differentiated C2C12 myotubes (**C**) were treated with vehicle or MD001 (10 μM) for 24 h and total RNA was isolated for cDNA synthesis. Relative gene expressions were analysed by qRT-PCR. HepG2 (**D**), differentiated 3T3-L1 adipocytes (**E**), and differentiated C2C12 myotubes (**F**) were treated with vehicle, MD001 (10 μM), or WY14643 (WY) (10 μM) for 24 h and the fatty acid oxidation rate was analysed as described in the Methods section. To confirm whether the fatty acid oxidation rate enhanced by MD001 is mediated by PPARα, HepG2 (**G**), differentiated 3T3-L1 (**H**), and differentiated C2C12 myotubes (**I**) were transfected with control or PPARα siRNA (20 nM) for 48 h, followed by treatment with vehicle, MD001 (10 μM), or WY14643 (WY) (10 μM) for 24 h. WY14643 was treated as positive control. *, **, vs. vehicle; ^†^ and ^‡^, vs. vehicle/control siRNA; ^§^, vs. WY/control siRNA; ^¶^, vs. MD001/control siRNA. Data represent the mean ± SD of three independent experiments. *P < 0.05, **P < 0.01, ^†^*P* < 0.01, ^‡^*P* < 0.05, ^§^*P* < 0.01, and ^¶^*P* < 0.05.

To further examine whether MD001 could increase β-oxidation via PPARα activation, we analysed the expression of PPARα target genes related to β-oxidation in HepG2, differentiated 3T3-L1, and C2C12 myotubes. MD001 significantly increased the expression levels of *ACOX*, *CPT*, malonyl-CoA decarboxylase (*MLYCD*), and fatty acid transporter (*FATP*) in HepG2, and ACOX and CPT in both 3T3-L1 and C2C12 cells (Fig. 4A–C). Therefore, we evaluated whether MD001 could stimulate fatty acid oxidation and found that MD001 significantly increased the β-oxidation rate in HepG2 cells (Fig. 4D); similar results were observed in differentiated 3T3-L1 and C2C12 myotubes (Fig. 4E,F). We examined whether the increased expression of PPARα target genes and enhanced β-oxidation is dependent on PPARα by suppressing PPARα expression using PPARα-specific siRNA. The MD001-mediated increase in β-oxidation was abrogated following downregulation of PPARα expression, thereby confirming the stimulatory effect of MD001 on β-oxidation via PPARα activation (Fig. 4G–I, Supplementary Fig. S4A–C).

### MD001 improves metabolic profiles in *db/db* mice

**Figure 5:**
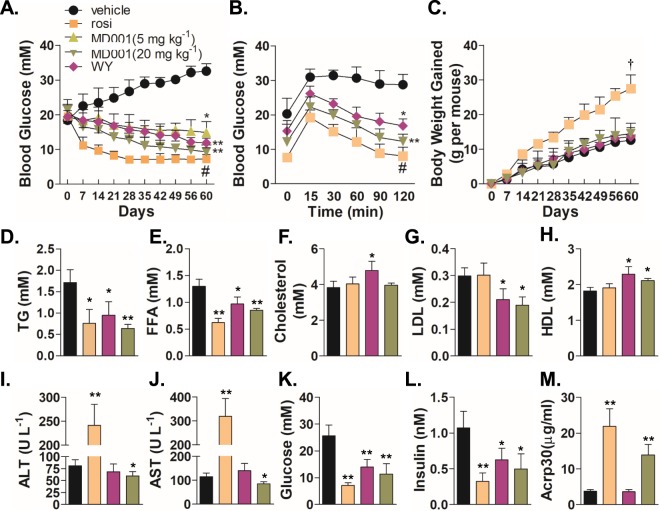
MD001 improves metabolic parameters in *db/db* mice. (**A**) Changes in blood glucose levels was monitored in *db/db* mice administered once a day with vehicle, WY14643 (WY, 20 mg/kg), rosiglitazone (rosi, 20 mg/kg), or MD001 (n = 5–6 per group) as indicated. (**B**) Oral glucose tolerance test (OGTT) was performed with sterile glucose (1 g/kg) at eight weeks after drug administration (n = 5–6 per group). (**C**) Body weight changes during drug administration were monitored for 60 days (n = 5–6 per group). (**D**–**M**) The change in various blood metabolites was examined in *db/db* mice administered once a day with vehicle, WY14643 (WY), rosiglitazone (rosi), or MD001 (n = 5–6 per group). *, **, and ^#^, vs. vehicle; ^†^, vs. vehicle, MD001(5 mg/kg), and MD001(20 mg/kg). The data represent the mean ± S.D. *P < 0.05, **P < 0.01, ^#^*P* < 0.001, and ^†^*P* < 0.01.

To investigate its effects *in vivo*, MD001 was administered once a day to wild type C57BL/6J and diabetic *db/db* mice. MD001 significantly decreased blood glucose levels in a dose-dependent manner in *db/db* mice (Fig. 5A). An oral glucose tolerance test (OGTT) revealed that MD001 significantly decreased blood glucose levels in *db/db* mice (Fig. 5B, Supplementary Fig. S5A). In addition, an intraperitoneal insulin tolerance test (IPITT) showed that MD001 lowered blood glucose levels by increasing insulin sensitivity (Supplementary Fig. S5B,C). In the livers of specimens in the diabetic animal model, the expression of genes related to gluconeogenesis is upregulated, contributing to hyperglycaemia^30^. Given that MD001 was shown to recover insulin sensitivity and improve blood glucose levels in *db/db* mice as shown above, the effect of MD001 on gene expression related to gluconeogenesis was examined. As shown in Supplementary Fig. S5D, MD001 decreased the expression of phosphoenolpyruvate carboxykinase (*PEPCK*) and glucose-6-phosphatase (*G6Pase*) in the livers of *db/db* mice. TZDs, including rosiglitazone, pioglitazone, and troglitazone, are known to induce severe body weight gain as an adverse effect both in animals and humans^9,12,31,32^. Therefore, we evaluated the effect of MD001 on body weight. As shown in Fig. 5C, rosiglitazone significantly induced body weight gain in *db/db* mice compared to the vehicle control; on the contrary, MD001 and WY14643 showed no induction of body weight gain in both wild type C57BL/6J and *db/db* mice without changes in food ingestion (Supplementary Fig. S6). As the agonist-mediated activation of PPARα or PPARγ has been known to lower lipid levels as well as blood glucose in diabetic patients^7,8^, we examined whether MD001 could lower lipid levels. Rosiglitazone and WY14643, used as positive controls, significantly decreased blood TG, FFA, and glucose levels in *db/db* mice (Fig. 5D,E,K), consistent with a previous study^9^. As a PPARα/γ dual agonist, MD001 also significantly decreased blood TG, FFA, insulin, and glucose levels in *db/db* mice (Fig. 5A,D,E,K,L). However, in wild type C57BL/6J mice, MD001 did not have any effect on the levels of plasma lipids and blood glucose when compared to the vehicle group (Supplementary Fig. S7). In addition, although MD001 did not affect the total cholesterol levels (Fig. 5F), it significantly decreased LDL and increased HDL levels (Fig. 5G,H), indicating that MD001 may improve cholesterol metabolism in diabetic animal models. While rosiglitazone significantly increased blood alanine aminotransferase (ALT) and aspartate aminotransferase (AST) levels, MD001 significantly reduced blood ALT and AST levels in *db/db* mice; no effect was observed on blood ALT and AST levels in wild type C57BL/6 mice, suggestive of the absence of toxic effects of MD001 on the liver, unlike pure PPARγ agonists (Fig. 5I,J, Supplementary Fig. S7F,G)^33,34^. In addition, MD001 increased the serum adiponectin (Acrp30) levels (Fig. 5M). Furthermore, rosiglitazone treatment resulted in significant increases in liver weight (40%) and fat mass (50%) as compared to the vehicle control in *db/db* mice, whereas MD001 treatment showed no increase in liver weight or fat mass (Supplementary Fig. S8). These results strongly suggest that MD001 may efficiently decrease blood glucose and lipid levels without significant changes in body weight and hepatotoxicity.

### MD001 improves metabolic disorders in *db/db* mice

**Figure 6:**
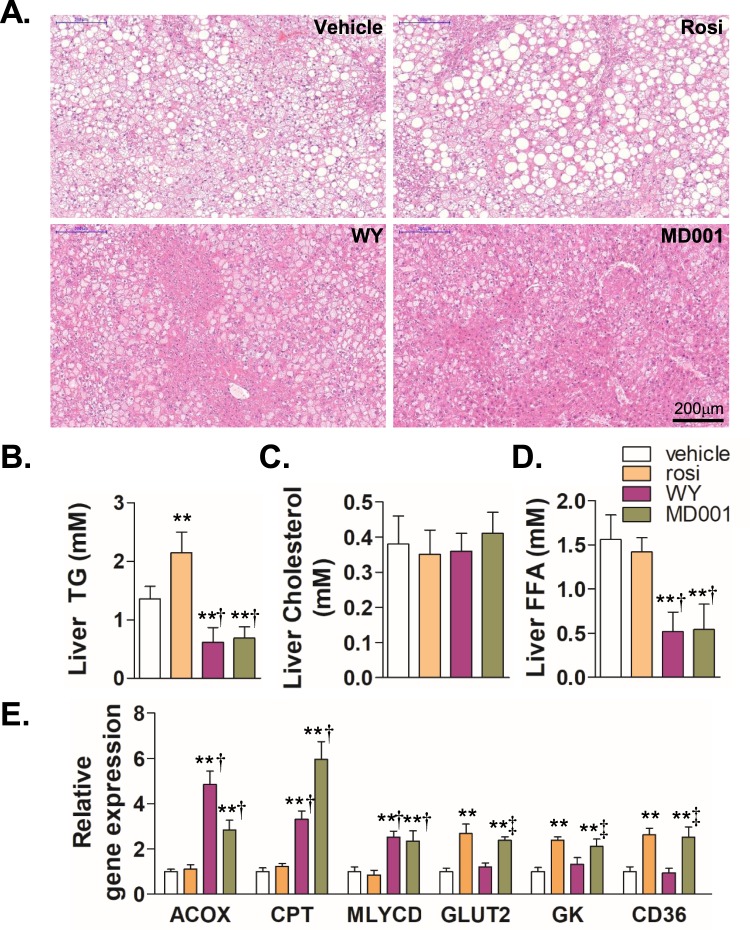
MD001 improves hepatic steatosis in *db/db* mice. (**A**) *db/db* mice were administered with vehicle, WY14643 (WY, 20 mg/kg), rosiglitazone (rosi, 20 mg/kg), or MD001 (20 mg/kg) as indicated for 4 weeks, and then livers were harvested for H&E staining (n = 5–6 per group). (**B**) liver TG, (**C**) cholesterol, and (**D**) free fatty acid levels were quantitated as described in the Methods section. (**E**) The relative gene expression levels of each gene from *db*/*db* mice were analysed by qRT-PCR. **, vs. vehicle; ^†^, vs. rosi; ^‡^, vs. WY. The data represent the mean ± S.D. ^**^P < 0.01, ^†^*P* < 0.01, and ^‡^*P* < 0.01.

Fatty liver is a complication associated with insulin resistance, obesity, and type 2 diabetes. While PPARα activation has been known to alleviate fatty liver by stimulating β-oxidation and reducing lipogenesis, the effect of activated PPARγ on hepatic steatosis is controversial^35,36^. We assessed whether MD001, as a dual agonist of PPARα/γ, may alleviate fatty liver in *db/db* mice. As shown in Fig. 6A, rosiglitazone exacerbated hepatic steatosis, as reported in a previous studies^35,36^. However, MD001 treatment resulted in the reduction in size and number of hepatic lipid droplets in a dose-dependent manner, suggesting that MD001 alleviated fatty liver (Fig. 6A). In addition, MD001 significantly reduced hepatic TG and FFA, but not cholesterol; rosiglitazone, on the other hand, failed to reduce hepatic TG (Fig. 6B–D). Quantitative RT-PCR and immunoblot analyses showed that MD001 significantly increased the expression of target genes of PPARα (*ACOX*, *CPT*, and *MLYCD*), and PPARγ (*GLUT2*, *GK*, and *CD36*) (Fig. 6E, Supplementary Fig. S9), but decreased the expression of genes associated with hepatic lipogenesis, including adipocyte determination and differentiation-dependent factor 1 (ADD1), acetyl-CoA carboxylase (ACC), and fatty acid synthase (FAS) (Supplementary Fig. S10). Thus, MD001 may reduce hepatic steatosis at least in part by stimulating β-oxidation or inhibiting hepatic lipogenesis.

**Figure 7:**
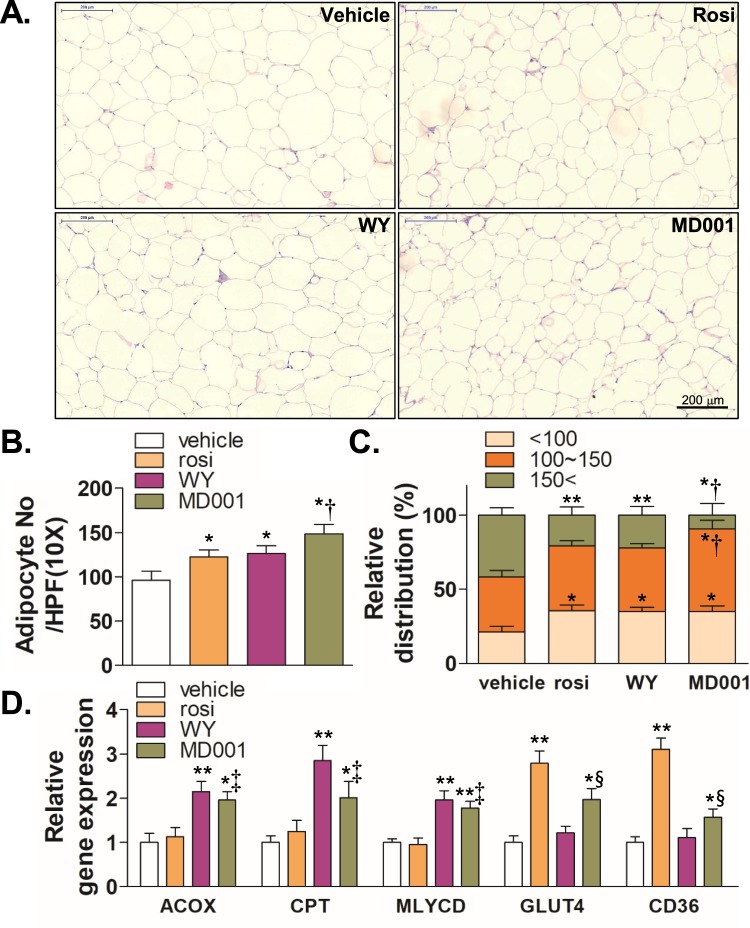
MD001 reduces fat cell size. (**A**) Adipose tissue from *db*/*db* mice was harvested and subjected to H&E staining (n = 5–6 per group). (**B**) The average number of fat cells per high- power field (HPF) was counted. (**C**) The relative distribution of fat cells was evaluated by size. (**D**) Relative gene expression was determined by qRT-PCR. * and **, vs. vehicle; ^†^, vs. rosi and WY; ^‡^, vs. rosi; ^§^, vs. WY. The data represent the mean ± S.D. *P < 0.05, **P < 0.01, ^†^*P* < 0.01, ^‡^*P* < 0.05, and ^§^*P* < 0.05.

Next, we examined the effect of MD001 on fat-cell size in *db/db* mice, as the progression of obesity induces increases in cell size and adipocyte number. Adipocyte size is one of the indicators of metabolic stresses such as inflammation, insulin resistance, and hyperlipidaemia^37,38^. While rosiglitazone and WY14643 significantly increased the number of fat cells below 100 μm in diameter, they significantly reduced the number of fat cells above 150 μm in diameter (Fig. 7A–C). Interestingly, MD001 significantly increased the number of medium fat cells (100~150 μm in diameter) as well as the number of small fat cells (<100 μm in diameter), whereas MD001 significantly reduced the number of large fat cells (>150 μm). In addition, qRT-PCR and immunoblot analyses showed that MD001 significantly increased the expression of *ACOX*, *CPT*, *GLUT4*, and *CD36*, indicating that MD001 may not only induce fatty acid and glucose uptake but also stimulate β-oxidation in adipose tissue at least in part (Fig. 7D, Supplementary Fig. S11). Furthermore, MD001 decreased the expression of inflammatory genes TNFα and MCP-1, as well as macrophage-marker genes CD11b and CD11c (Supplementary Fig. S12).

In addition, H&E staining of skeletal muscle revealed no differences between vehicle-, rosiglitazone-, WY14643-, and MD001-treated *db/db* mice (Supplementary Fig. S13a). Interestingly, qRT-PCR analysis of skeletal muscle showed that MD001 significantly increased the expression of *ACOX*, *CPT*, *MLYCD*, *GLUT4*, and lipoprotein lipase *(LPL)*, indicating that MD001 may increase glucose and fatty acid metabolism in skeletal muscle (Supplementary Fig. S13B). Moreover, H&E staining of adipose tissue, skeletal muscle, and liver from wild type C57BL/6J mice treated with vehicle, rosiglitazone, or MD001 showed no differences (Supplementary Fig. S14).

## Discussion

Type 2 diabetes and its related complications, including hypertension, arteriosclerosis, and diabetic retinopathy are recognised as serious problems in Westernized societies. The advantages of PPARα and PPARγ agonists in the treatment of metabolic syndrome have led to the development of PPARα/γ dual agonists. In this study, we synthesised derivatives of Int B and examined their potential roles as PPARα and PPARγ agonists. Of these, only MD001 enhanced the transcriptional activity of PPARα and PPARγ *in vitro*. Interaction with PPAR requires acidic hydrogen to act as a hydrogen-bonding donor on the interacting chemical compound. Compounds **4a** and **4b** lack acidic hydrogen, which may affect their stimulatory activities. Although MD002 bears acidic hydrogen (for hydrogen bonding) and a similar chemical structure, it failed to enhance the transcriptional activity of PPARα and PPARγ, which may be attributable to the different location of its methyl group. Further studies on the relevant structure-activity relationships (SARs) may reveal the mechanism involved.

*In vitro* binding assays revealed that MD001 exhibited a significantly higher binding affinity for PPARγ than PPARα (Supplementary Table S3). However, MD001 significantly lowered hyperlipidaemia, though the blood glucose lowering effect of MD001 was not as high as that of rosiglitazone, which may be attributable to the existence of a different regulatory mechanism that needs further elucidation. Therefore, the mechanism responsible for MD001-mediated lipid homeostasis is likely to be different from that of rosiglitazone. *In vitro* and *in vivo* experiments showed that MD001 induced glucose consumption and β-oxidation in the liver, adipose tissue, and skeletal muscle. In addition, MD001 increased the expression levels of PPARα and PPARγ target genes. These results further confirmed the effect of MD001 on blood glucose levels and hyperlipidaemia through the simultaneous activation of PPARα/γ. The currently-available PPARα/γ dual agonists are associated with PPARγ-related side-effects such as fluid retention and weight gain, limiting their application at higher doses for improved efficacy^39^. Although the blood glucose lowering effect of MD001 was lower than that of rosiglitazone, the MD001-mediated increase in fatty acid oxidation via PPARα activation suggests that MD001 may have favourable effects on hyperlipidaemia and obesity without inducing body weight gain, at least in part.

Hepatic steatosis, a common complication in obesity and type 2 diabetes, is closely associated with insulin resistance^40^. MD001 alleviated fatty liver by reducing TG and FFA levels in *db/db* mice, which was associated with increased expression levels of *ACOX*, *CPT*, and *MLYCD* and decreased expression levels of *ADD1*, *ACC*, and *FAS* by PPARα activation. In addition, hepatomegaly is commonly associated with fatty infiltration of the liver and increased serum ALT^41^. Rosiglitazone induced significant increases in blood ALT and fatty liver, resulting in hepatomegaly (Figs 5I and 6A, Supplementary Fig. S8). MD001-treated obese *db/db* mice showed significant improvement in fatty liver, with no signs of hepatomegaly-a common PPARα agonist-associated adverse effect in rodents^3,42^. Several PPARα/γ dual agonists, including ragaglitazar, are known to alleviate fatty liver without hepatomegaly^43^, though the underlying molecular mechanisms remain to be elucidated.

About 40% of patients with type 2 diabetes eventually suffer from kidney failure; PPAR agonists are known to have renoprotective effect^44^. Examination of kidney showed that MD001 as well as WY14643 and rosiglitazone significantly reduced the diameter of the glomerular capsule (Supplementary Fig. S15). In addition, reduction of haemoglobin (Hb) and haematocrit (HCT) levels in PPAR agonist-treated patients is often observed. The examination of RBC, Hb, and HCT showed that rosiglitazone significantly decreased RBC count, Hb, and HCT levels, whereas MD001 did not decrease RBC count, Hb, and HCT levels (Supplementary Fig. S16), suggesting that MD001 does not induce negative effects on blood profile components. A toxicology study revealed no differences in the white blood cell counts and haemoglobin. Liver and kidney toxicities were not observed in wild type C57/BL6 mice treated with 50 or 100 mg/kg MD001 for eight weeks (Supplementary Table S4). In addition, there were no significant differences in body weight change (data not shown).

In summary, we have demonstrated that MD001 improved glucose and lipid metabolism in *db/db* mice through PPARα and PPARγ activation. In addition, MD001 showed no severe adverse effects such as fatty liver, body weight gain, liver toxicity, and hepatomegaly commonly observed with previous PPAR agonists, thereby alleviating metabolic disorders. MD001 ameliorates abnormal lipid profiles through the consumption of excess lipids from peripheral tissues, instead of storage in adipose tissue. The development of MD001 as a PPARα/γ dual agonist targeting metabolic disease may help to overcome the limitations associated with previous PPAR agonists.

## Methods

### Chemical synthesis

2-methylbenzene-1,3,5-triol was purchased from Frontier Scientific Services, Inc. (Newark, DE, USA). Methyl phloroglucinol (2.0 g, 14.3 mmol), ethyl benzoyl acetate (5.50 g, 28.6 mmol, 2.0 eq.), trifluoroacetic acid (2 mL), and AcOH (40 mL) were added to a round-bottomed flask and the reaction mixture was heated to reflux for 12 h. The reaction mixture was evaporated under reduced pressure and water (20 mL) was added. The aqueous layer was extracted with EtOAc (50 mL) three times. The complete protocol for the synthesis of Int B isoform is described in Supplementary Information. After synthesis, each compound was dissolved in a solution of 10% diethylene glycol monoethyl ether (Gattefossé, Saint-Priest, France), and 20% polyoxyl 15 hydroxystearate (BASF, Florham Park, NJ, USA).

### Cell culture

Cells were maintained at 37 °C in a humidified chamber containing 5% CO_2_. HepG2 cells were cultured in Minimum Eagle’s medium (MEM) supplemented with 10% FBS and 1% penicillin and streptomycin. HEK293, C2C12, and 3T3-L1 cells were maintained in Dulbecco’s Modified Eagle’s Medium (DMEM) containing 10% FBS and 1% penicillin and streptomycin. Adipocyte differentiation was induced as previously described^45^. C2C12 cells were differentiated as previously described^46^.

### Transient transfection and luciferase activity assay

For transient transfection, cells were transfected with plasmid DNA constructs by polyethylenimine (PEI, Polysciences, Inc., Warrington, PA, USA) according to the manufacturer’s instructions. Transcription activity assays of PPARα and PPARγ in HEK293 cells were performed by co-transfection of HA-PPARα, HA-PPARγ, and peroxisome proliferator-response element (PPRE) reporter plasmid (PPRE-pk-Luc) or control reporter plasmid (pk-Luc) with *Renilla* vector for 24 h. Cells were treated with MD001, rosiglitazone (Sigma-Aldrich/Millipore, Burlington, MA, USA), or WY14643 (Sigma-Aldrich/Millipore) at indicated concentrations for 24 h. Luciferase activity was measured with the Dual-Luciferase Reporter Assay System (Promega, Madison, WI, USA) and quantified using GloMax (Promega, USA) according to the manufacturer’s protocol. Luciferase activity was normalised to *Renilla* luciferase activity. For the knockdown experiment, HepG2, 3T3-L1, and C2C12 cells were transfected with 20 nM of siRNA targeting PPARα and PPARγ (Invitrogen/Thermo Fisher Scientific, Carlsbad, CA, USA) using lipofectamine 2000 (Invitrogen/Thermo Fisher Scientific) for 48 h. Following transfection, cells were treated with vehicle, rosiglitazone, WY14643 or MD001 for 24 h.

### Quantitative reverse transcription PCR analysis

Total RNA was isolated using an RNeasy kit (Qiagen, Germantown, MD, USA) according to the manufacturer’s instructions. Complementary DNA (cDNA) was synthesised by reverse transcription using 0.5 μg of total RNA, and quantitative RT-PCR was performed using Power SYBR Green PCR Master Mix (ABI Thermo Fisher Scientific, Foster City, CA, USA) on a StepOne 48-well real-time PCR system (ABI/Thermo Fisher Scientific). GAPDH was used as an endogenous control. Primer sequences are summarized in Supplementary Table S1.

### Metabolic assay

For the fatty acid oxidation assay, cells were incubated in α-MEM (HyClone Laboratories, Inc., Logan, UT, USA) containing 0.1 mM palmitate (9,10-[^3^H]palmitate, 5 μci/mL) (PerkinElmer, Waltham, MA, USA) and 1% bovine serum albumin for 24 h. Following incubation, the medium was harvested and precipitated with 10% trichloroacetic acid (Sigma-Aldrich/Millipore), vortexed, and incubated for 20 min at room temperature, then centrifuged at 16000 × g for 10 min at 4 °C. The supernatants were transferred to a new tube and incubated in a scintillation vial containing 0.5 mL water at 60 °C for 12 h. After incubation, the vial was removed and radioactivity (^3^H_2_O in water) was quantified using a liquid scintillation counter (LKB Instruments, Victoria, Australia).

### *In vitro* glucose consumption assay

The glucose consumption assay was performed as previously described^23^. Briefly, cells were treated with MD001 or rosiglitazone for three days. The conditioned medium from cultured cells was harvested and assayed for glucose content using a Glucose Colorimetric Assay Kit II (BioVision Inc., Milpitas, CA, USA).

### Surface plasmon resonance (SPR) analysis

SPR analysis was performed using a Reichert SR7500DC (Reichert Technologies, Depew, NY, USA). HA-PPARα, HA-PPARβ/δ, and HA-PPARγ were purified as previously described^23^ and was immobilised on CMDH chips (Reichert Technologies) according to the manufacturer’s instructions. MD001 was loaded in a dose-dependent manner (31.25, 62.5, 125, 250, and 500 μM) for the SPR assay. The dissociation constant (K_D_) value was determined using the Scrubber2 program (Informer Technologies, Inc., Los Angeles, CA, USA).

### *In vivo* experiment

The Ajou University Animal Care and Use Committee approved all animal studies (IACUC2015-0001), and the experiment conformed to the Guide for the Care and Use of Laboratory Animals published by the United States National Institutes of Health. All experiments were performed in accordance with relevant guidelines and regulations. Six-week-old C57BLKS/J-*Lepr*^*db*^*/Lepr*^*db*^ or wild type C57BL/6J male mice were purchased from Orient Bio, Inc. (Seongnam, Korea) and acclimatised for one week. Mice were randomly grouped and orally administered with vehicle, WY14643 (20 mg/kg), rosiglitazone (20 mg/kg), or MD001 (5 mg/kg or 20 mg/kg) once a day for two months. For the oral glucose tolerance test (OGTT) and intraperitoneal insulin tolerance test (IPITT), mice were fasted for 12 h and were treated with sterile glucose (1 g/kg, Sigma-Aldrich/Millipore) or human insulin (1 unit/kg, Eli Lilly and Company, Indianapolis, IN, USA). Blood glucose levels were measured at the indicated time point using an OneTouch Ultra Blood Glucose Monitoring System (LifeScan, Inc., Milpitas, CA, USA). For the toxicity study, wild type C57BL/6J male mice were randomly grouped and orally administered with vehicle and MD001 (50 mg/kg or 100 mg/kg) once a day for two month. Blood cells, body weight change, and blood and urine metabolites were analysed for toxicity.

### Biochemical analysis

Livers and blood samples were collected from mice in each group. Serum and liver TG, FFA, and total cholesterol were measured using WAKO reagents (WAKO chemicals USA, Inc., Richmond, VA, USA) using Hitachi Clinical Analyzer 7180 (Hitachi High-Technologies GLOBAL, Tokyo, Japan). Serum concentrations of low-density lipoprotein (LDL) and high-density lipoprotein (HDL) were determined using Sekisui reagents (Sekisui Medical Co., Ltd., Tokyo, Japan)^47^. Plasma alanine aminotransferase (ALT) and aspartate aminotransferase (AST) levels using WAKO reagents (Wako Chemicals USA, Inc) were measured for analysis of hepatotoxicity.

### Tissue section and staining

Tissues specimens including liver, perigonadal adipose tissue, skeletal muscle, spleen, kidney, and heart, were collected from mice in each group, fixed in 10% formalin, and embedded into paraffin. Tissue sections (5 μm) were stained with haematoxylin and eosin (H&E).

### Statistical analysis

All data were analysed using GraphPad Prism 5.0 software (La Jolla, CA, USA). The results are expressed as mean ± SD or mean ± SEM. Statistical significance was calculated using one-way or two-way analysis of variance (ANOVA) with a post hoc Bonferroni multiple comparison test to compare the differences between groups. *P* < 0.05 was considered statistically significant.

## Supplementary information

Supplementary information

## Data Availability

All data generated or analysed during this study are included in this published article (and its Supplementary Information files).
