# Supplementary material for: MD001, a Novel Peroxisome Proliferator-activated Receptor α/γ Agonist, Improves Glucose and Lipid Metabolism
Source: Sci Rep. 2019 Feb 7;9:1656. doi: 10.1038/s41598-018-38281-0 (PMC6367362; doi:10.1038/s41598-018-38281-0)
Supplement: Supplementary file 1 — Supplementary information [file 41598_2018_38281_MOESM1_ESM.pdf]

MD001, a Novel Peroxisome Proliferator-activated Receptor α/γ Agonist, Improves Glucose and Lipid Metabolism

Seok-Ho Kim, Shin Hee Hong, Young-Joon Park, Jong-Hyuk Sung, Wonhee Suh, Kyeong Won Lee, Kiwon Jung, Changjin Lim, Jin-Hee Kim, Hyoungsu Kim, Kyong Soo Park, Sang Gyu Park

Supplementary Information

Supplementary Materials and Methods

Chemical synthesis. Methyl phloroglucinol (**1**) (2.0 g, 14.3 mmol), ethyl benzoyl acetate (5.50 g, 28.6 mmol, 2.0 eq.), trifluoroacetic acid (2 mL), AcOH (40 mL) was added to a round-bottomed flask and the reaction mixture was heated to reflux for 12 hrs. The reaction mixture was evaporated under reduced pressure and water (20 mL) was added. The aqueous layer was extracted with EtOAc (50 mL) three times. Combined organic layer was washed with water (10 mL) two times, dried over $MgSO_4$, and adsorbed onto silica gel. The mixture was purified by silica gel column chromatography (EtOAc : hexane = 1 : 2) to obtain two regioisomers, **2a** and **2b.**

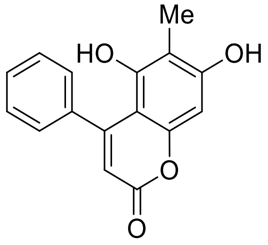

5,7-Dihydroxy-6-methyl-4-phenyl-2H-chromen-2-one (**2b**) yield: 14%; yellow solid; m.p. 274-276 °C; ^{1}H-NMR (300 MHz, DMSO-d_6): δ 10.46 (s, 1H), 8.87 (s, 1H), 7.37-7.31 (m, 5H), 6.44 (s, 1H), 5.74 (s, 1H), 1.98 (s, 3H); ^{13}C-NMR (75 MHz, DMSO-d_6): δ 159.9, 159.8, 156.3, 154.0, 153.9, 140.3, 127.6, 127.4, 127.3, 110.7, 107.7, 101.6, 94.7, 8.6; IR (ATR) ν_{max} 3151, 1680, 1597, 1560, 1365, 1338, 1126 cm^{-1}; HRMS (ESI) m/z calculated for $C_{16}H_{13}O_4$ $(M+H)^+$ 269.0814, detected 269.0815

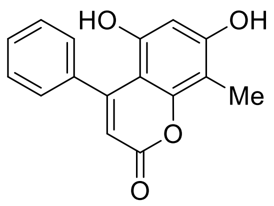

5,7-Dihydroxy-8-methyl-4-phenyl-2H-chromen-2-one (**2a**) yield: 19%; yellow solid. m.p. 210-212 °C; ^{1}H-NMR (500 MHz, DMSO-d_6): δ 10.32 (s, 1H), 9.81 (s, 1H), 7.37-7.29 (m, 5H), 6.29 (s, 1H), 5.72 (s, 1H), 2.08 (s, 3H); ^{13}C-NMR (75MHz, DMSO-d_6): δ 160.0, 159.6, 156.3, 154.3, 154.1, 139.9, 127.6, 127.3, 127.2, 109.6, 102.1, 100.6, 98.6, 7.7; IR (ATR) ν_{max} 3140, 1714, 1662, 1593, 1550, 1361, 1296, 1199, 1126, 1083 cm^{-1}; HRMS (ESI) m/z calculated for $C_{16}H_{13}O_4$ $(M+H)^+$ 269.0814, detected 269.0798

In the first reaction, compounds were highly insoluble and a considerable amount of loss was observed during the normal purification system. Instead of purification, we evaporated the crude mixture under reduced pressure and volatiles were removed under high vacuum conditions. The mixture was methylated using a known methylation procedure as follows: Methyl phloroglucinol (4.0 g, 24.6 mmol), ethyl benzoyl acetate (9.90 mL, 57.2mmol, 2.0 eq.), trifluoroacetic acid (4 mL), and AcOH (100 mL) were added to a round-bottomed flask and the reaction mixture was heated to reflux for 12 hrs. The reaction mixture was evaporated under reduced pressure and the crude reaction mixture was dissolved in acetone (150 mL); K_2CO_3 (11.7 g, 86.1 mmol, 3.5 eq.) and dimethyl sulfate (5.84 mL, 61.5 mmol, 2.5 eq.) was added portion wise at 0 ^{o}C and stirred at room temperature. Additional reagent was added until no starting material was remained. Acetone was removed under reduced pressure and the residue was dissolved in water (50 mL) / EtOAc (50 mL) to separate the organic layer. The Aqueous

layer was extracted with EtOAc (50 mL X 2) and the combined organic layer was washed with water (10 mL X 2). The Organic layer was dried with $MgSO_4$, evaporated under reduced pressure, and adsorbed onto silica gel. The reaction mixture was purified by silica gel column chromatography (EtOAc : hexane = 1 : 4, 5% DCM) to obtain two regioisomer **3a** and **3b.**

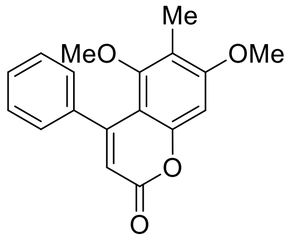

5,7-Dimethoxy-6-methyl-4-phenyl-2H-chromen-2-one (**3b**) yield: 20%; yellow solid m.p. 164-165 °C; ^{1}H-NMR (400MHz, $CDCl_3$): δ 7.39-7.35 (m, 5H), 6.68 (s, 1H), 6.04 (s, 1H), 3.87 (s, 3H), 2.93 (s, 3H) 2.05 (s, 3H); ^{13}C-NMR (150MHz, $CDCl_3$): δ 161.6, 160.8, 156.2, 155.2, 154.7, 138.7, 128.3, 127.8, 127.4, 117.4, 113.9, 106.6, 95.5, 60.9, 55.9, 8.7; IR (ATR) ν_{max} 2931, 1710, 1595, 1359, 1132, 1095 cm^{-1}; HRMS (ESI) m/z calculated for $C_{18}H_{17}O_4$ $(M+H)^+$ 297.1127, detected 297.1125

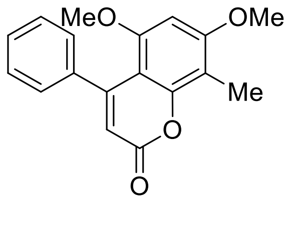

5,7-Dimethoxy-8-methyl-4-phenyl-2H-chromen-2-one (**3a**) yield: 31%; yellow solid m.p. 162-163 °C; ^{1}H-NMR (800 MHz, $CDCl_3$): δ 7.35-7.33 (m, 3H), 7.22-7.21 (m, 2H), 6.23 (s, 1H), 5.94 (s, 1H), 3.88 (s, 3H), 3.41 (s, 3H) 2.21 (s, 3H); ^{13}C-NMR (200 MHz, $CDCl_3$): δ 161.0, 160.9, 156.2, 155.7, 153.9, 140.1, 127.7, 127.3, 126.9, 112.2, 106.6, 103.2, 91.4, 55.8, 55.5,

7.7; IR (ATR) ν_{max} 2929, 1710, 1606, 1591, 1452, 1359, 1199, 1116 cm^{-1}; HRMS (ESI) m/z calculated for $C_{18}H_{17}O_4$ $(M+H)^+$ 297.1127, detected 297.1113

To a stirred solution of **3b** (1.30 g, 4.39 mmol), cinnamoyl chloride (1.83 g, 10.98 mmol, 2.5 eq.), in dichloroethane (DCE, 50 mL) and $TiCl_4$ (2.65 mL, 24.1 mmol, 5.5 eq.) were slowly added at 0 °C. The reaction mixture was heated to reflux for 48 h. MeOH was added carefully to quench $TiCl_4$ at 0 °C, followed by the addition of ice water. The organic layer was separated and the aqueous layer was extracted with EtOAc (50 mL X 3). The combined organic layer was washed with water (10 mL X 2). The organic layer was dried with $MgSO_4$ and evaporated under reduced pressure. The reaction mixture was purified by silica gel column chromatography (EtOAc: hexane = 1: 3, 5% DCM) to obtain **4b** (0.71 g, 1.67 mmol).

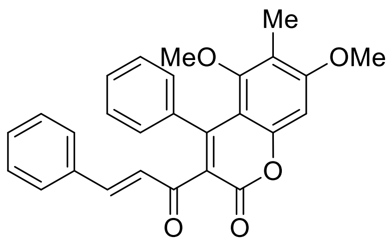

3-Cinnamoyl-5,7-dimethoxy-6-methyl-4-phenyl-2H-chromen-2-one (**4b**), yield: 38%; yellow solid m.p. 197-198 °C; ^{1}H-NMR (300 MHz, $CDCl_3$): δ 7.37-7.21 (m, 11H), 6.72 (s, 1H), 6.53 (d, J = 16.3 Hz, 1H), 3.91 (s, 3H), 2.95 (s, 3H), 2.06 (s, 3H); ^{13}C-NMR (100 MHz, $CDCl_3$): δ 192.0, 162.4, 158.7, 157.0, 154.2, 151.9, 145.5, 135.8, 134.4, 130.7, 128.8, 128.4, 128.1, 127.4, 126.8, 124.2, 118.1, 106.7, 95.5, 61.1, 56.1, 8.9; IR (ATR) ν_{max} 3408, 2935, 1708, 1591, 1448, 1361, 1197, 1136, 1099 cm^{-1}; HRMS (ESI) m/z calculated for $C_{27}H_{23}O_5$ $(M+H)^+$ 427.1545, detected 427.1525

To a stirred solution of **3a** (2.50 g, 8.44 mmol), cinnamoyl chloride (3.50 g, 21.1 mmol, 2.5 eq.), in DCM (120 mL) and $TiCl_4$ (5.09 mL, 46.4 mmol, 5.5 eq.) were slowly added at 0 °C. The reaction mixture was heated to reflux for 48 hrs. MeOH was carefully added to quench $TiCl_4$ at 0 °C, followed by addition of ice water. The organic layer was separated and the aqueous layer was extracted with EtOAc (50 mL X 3). The combined organic layer was washed with water (10 mL X 2). The organic layer was dried with $MgSO_4$ and evaporated under reduced pressure. The reaction mixture was purified by silica gel column chromatography (EtOAc: hexane = 1: 3, 5% DCM) to obtain **4b** (2.44 g, 5.74 mmol).

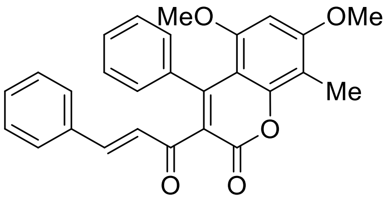

3-cinnamoyl-5,7-dimethoxy-8-methyl-4-phenyl-2H-chromen-2-one (**4a**), yield: 68%; yellow solid m.p. 224-226 °C; ^{1}H-NMR (300 MHz, $CDCl_3$): δ 7.36-7.08 (m, 11H), 6.72 (s, 1H), 6.53 (d, J = 16.3 Hz, 1H), 6.18 (s, 1H), 3.86 (s, 3H), 3.30 (s, 3H), 2.21 (s, 3H); ^{13}C-NMR (100 MHz, $CDCl_3$): δ 192.1, 161.7, 159.0, 157.4, 153.4, 152.8, 145.2, 137.1, 134.4, 130.6, 128.8, 128.4, 127.8, 127.4, 127.3, 127.1, 122.5, 106.7, 103.1, 91.8, 55.9, 55.8, 7.8; IR (ATR) ν_{max} 2933, 1710, 1608, 1591, 1357, 1201, 1136 cm^{-1}; HRMS (ESI) m/z calculated for $C_{27}H_{23}O_5$ $(M+H)^+$ 427.1545, detected 427.1533.

To a stirred solution of **4b** (0.29 g, 0.68 mmol), BBr_3 (1.29 mL, 13.6 mmol, 20.0 eq.) in DCE (30 mL) was slowly added at 0 °C. The reaction mixture was heated to reflux for 24 hrs. After the completion of the reaction, MeOH and ice cold water (20 mL) were carefully added to quench remaining BBr_3 at 0 °C. DCE was removed under reduced pressure and EtOAc was added. The organic layer was separated and the aqueous layer extracted with EtOAc (50 mL X 2). The combined organic layer was washed with water (10 mL X 2). The organic layer was dried with $MgSO_4$ and evaporated under reduced pressure. The reaction mixture was purified by silica gel column chromatography (EtOAc: hexane = 1: 2) to obtain **5b** (0.10 g, 0.251 mmol).

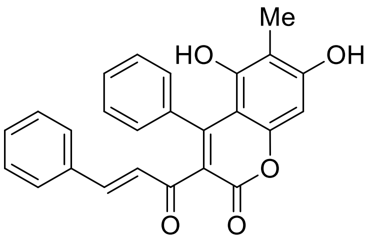

3-Cinnamoyl-5,7-dihydroxy-6-methyl-4-phenyl-2H-chromen-2-one (**5b**); yield: 37%; yellow solid m.p. 110-114°C; ^{1}H-NMR (800 MHz, $CDCl_3$): δ 8.00 (s, 1H), 7.46-7.44 (m, 3H), 7.41-7.38 (m, 5H), 7.31-7.30 (m, 4H), 6.89 (s, 1H), 6.62 (d, J = 16.1 Hz, 1H), 5.35 (s, 1H), 1.94 (s, 3H); ^{13}C-NMR (200 MHz, $CDCl_3$): δ 191.7, 160.5, 159.8, 153.8, 153.2, 152.0, 146.2, 134.0, 133.5, 130.9, 130.5, 129.8, 128.8, 128.6, 127.9, 126.7, 121.8, 109.6, 100.8, 96.5, 7.9; IR (thin film, neat) ν_{max} 3297, 1695, 1605, 1552, 1373, 1133, 1096 cm^{-1}; HRMS (ESI) m/z calculated for $C_{25}H_{19}O_5$ $(M+H)^+$ 399.1232, detected 399.1213

To a stirred solution of **4a** (0.50 g, 1.17 mmol), BBr_3 (2.22 mL, 23.4 mmol, 20.0 eq.) in DCE (50 mL) was slowly added at 0 °C. The reaction mixture was heated to reflux for 24 hrs. After

the completion of the reaction, MeOH and ice cold water (20 mL) were carefully added to quench remaining BBr_3 at 0 °C. DCE was removed under reduced pressure and EtOAc was added. The organic layer was separated and the aqueous layer was extracted with EtOAc (50 mL X 2). The combined organic layer was washed with water (10 mL X 2). The organic layer was dried with $MgSO_4$ and evaporated under reduced pressure. The reaction mixture was purified by silica gel column chromatography (EtOAc : hexane = 1 : 2) to obtain **5a** (0.19 g, 0.476 mmol).

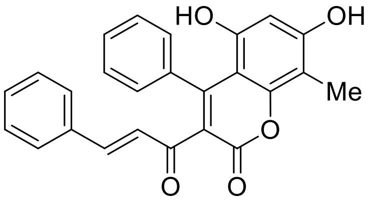

3-Cinnamoyl-5,7-dihydroxy-8-methyl-4-phenyl-2H-chromen-2-one (**5a**), yield: 41%; yellow solid m.p. 274-278 °C; ^{1}H-NMR (600 MHz, acetone-d_6): δ 9.31 (s, 1H), 8.58 (s, 1H), 7.55-7.54 (m, 2H), 7.53 (d, J = 16.3 Hz, 1H), 7.34-7.31 (m, 3H), 7.24-7.20 (m, 2H), 7.20-7.14 (m, 3H), 6.72 (s, 1H), 6.59 (d, J = 16.3 Hz, 1H), 6.30 (s, 3H), 2.16 (s, 3H); ^{1}H-NMR (400 MHz, DMSO-d_6): δ 10.46 (s, 1H), 9.82 (s, 1H), 7.63 (d, J = 6.0 Hz, 2H), 7.49 (d, J = 16.3 Hz, 2H), 7.39-7.37 (m, 3H), 7.22-7.16 (m, 5H), 6.63 (d, J = 16.3 Hz, 1H), 6.27 (s, 3H), 2.11 (s, 3H); ^{13}C-NMR (150 MHz, acetone-d_6): δ 193.5, 171.5, 161.2, 159.9, 156.6, 155.4, 153.8, 146.6, 138.6, 136.1, 131.8, 131.1, 129.7, 129.2, 128.9, 128.8, 128.5, 122.9, 104.6, 102.8, 100.4, 8.3; IR (ATR) ν_{max} 3373, 2931, 1716, 1604, 1361, 1138, 1099 cm^{-1}; HRMS (ESI) m/z calculated for $C_{25}H_{19}O_5$ $(M+H)^+$ 399.1232, detected 399.1223

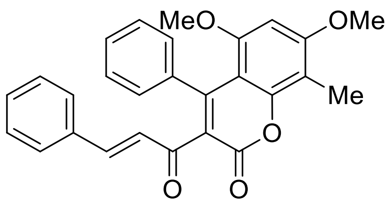

To a stirred solution of **5a** (53 mg, 0.133 mmol), K_2CO_3 (64 mg, 0.466 mmol, 3.5 eq.) in acetone (20 mL) and dimethyl sulfate (31 µL, 0.333 mmol. 2.5 eq.) were added portion wise at 0 ˚C and stirred at room temperature until no starting material was observed. Acetone was removed under reduced pressure and the residue was dissolved in water (50 mL)/EtOAc (50 mL) to separate the organic layer. The aqueous layer was extracted with EtOAc (50 mL) and the combined organic layer was washed with water (10 mL X 2). The organic layer was dried with MgSO4 and evaporated under reduced pressure. The reaction mixture was purified by silica gel column chromatography (EtOAc: hexane = 1: 3, 5% DCM) to obtain **4a** (35 mg, 0.082 mmol, 62% yield). The NMR spectrum of the synthesised compound matched with that of **4a**.

X-ray crystallographic data

For crystal structure determination of **4a**, crystal data for $C_{27}H_{22}O_5$ (M =426.44 g/mol): monoclinic, space group $P2_1/n$ (no. 14), a = 10.45830(10) Å, b = 16.2819(2) Å, c = 13.0437(2) Å, β = 98.1730(10)°, V = 2198.54(5) Å^3, Z = 4, T = 294.8(2) K, µ(CuKα) = 0.721 mm^{-1}, $Dcalc$ = 1.288 g/cm^3, 18734 reflections measured (8.74° ≤ 2Θ ≤ 153.292°), 4600 unique (R_{int} = 0.0208, R_{sigma} = 0.0169) which were used in all calculations. The final R_1 was 0.0413 (I > 2σ(I)) and wR_2 was 0.1196 (all data). CCDC 1540859 (Supplementary Table S5).

For crystal structure determination of **4b**, crystal data for $C_{18}H_{16}O_4$ (M = 296.31 g/mol): monoclinic, space group C2/c (no. 15), a = 18.0050(2) Å, b = 9.91663(13) Å, c =

16.84352(19) Å, β = 97.9087(12)°, V = 2978.79(7) Å^3, Z = 8, T = 295.3(3) K, μ(CuKα) = 0.764 mm^{-1}, $Dcalc$ = 1.321 g/cm^3, 12533 reflections measured (9.92° ≤ 2Θ ≤ 152.904°), 3102 unique (R_{int} = 0.0170, R_{sigma} = 0.0151) which were used in all calculations. The final R_1 was 0.0385 (I > 2σ(I)) and wR_2 was 0.1095 (all data). CCDC 1540860 (Supplementary Table S6).

Immunoblot

Cells or tissues were lysed with lysis buffer containing 20 mM HEPES, pH7.4, 150 mM NaCl, 1% TritonX-100, 1%sodium deoxycholate, 0.1% SDS,1 mM EDTA, and 10 mM Na4P2O7, 100 mM NaF, and 1 mM PMSF. The whole cell lysates (30 - 70 μg) were subjected to 10% SDS-PAGE, and then blotted with their specific antibodies (ACOX, PPARα and PPARγ, Santa Cruz Biotechnology, Santa Cruz, CA, USA; CPT, Proteintech, Rosemont, IL, USA; MLYCD, Sigma-Aldrich/Millipore; CD36, GLUT2 and GLUT4, Abcam, Cambridge, UK; GK, MERCK, Darmstadt, Germany). All blots were developed using the enhanced chemiluminescence kit (Pierce Biotechnology, Waltham, Massachusetts, USA).

Supplementary Table S1. Primers used for qRT-PCR

Primers	Sequences	Species
PPARα	F, 5′-CGGTGACTTATCCTGTGGTCC-3′	Human
	R, 5′-CCGCAGATTCTACATTCGATGTT-3′	
PPARγ	F, 5′-TACTGTCGGTTTCAGAAATGCC-3′	
	R, 5′-GTCAGCGGACTCTGGATTCAG-3′	
RXR	F, 5′-GACGGAGCTTGTGTCCAAGAT-3′	
	R, 5′-AGTCAGGGTTAAAGAGGACGAT-3′	
ACOX	F, 5′-ACTCGCAGCCAGCGTTATG-3′	
	R, 5′-AGGGTCAGCGATGCCAAAC-3′	
CPT	F, 5′-TCCAGTTGGCTTATCGTGGTG-3′	
	R, 5′-TCCAGAGTCCGATTGATTTTTGC-3′	
MLYCD	F, 5′-ACGTCCGGGAAATGAATGGG-3′	
	R, 5′-GTAACCCGTTCTAGGTTCAGGA-3′	
FATP	F, 5′-CTTCGATGGCTATGTCAGCGA-3′	
	R, 5′-AGCACGTCACCTGAGAGGTAG-3′	
mCAD	F, 5′-GCCACGGTAGAAACATTGGCT-3′	
	R, 5′-CTTTTGCAGTACCCAGCACCT-3′	
GK	F, 5′-CTGGGACAAGATAACTGGAGAGC-3′	
	R, 5′-TCAACGGTAGACTGGGTTCTTA-3′	
PEPCK	F, 5′-AAAACGGCCTGAACCTCTCG-3′	
	R, 5′-ACACAGCTCAGCGTTATTCTC-3′	
CD36	F, 5′-AAGCCAGGTATTGCAGTTCTTT-3′	
	R, 5′-GCATTTGCTGATGTCTAGCACA-3′	
FABP1	F, 5′-GTGTCGGAAATCGTGCAGAAT-3′	
	R, 5′-GACTTTCTCCCCTGTCATTGTC-3′	
GLUT2	F, 5′-CCATCTTCCTCTTTGTCAGCTT-3′	
	R, 5′-AAATTGCAGGTCCAATTGCT-3′	
GAPDH	F, 5′-AAGGTGAAGGTCGGAGTCAAC-3′	
	R, 5′-GGGGTCATTGATGGCAACAATA-3′	
PPARα	F, 5′-TCTTCACGATGCTGTCCTCCT-3′	Mouse
	R, 5′-CTATGTTTAGAAGGCCAGGC-3′	
GLUT2	F, 5′-TTCCAGTTCGGCTATGACATCG-3′	
	R, 5′-CTGGTGTGACTGTAAGTGGGG-3′	
GK	F, 5′-TGAACCTGAGGATTTGTCAGC-3′	
	R, 5′-CCATGTGGAGTAACGGATTTCG-3′	
CD36	F, 5′-ATGGGCTGTGATCGGAACTG-3′	
	R, 5′-GTCTTCCCAATAAGCATGTCTCC-3′	
LPL	F, 5′-TTGCCCTAAGGACCCCTGAA-3′	

	R, 5′-TTGAAGTGGCAGTTAGACACAG-3′
GLUT4	F, 5′-ACACTGGTCCTAGCTGTATTCT-3′
	R, 5′-CCAGCCACGTTGCATTGTA-3′
ACOX	F, 5′-TGTTAAGAAGAGTGCCACCAT-3′
	R, 5′-ATCCATCTCTTCATAACCAAATTT-3′
CPT	F, 5′-ACTCCTGGAAGAAGAAGTTCAT-3′
	R, 5′-AGTATCTTTGACAGCTGGGAC-3′
MLYCD	F, 5′-GCACGTCCGGGAAATGAAC-3′
	R, 5′-GCCTCACACTCGCTGATCTT-3′
GAPDH	F, 5′-ACCCCAGCAAGGACACTGAGCAAG-3′
	R, 5′-GGCTCCCTAGGCCCCTCCTGTTATT-3′

F, forward and R, reverse

Supplementary Table S2. Optimisation of Friedel-Crafts acylation.

substrate	Lewis acid	solvent	temperature	Yield (conversion)
6a	$SnCl_4$	CH_2Cl_2	0 °C to r.t.	31% (50%)
6a	BF_3	CH_2Cl_2	0 °C to reflux	No reaction
6a	$TiCl_4$	CH_2Cl_2	0 °C to r.t.	42% (60%)
6a	$TiCl_4$	CH_2Cl_2	Reflux	68% (>95%)
6b	$TiCl_4$	CH_2Cl_2	Reflux	No reaction
6b	$TiCl_4$	DCE	Reflux	38%

Supplementary Table S3. K_D values for MD001 compound using SPR assay

	K_D (μM)
PPARα	9.55±0.8
PPARβ/δ	N.B*
PPARγ	0.14±0.03

*, No binding up to 500 μM; K_D values are presented as mean ± standard error of the mean

Supplementary Table S4. Laboratory findings in control and MD001-treated mice

	Control	MD001 (50 mg/kg)	MD001 (100 mg/kg)
WBC (/mm^3)	8859.5 (± 1028.2)	9351.4 (± 638.5)	9057.4 (± 857.1)
Haemoglobin (g/dL)	11.7 (± 1.5)	12.1 (± 0.6)	12.3 (± 1.8)
BUN (mg/dL)	26.7 (± 4.3)	31.3 (± 5.1)	28.5 (± 3.6)
Creatinine (mg/dL)	0.4 (± 0.2)	0.3 (± 0.3)	0.5 (± 0.2)
AST (IU/L)	117 (± 58.5)	128 (± 63.8)	129 (± 41.2)
ALT (IU/L)	43.5(± 21.5)	50.8 (± 31.3)	53.7 (± 29.4)

Results are expressed as the mean (± SD).

ALT, alanine transaminase; AST, aspartate transaminase; BUN, blood urea nitrogen; WBC, white blood cells.

Supplementary Table S5. Crystal data and structure refinement for 4a

Identification code	exp_524
Empirical formula	$C_{27}H_{22}O_5$
Formula weight	426.44
Temperature/K	294.8(2)
Crystal system	monoclinic
Space group	$P2_1/n$
a/Å	10.45830(10)
b/Å	16.2819(2)
c/Å	13.0437(2)
α/°	90
β/°	98.1730(10)
γ/°	90
Volume/Å^3	2198.54(5)
Z	4
ρ_{calc}g/cm^3	1.288
μ/mm^{-1}	0.721
F(000)	896.0
Crystal size/mm^3	0.127 × 0.071 × 0.068
Radiation	CuKα (λ = 1.54184)
2Θ range for data collection/°	8.74 to 153.292
Index ranges	$-12 \leq h \leq 13$, $-18 \leq k \leq 20$, $-16 \leq l \leq 14$

Reflections collected	18734
Independent reflections	4600 [R_{int} = 0.0208, R_{sigma} = 0.0169]
Data/restraints/parameters	4600/0/292
Goodness-of-fit on F^2	1.049
Final R indexes [I>=2σ (I)]	R_1 = 0.0413, wR_2 = 0.1147
Final R indexes [all data]	R_1 = 0.0461, wR_2 = 0.1196
Largest diff. peak/hole / e Å^{-3}	0.25/-0.23

Supplementary Table S6. Crystal data and structure refinement for 3b

Identification code	exp_525
Empirical formula	$C_{18}H_{16}O_4$
Formula weight	296.31
Temperature/K	295.3(3)
Crystal system	monoclinic
Space group	C2/c
a/Å	18.0050(2)
b/Å	9.91663(13)
c/Å	16.84352(19)
α/°	90
β/°	97.9087(12)
γ/°	90
Volume/Å^3	2978.79(7)
Z	8
ρ_{calc}g/cm^3	1.321
μ/mm^{-1}	0.764
F(000)	1248.0
Crystal size/mm^3	0.178 × 0.115 × 0.086
Radiation	CuKα (λ = 1.54184)

2Θ range for data collection/°	9.92 to 152.904
Index ranges	$-22 \leq h \leq 22$, $-12 \leq k \leq 12$, $-21 \leq l \leq 15$
Reflections collected	12533
Independent reflections	3102 [R_{int} = 0.0170, R_{sigma} = 0.0151]
Data/restraints/parameters	3102/0/202
Goodness-of-fit on F^2	1.045
Final R indexes [I>=2σ (I)]	R_1 = 0.0385, wR_2 = 0.1066
Final R indexes [all data]	R_1 = 0.0407, wR_2 = 0.1095
Largest diff. peak/hole / e $Å^{-3}$	0.12/-0.18

Supplementary Captions

***Supplementary Figure S*1.** X-ray crystallographic structure of 3b **(A)** and 4a **(B)**. Displacement ellipsoids are drawn at 50% probability level.

***Supplementary Figure S*2.** HEK293 cells were transiently co-transfected with human HA-PPARα **(A)** and HA-PPARγ **(B)** expression vectors and reporter plasmid (PPRE-pk-Luc) or control reporter plasmid (pk-Luc) with *Renilla* vector for 24 h. Then, cells were treated with a variety of compounds (each 20 μM) for 24 h. Int B compound was treated as positive control. Luciferase activity was measured as described in the Methods section. Data represent the mean ± SD of three independent experiments.

***Supplementary Figure S*3.** Knock-down of PPARα and PPARγ using si-RNA. HepG2 cells were transfected with control si-RNA, PPARα si-RNA (A), or PPARγ si-RNA (20 nM) (B) for 48 h and whole cell lysates (60 μg) were prepared and subjected to immunoblotting. Tubulin was used as loading control. Whole scan of the western blot is available in supplementary data.

***Supplementary Figure S*4.** Knock-down of PPARα using si-RNA. Control si-RNA or PPARα si-RNA (20 nM) was transfected to HepG2 (A), 3T3-L1 (B), and C2C12 (C) for 48 h. Whole cell lysates (60 μg) were prepared and subjected to immunoblotting. Tubulin was used as loading control. Whole scan of the western blot is available in supplementary data.

***Supplementary Figure S*5.** MD001 enhances glucose tolerance by increasing insulin sensitivity in *db/db* mice. **(A)** OGTT was performed, and the area under the curve (AUC) was calculated (n = 5-6 in each group). **(B-C)** After intraperitoneal injection of insulin (0.75 U/kg), blood glucose level was measured as described in the Methods section (n= 5-6 in each group), and AUC was calculated. (D) The expression level of PEPCK and G6Pase in *db/db* mice was examined by qRT-PCR in the liver treated with vehicle, rosiglitazone (rosi, 20 mg/kg), WY14643 (WY, 20 mg/kg) or MD001 (20 mg/kg) (n = 5-6 per group) once a day for 60 days. ‡ and §, vs. vehicle; *, vs. WY; †, vs. time 0. The data represent the mean ± SD. *$P < 0.05$, §$P < 0.05$, †$P < 0.05$, and ‡$P < 0.01$.

Supplementary Figure **S6.** MD001 has no influence on food intake of mice. **(A)** Wild type C57/BL6 mice were orally administered with vehicle, rosiglitazone (20 mg/kg), or MD001 (20 mg/kg) once a day for 60 days. The change in their body weight was evaluated and presented as gain of body weight per group (n = 5 - 6 in each group). **(B)** The 3-day food intake in wild type C57/BL6 mice or *db/db* mice during the administration of the indicated drug was measured three times (n = 5 - 6 in each group). The data represent the mean ± SD.

Supplementary Figure **S7.** MD001 shows no influence on blood metabolites of wild type C57/BL6 mice. **(A-H)** Wild type C57/BL6 mice were administered with vehicle, rosiglitazone (20 mg/kg), or MD001 (20 mg/kg) (n = 5 in each group) for 60 days, and their blood metabolites were measured. **(B)** Blood glucose level was examined during the administration of the

indicated drug (n = 5 in each group). The data represent the mean ± SD.

***Supplementary Figure* S8.** Average tissue weight of *db/db* mice treated with PPAR agonists. Tissue weight was evaluated in *db/db* mice administered once a day for 60 days with vehicle, WY14643 (WY, 20 mg/kg), rosiglitazone (rosi, 20 mg/kg) or MD001 (n = 5-6 per group). Gon, gonadal. The data represent the mean ± SD (n = 5-6 per group). *, vs. vehicle. *$P < 0.05$.

***Supplementary Figure* S9.** The expression level of ACOX, CPT, MLYCD, GLUT2, GK, and CD36 was examined by immunoblot in the liver treated with vehicle, rosiglitazone (rosi, 20 mg/kg), WY14643 (WY, 20 mg/kg) or MD001 (20 mg/kg) (n = 5-6 per group) once a day for 60 days. Tubulin was used as loading control. Whole scan of the western blot is available in supplementary data.

***Supplementary Figure* S10.** The expression level of ADD1, ACC, and FAS in *db/db* mice was examined by qRT-PCR in the liver treated with vehicle, rosiglitazone (rosi, 20 mg/kg), WY14643 (WY, 20 mg/kg) or MD001 (20 mg/kg) (n = 5-6 per group) once a day for 60 days. The data represent the mean ± SD. †, vs. vehicle; **, vs. rosi. **$P < 0.01$ and †$P < 0.05$.

***Supplementary Figure* S11.** The expression level of ACOX, CPT, MLYCD. GLUT4, and CD36 in *db/db* mice was examined by immunoblotting in the adipose tissue treated with vehicle, rosiglitazone (rosi, 20 mg/kg), WY14643 (WY, 20 mg/kg) or MD001 (20 mg/kg) (n =

5-6 per group) once a day for 60 days. Whole scan of the western blot is available in supplementary data.

Supplementary Figure **S12.** MD001 decreases the expression of inflammatory and macrophage-marker genes. The expression level of TNFα, MCP-1, CD11b, and CD11c was examined by qRT-PCR in the liver of *db/db* mice treated with vehicle, rosiglitazone (rosi, 20 mg/kg), WY14643 (WY, 20 mg/kg) or MD001 (20 mg/kg) (n = 5-6 per group) once a day for 60 days. The data represent the mean ± SD. *, vs. vehicle. *$P < 0.05$.

Supplementary Figure **S13.** MD001 increases the expression of fatty acid oxidation genes in *db/db* mice. *db/db* mice were orally administered with vehicle, rosiglitazone (20 mg/kg), WY14643 (WY, 20 mg/kg) or MD001 (20 mg/kg) for 60 days (n = 5 in each group). **(A)** Representative images of H&E staining. Skeletal muscles isolated from *db/db* mice treated with PPAR agonists were fixed with 10% formalin and subjected to H&E staining. **(B)** Total RNA was isolated from skeletal muscle, and qRT-PCR was performed to examine the expression of PPAR target genes. **, vs. vehicle; †, vs. rosi; ǂ, vs. WY. **$P < 0.01$, †$P < 0.05$, and ǂ$P < 0.05$.

Supplementary Figure **S14.** Histological analysis of wild type C57/BL6 mice treated with PPAR agonists. Wild type C57/BL6 mice were orally administered with vehicle, rosiglitazone (rosi, 20 mg/kg) or MD001 (20 mg/kg) once a day for 60 days (n = 5 in each group), and liver

(A), skeletal muscle **(B)**, and adipose tissue **(C)** were isolated and subjected to H&E staining. Inset, adipocyte number per high-power field (HPF) under 20x magnification was counted and presented as bar graph. The data represent the mean ± SD.

***Supplementary Figure* S15.** MD001 significantly reduced diameter of glomerular capsule in *db/db* mice. Mice were orally administered with vehicle, rosiglitazone (20 mg/kg), WY14643 (WY, 20 mg/kg) or MD001 (20 mg/kg) once a day for 60 days (n = 5 in each group). **(A-B)** Representative images of glomerular capsule (n=5 in each group) were shown, and diameter of glomerular capsule was presented as mean ± SEM. **, vs. vehicle. ** $P < 0.01$.

***Supplementary Figure* S16.** Haemodilution parameters after 60 days treatment with vehicle, rosiglitazone (20 mg/kg), WY14643 (WY, 20 mg/kg) or MD001 (5 mg/kg, 20 mg/kg) (n=5 in each group) in *db/db* mice. **(A)** RBC count, **(B)** haemoglobin (Hb), and **(C)** haematocrit (HCT) are shown. The data represent the mean ± SEM. *, vs. vehicle. * $P < 0.05$.

^{1}H NMR and ^{13}C NMR spectra

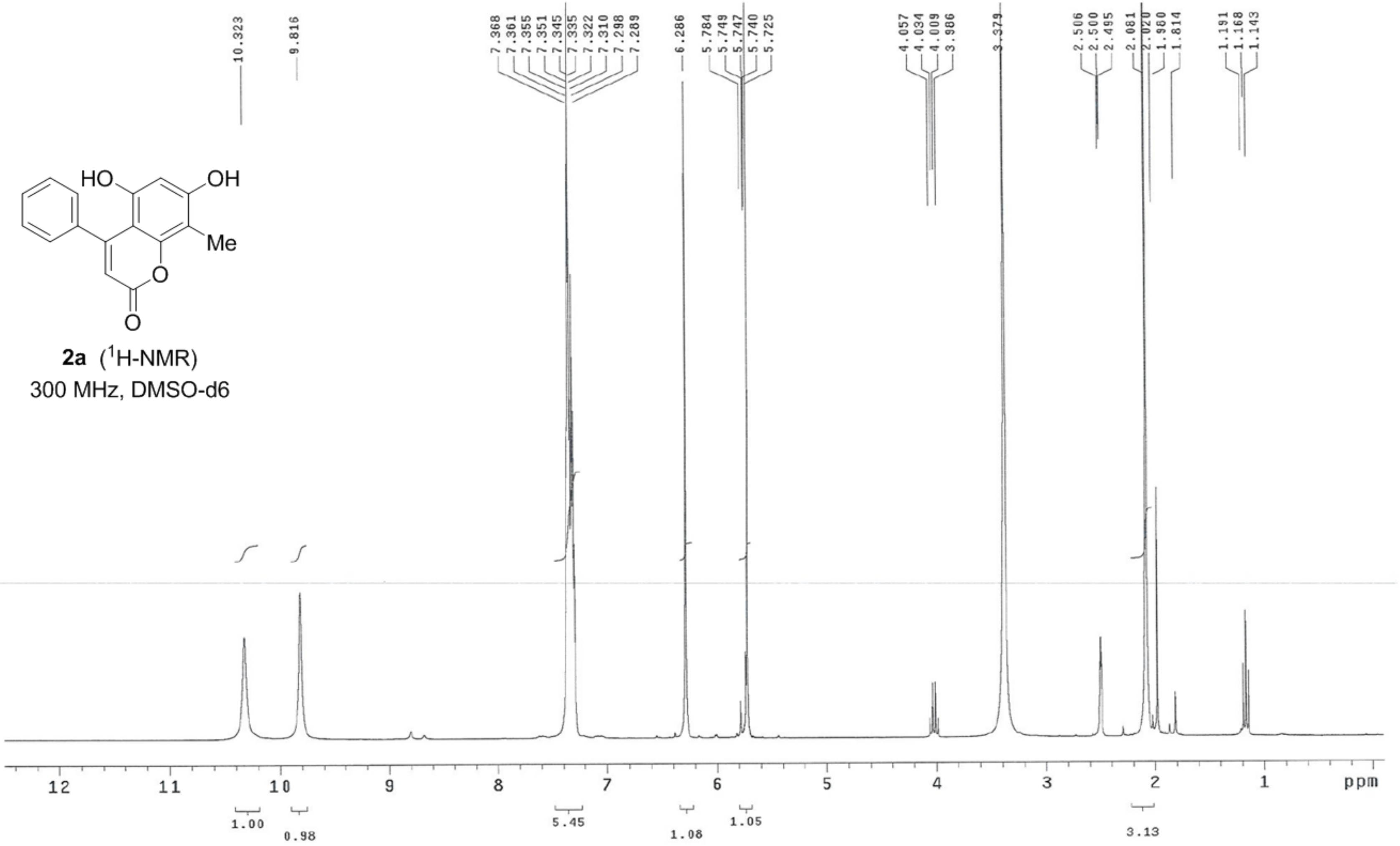

2a (^{1}H-NMR)
300 MHz, DMSO-d6

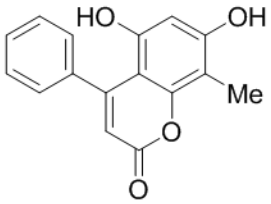

2a (^{13}C-NMR)
75 MHz, DMSO-d6

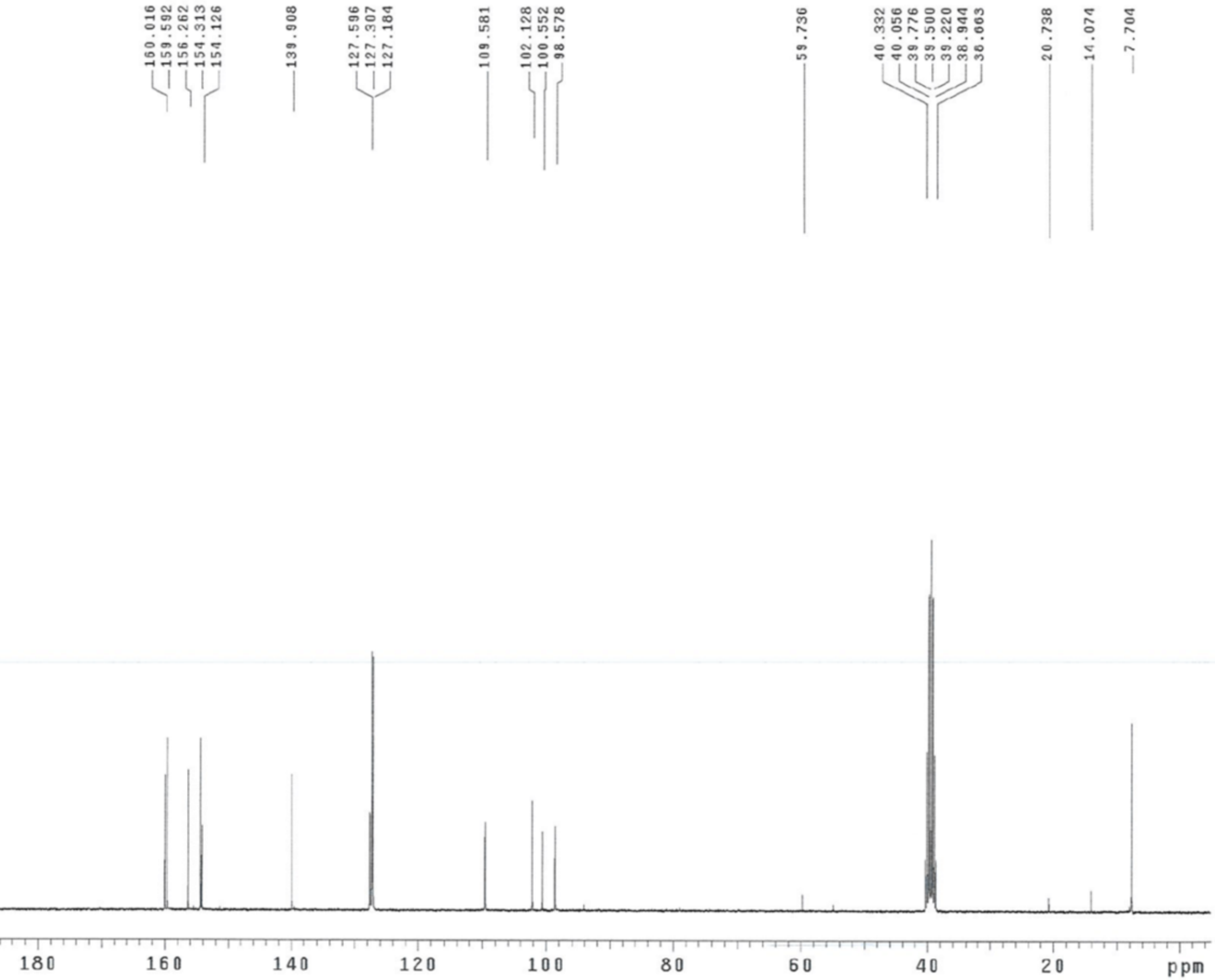

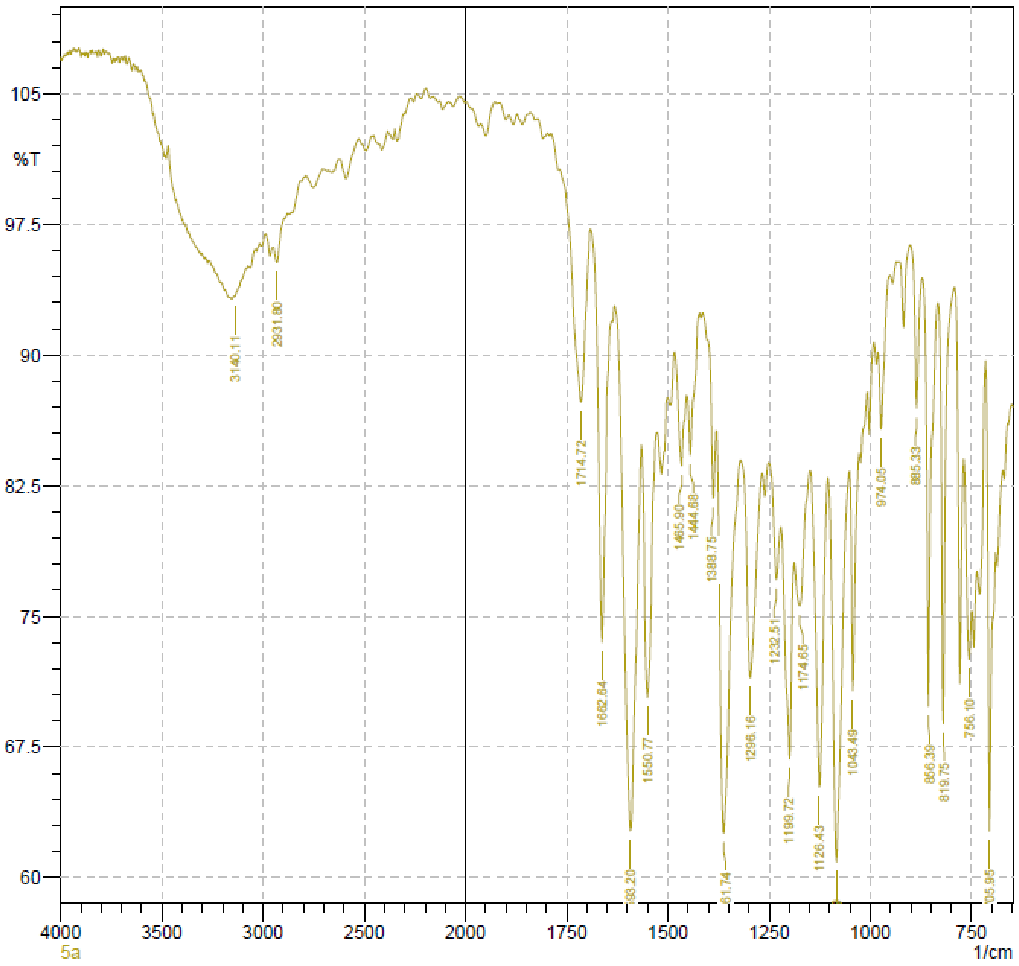

No.	Peak	Intensity	Corr. Inte	Base (H)	Base (L)	Area	Corr. Are
1	705.95	62.666	22.755	715.59	690.52	3.183	1.225
2	756.1	72.503	5.548	767.67	750.31	2.128	0.337
3	819.75	68.833	24.452	833.25	792.74	2.514	1.324
4	856.39	69.847	23.91	869.9	835.18	2.386	1.382
5	885.33	86.939	8.369	900.76	871.82	0.985	0.389
6	974.05	85.755	5.524	981.77	950.91	1.348	0.282
7	1043.49	70.726	12.952	1051.2	1028.06	2.455	0.69
8	1083.99	60.907	22.05	1103.28	1053.13	7.029	2.975
9	1126.43	65.187	17.958	1147.65	1105.21	5.416	2.012
10	1174.65	75.597	4.339	1188.15	1149.57	4.005	0.437
11	1199.72	66.818	11.718	1220.94	1190.08	4.24	1.063
12	1232.51	77.105	4.257	1249.87	1222.87	2.614	0.253
13	1296.16	71.468	12.055	1319.31	1269.16	5.453	1.519
14	1361.74	62.556	22.564	1379.1	1321.24	7.572	3.419
15	1388.75	81.782	5.461	1413.82	1381.03	1.805	0.141
16	1444.68	84.253	4.822	1454.33	1421.54	1.794	0.296
17	1465.9	83.633	5.054	1483.26	1454.33	1.823	0.349
18	1550.77	70.317	14.6	1564.27	1529.55	3.919	1.477
19	1593.2	62.696	25.434	1631.78	1566.2	7.789	4.353
20	1662.64	73.519	20.775	1691.57	1639.49	3.223	1.934
21	1714.72	87.289	10.77	1768.72	1693.5	2.008	1.611
22	2931.8	95.294	1.402	2949.16	2893.22	0.872	0.136
23	3140.11	93.345	0.077	3142.04	3111.18	0.869	0.011

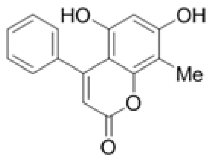

2a

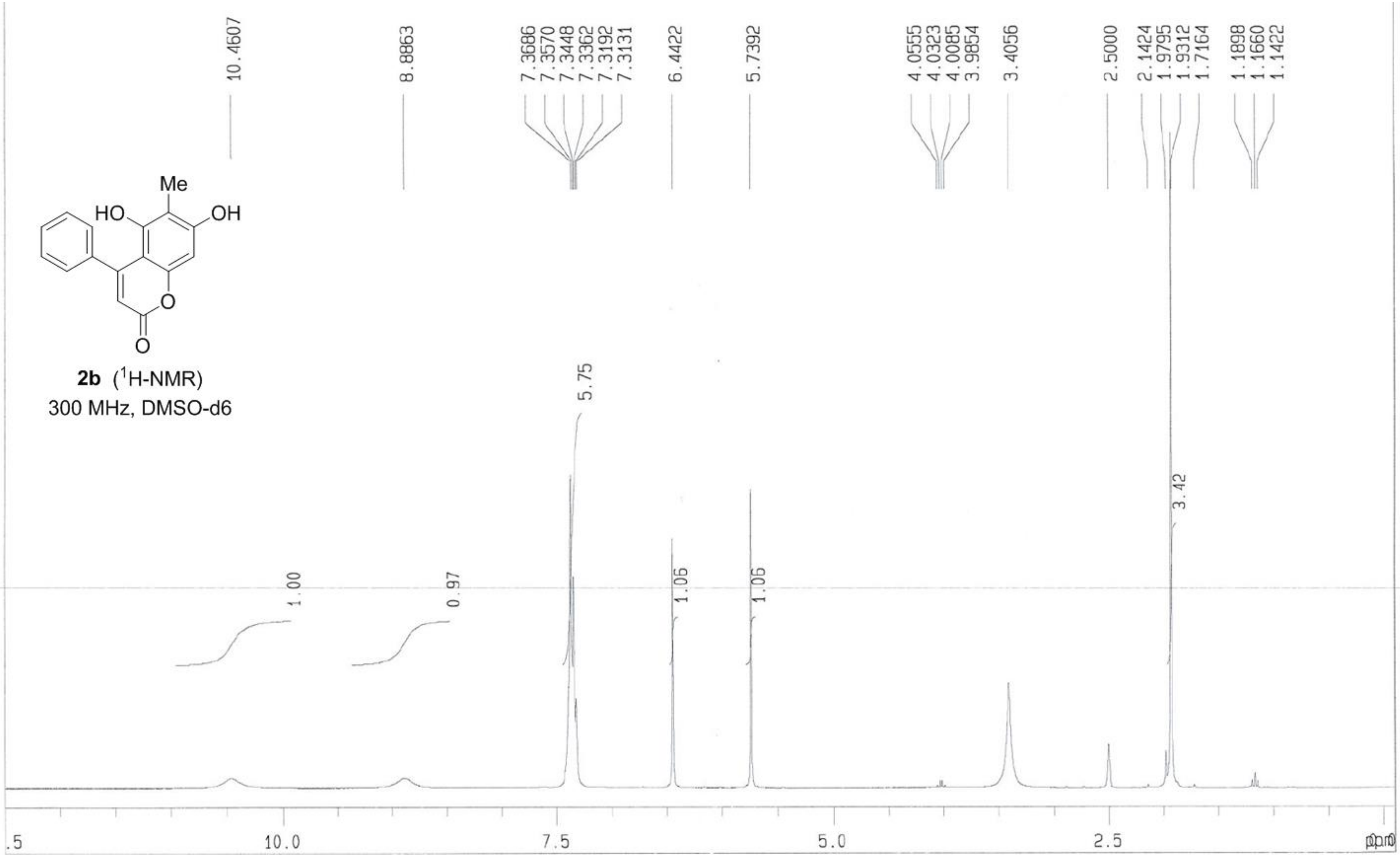
Me
HO
OH
O
O
2b (¹H-NMR)
300 MHz, DMSO-d6
10.4607
8.8863
7.3686
7.3570
7.3448
7.3362
7.3192
7.3131
6.4422
5.7392
4.0555
4.0323
4.0085
3.9854
3.4056
2.5000
2.1424
1.9795
1.9312
1.7164
1.1898
1.1660
1.1422
1.00
0.97
5.75
1.06
1.06
3.42
.5
10.0
7.5
5.0
2.5
ppm

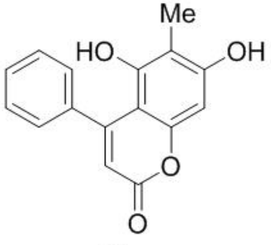

2b (^{13}C-NMR)
75 MHz, DMSO-d6

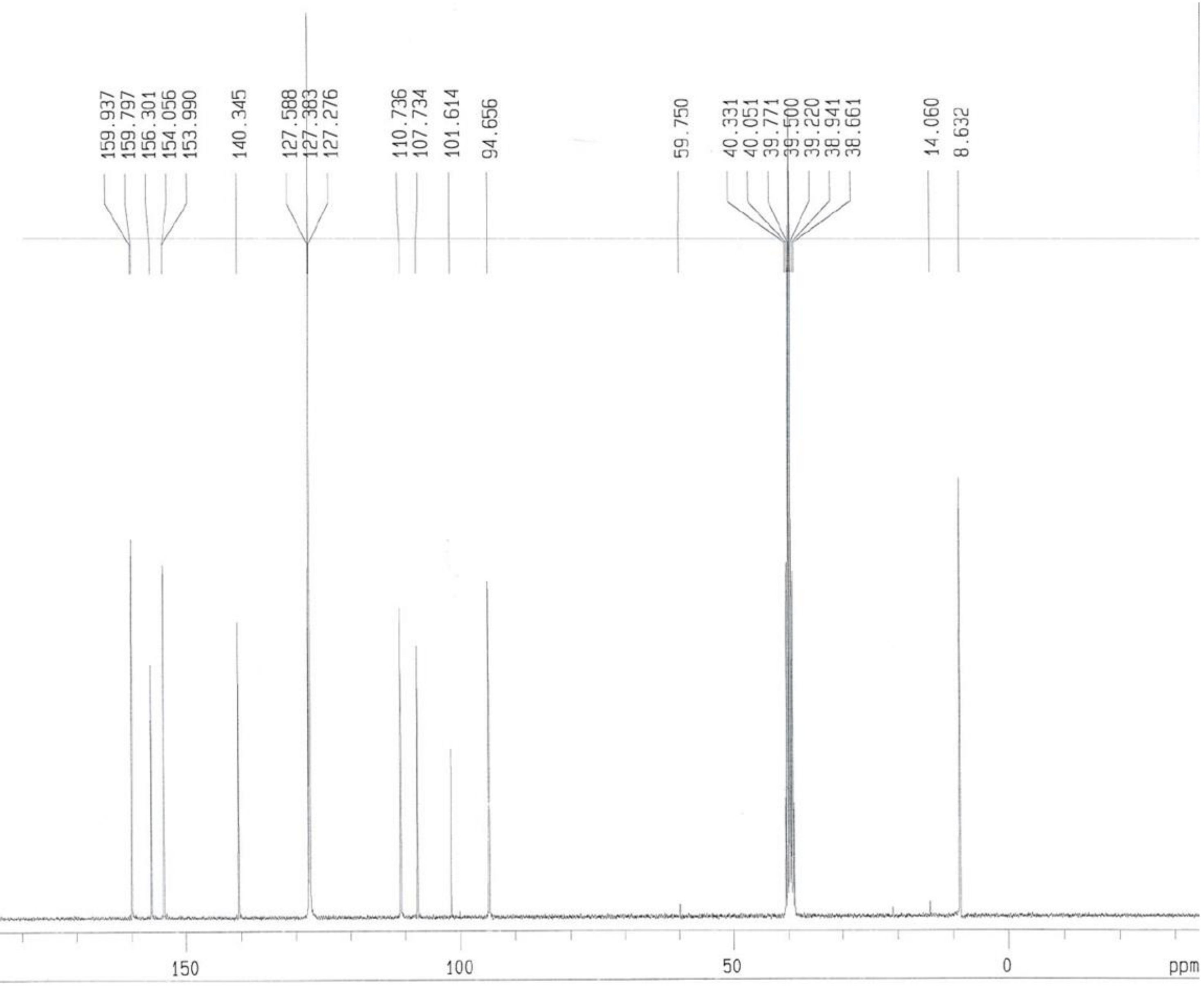

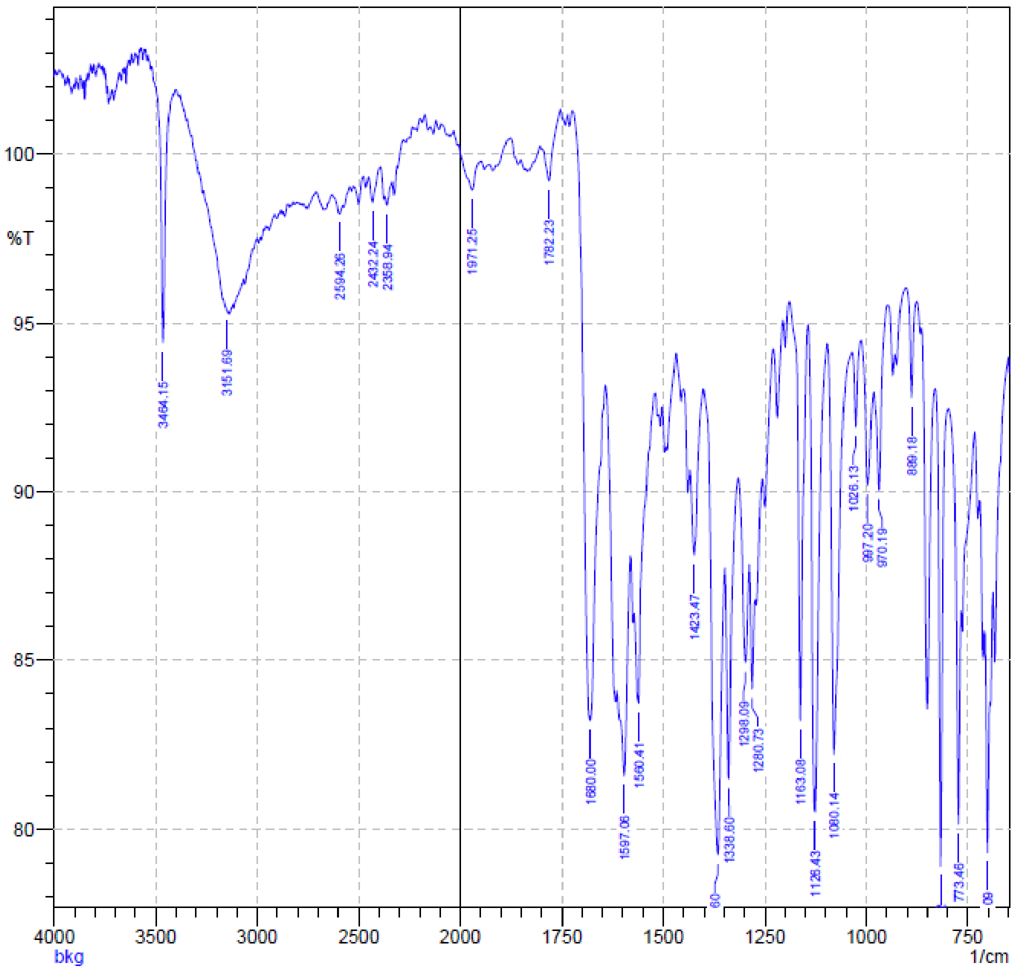

No.	Peak	Intensity	Corr. Inte	Base (H)	Base (L)	Area	Corr. Are
1	702.09	79.305	5.359	709.8	696.3	1.117	0.151
2	773.46	80.146	7.444	798.53	767.67	1.7	0.201
3	817.82	78.906	13.906	831.32	798.53	1.7	0.63
4	889.18	92.787	3.051	902.69	875.68	0.61	0.112
5	970.19	90.053	3.805	981.77	948.98	1.054	0.208
6	997.2	90.195	3.518	1012.63	981.77	1.082	0.211
7	1026.13	91.92	2.365	1035.77	1012.63	0.67	0.082
8	1080.14	82.225	12.077	1095.57	1035.77	2.77	1.232
9	1126.43	80.51	14.163	1141.86	1097.5	2.298	1.227
10	1163.08	83.209	12.035	1190.08	1143.79	1.721	0.753
11	1280.73	84.158	3.164	1288.45	1273.02	1.018	0.11
12	1298.09	84.936	3.823	1315.45	1288.45	1.59	0.239
13	1338.6	81.49	7.077	1348.24	1317.38	1.936	0.394
14	1365.6	79.24	9.885	1400.32	1350.17	3.465	1.188
15	1423.47	88.118	3.37	1435.04	1402.25	1.445	0.231
16	1560.41	83.734	3.743	1570.06	1521.84	2.451	0.175
17	1597.06	81.574	4.426	1612.49	1581.63	2.35	0.345
18	1680	83.215	13.622	1724.36	1643.35	3.106	2.066
19	1782.23	99.214	1.344	1797.66	1753.29	-0.032	0.11
20	1971.25	98.944	1.022	2011.76	1950.03	0.112	0.124
21	2358.94	98.488	0.143	2360.87	2335.8	0.124	0.001
22	2432.24	98.567	0.855	2451.53	2401.38	0.214	0.094
23	2594.26	98.235	0.33	2630.91	2580.76	0.329	0.03
24	3151.69	95.362	0.042	3169.04	3149.76	0.383	0.001
25	3464.15	94.414	7.5	3498.87	3415.93	0.117	0.793

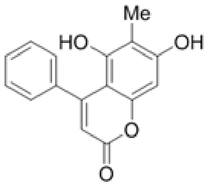

2b

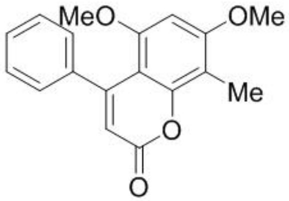

3a (^{1}H-NMR)
800 MHz, $CDCl_3$

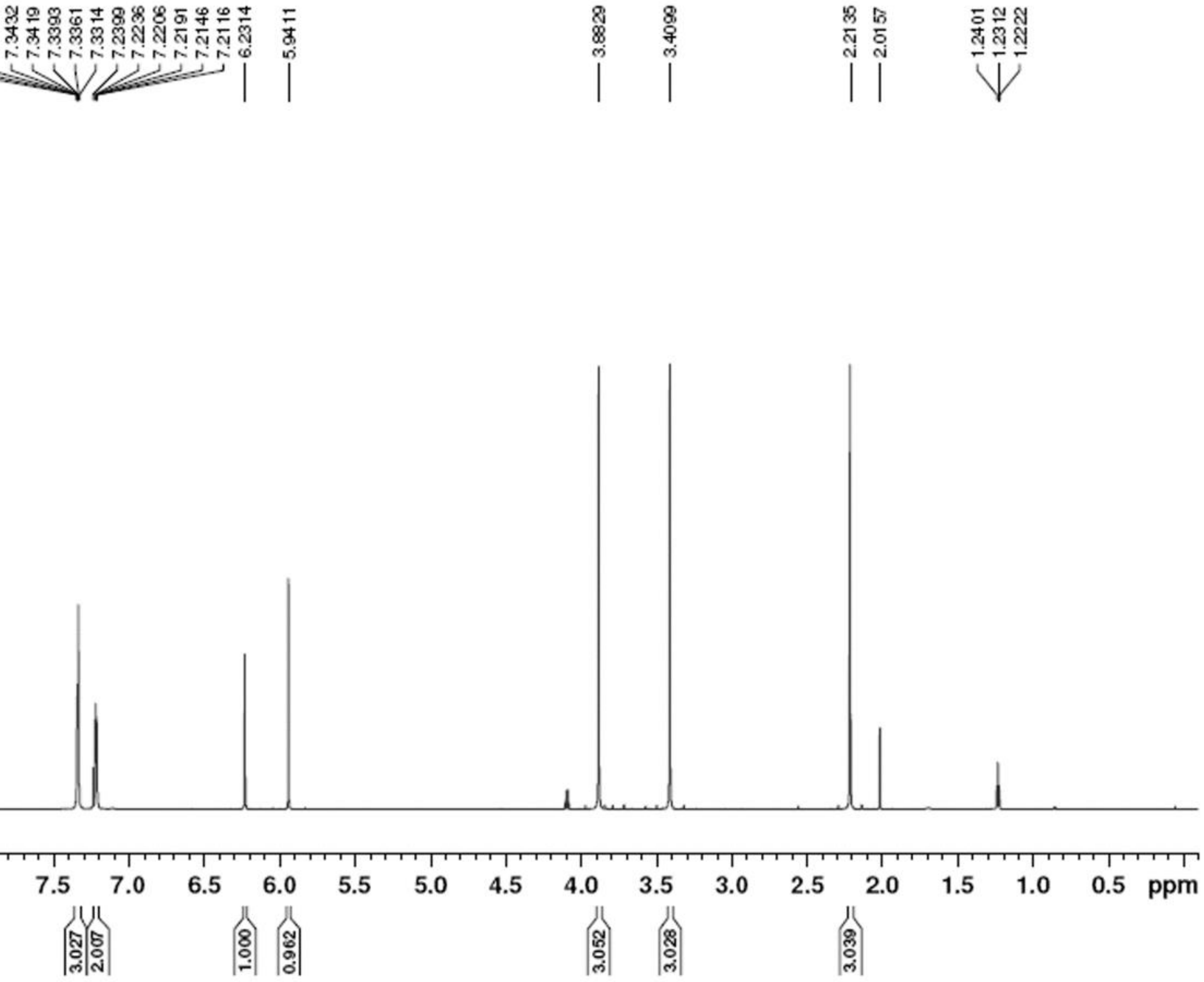

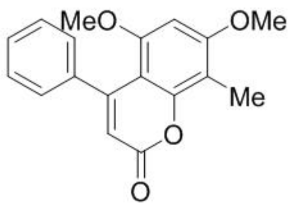

3a (^{13}C-NMR)
200 MHz, $CDCl_3$

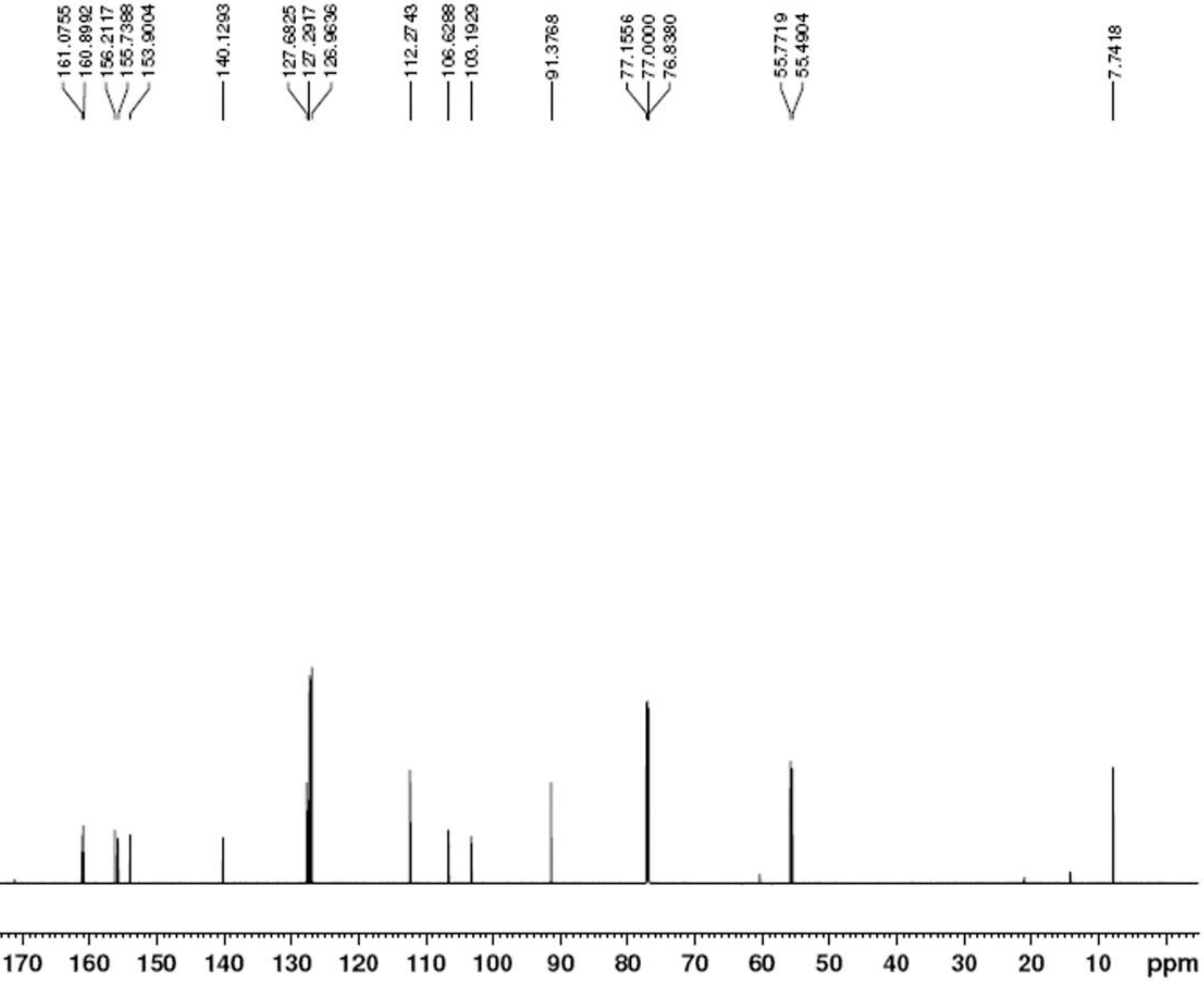

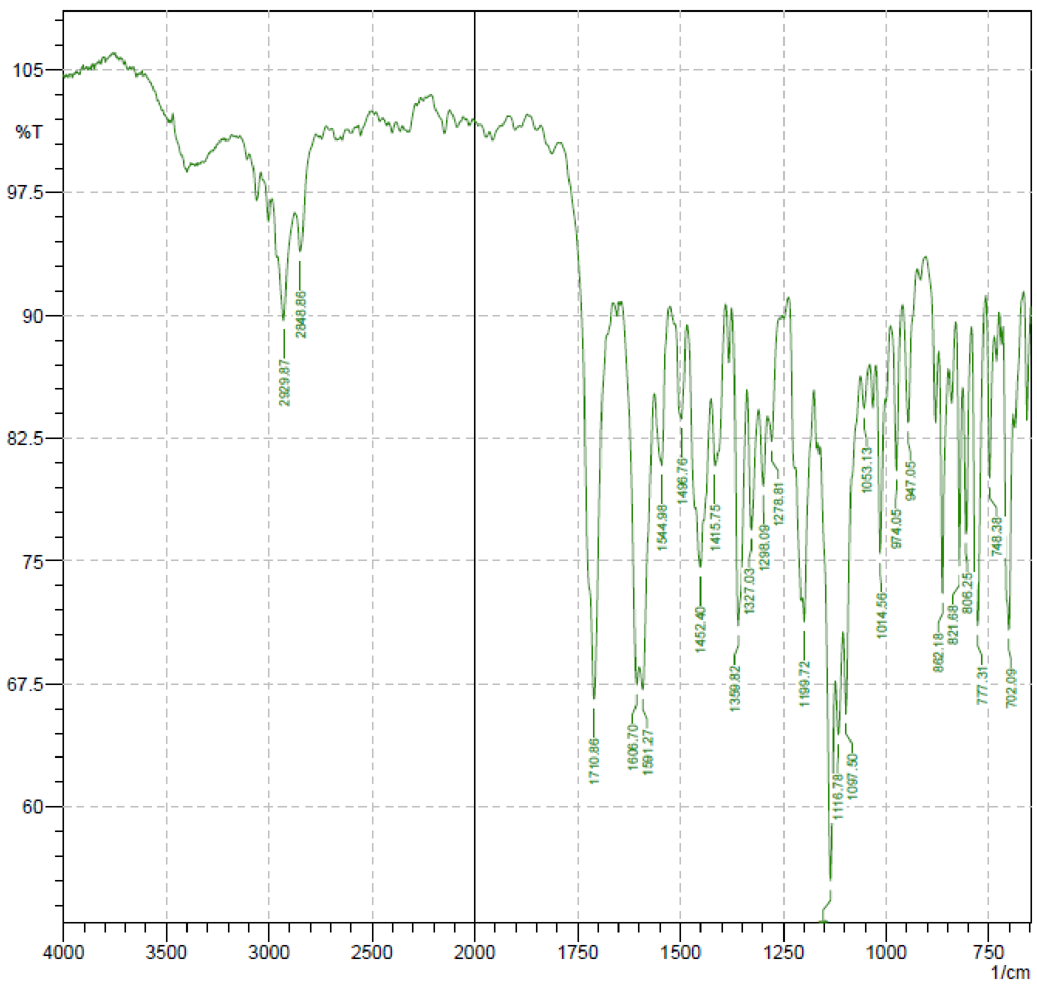

No.	Peak	Intensity	Corr. Inte	Base (H)	Base (L)	Area	Corr. Are
1	702.09	70.821	15.621	715.59	690.52	2.801	1.235
2	748.38	80.077	10.016	758.02	736.81	1.411	0.438
3	777.31	71.05	5.465	781.17	758.02	2.146	0.142
4	806.25	76.662	10.296	813.96	792.74	1.681	0.445
5	821.68	74.728	12.661	831.32	813.96	1.408	0.409
6	862.18	73.028	14.336	871.82	848.68	2.085	0.712
7	947.05	83.48	7.985	960.55	923.9	1.946	0.575
8	974.05	80.542	9.523	989.48	960.55	1.816	0.495
9	1014.56	75.461	10.546	1024.2	1002.98	1.93	0.52
10	1053.13	84.32	2.271	1062.78	1039.63	1.551	0.111
11	1097.5	65.666	7.069	1103.28	1062.78	4.408	0.331
12	1116.78	64.401	4.159	1122.57	1105.21	3.036	0.265
13	1134.14	55.489	16.111	1159.22	1124.5	6.033	1.495
14	1199.72	71.287	3.27	1203.58	1176.58	2.818	0.121
15	1278.81	82.325	3.52	1288.45	1259.52	1.955	0.175
16	1298.09	79.585	4.455	1309.67	1288.45	1.843	0.243
17	1327.03	76.95	7.892	1336.67	1311.59	2.34	0.528
18	1359.82	71.042	17.232	1373.32	1338.6	3.673	1.678
19	1415.75	80.832	5.677	1425.4	1390.68	2.593	0.65
20	1452.4	74.65	3.205	1462.04	1444.68	2.052	0.169
21	1496.76	83.701	5.76	1514.12	1485.19	1.857	0.459
22	1544.98	80.851	6.973	1562.34	1527.62	2.505	0.568
23	1591.27	67.148	5.038	1598.99	1564.27	4.344	0.391
24	1606.7	67.437	4.153	1643.35	1600.92	4.3	0.205
25	1710.86	66.588	27.798	1789.94	1664.57	8.086	5.567
26	2848.86	93.894	3.63	2875.86	2771.71	1.046	0.433
27	2929.87	89.71	4.852	2958.8	2877.79	2.545	0.728

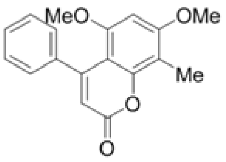

3a

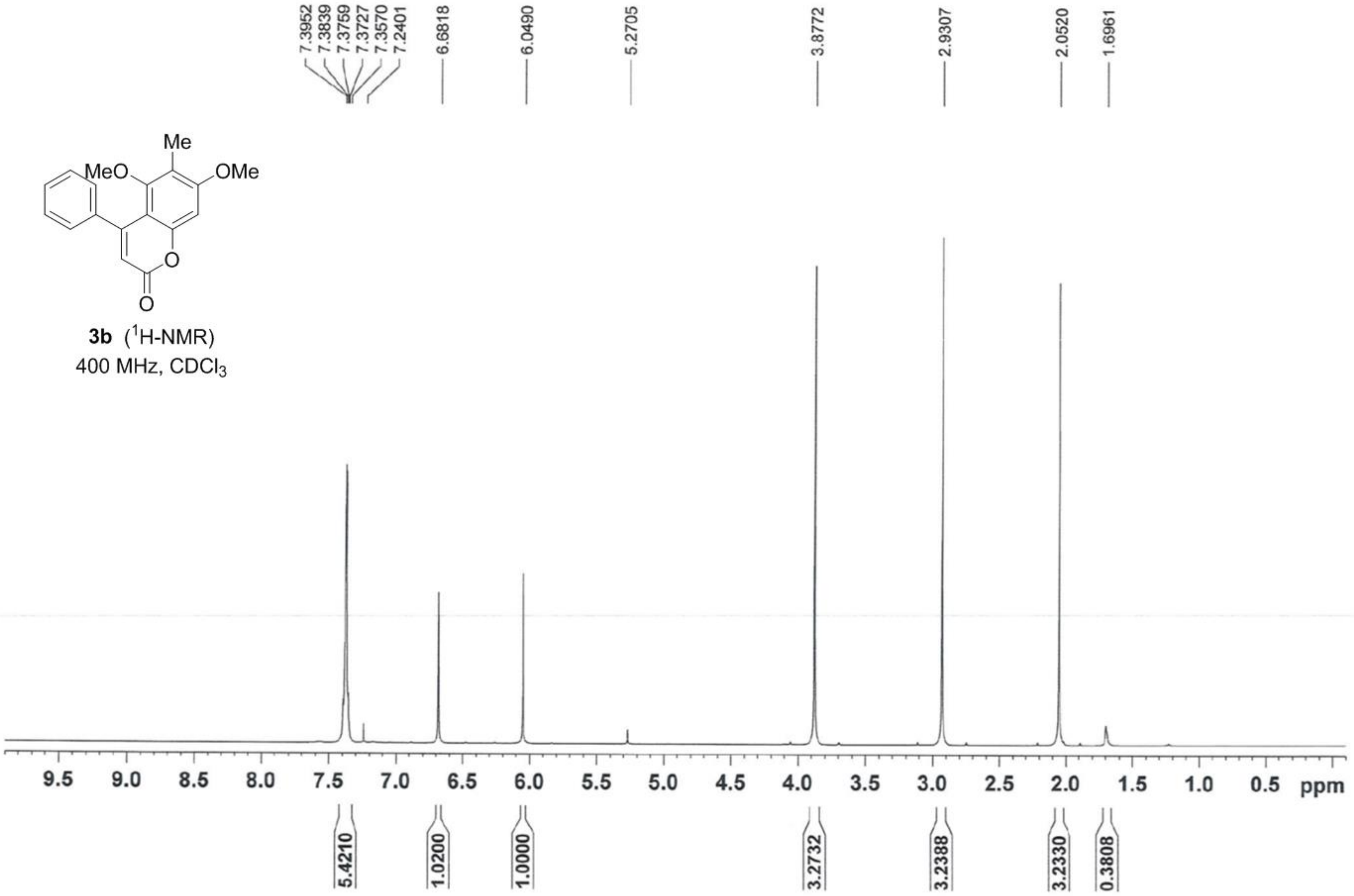

3b (^{1}H-NMR)
400 MHz, $CDCl_3$

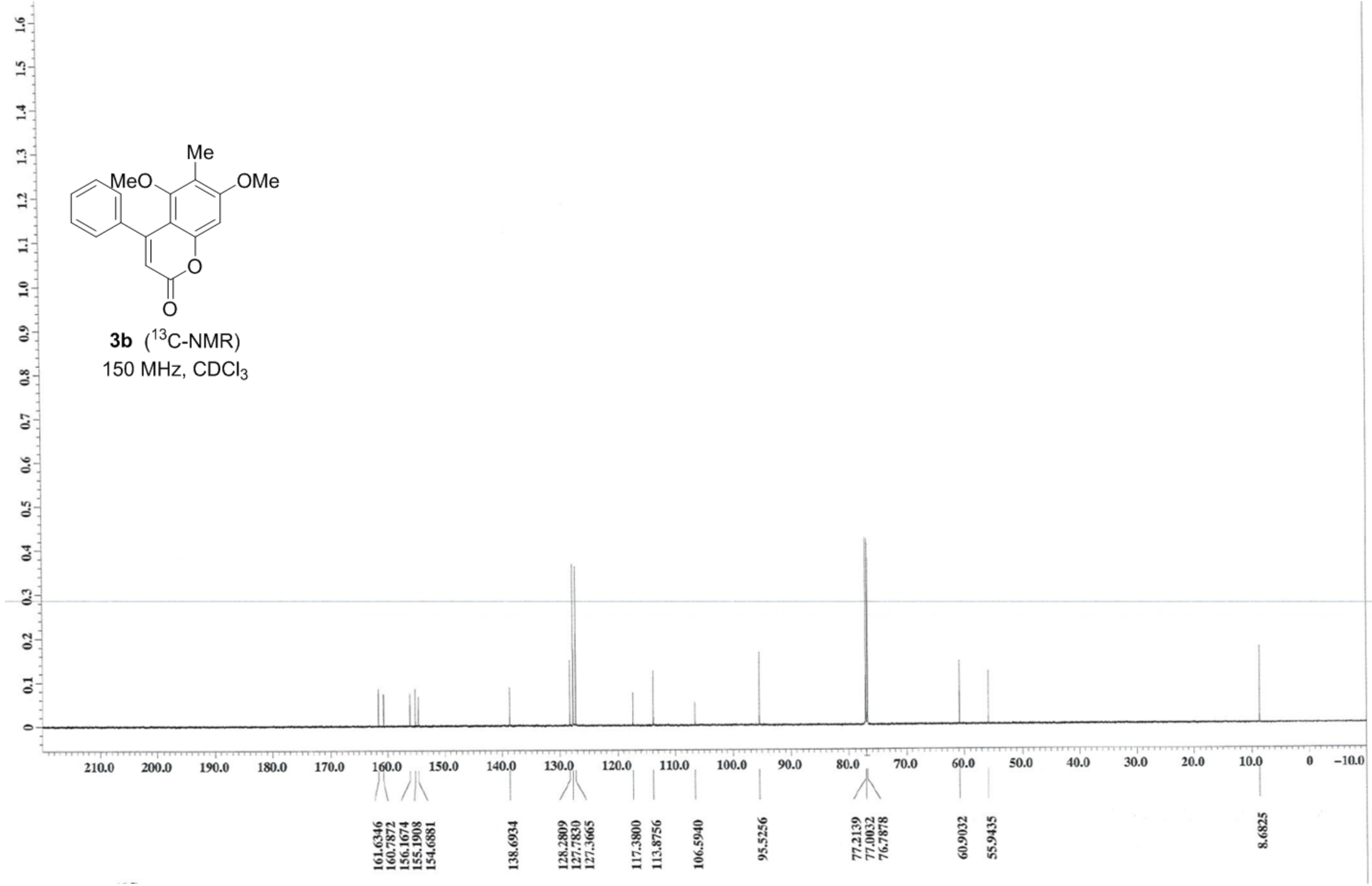
Me
MeO
OMe
O
O
3b (13C-NMR)
150 MHz, CDCl3
161.6346
160.7872
156.1674
155.1908
154.6881
138.6934
128.2809
127.7830
127.3665
117.3800
113.8756
106.5940
95.5256
77.2139
77.0032
76.7878
60.9032
55.9435
8.6825

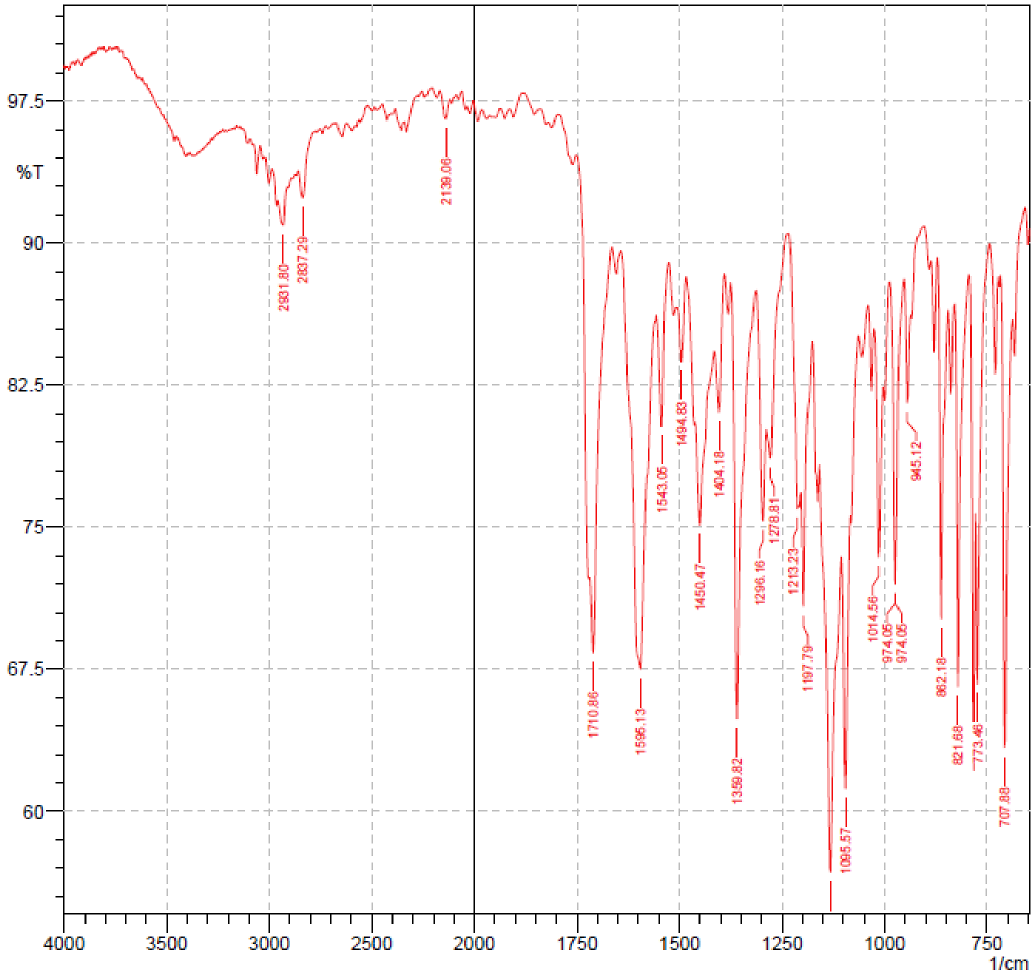

No.	Peak	Intensity	Corr. Inte	Base (H)	Base (L)	Area	Corr. Are
1	707.88	63.35	23.982	715.59	688.59	3.062	1.408
2	773.46	66.718	9.372	777.31	744.52	2.831	0.299
3	821.68	66.591	20.557	829.39	794.67	3.152	1.158
4	862.18	70.161	17.754	869.9	846.75	2.229	0.869
5	945.12	81.576	5.688	952.84	935.48	1.242	0.205
6	974.05	72.001	16.009	987.55	952.84	2.904	0.982
7	974.05	72.001	16.009	987.55	952.84	2.905	0.982
8	1014.56	73.435	10.411	1024.2	1004.91	1.979	0.488
9	1095.57	61.205	12.758	1103.28	1083.99	3.229	0.747
10	1132.21	56.789	18.774	1157.29	1105.21	8.982	2.592
11	1197.79	70.831	7.706	1203.58	1176.58	2.795	0.326
12	1213.23	75.984	4.444	1234.44	1205.51	2.508	0.206
13	1278.81	78.656	2.878	1286.52	1234.44	3.509	0.147
14	1296.16	75.354	7.082	1313.52	1288.45	2.403	0.442
15	1359.82	64.897	22.704	1373.32	1315.45	5.845	2.502
16	1404.18	81.074	5.105	1415.75	1390.68	1.892	0.289
17	1450.47	75.116	6.044	1460.11	1415.75	4.291	0.55
18	1494.83	83.743	3.666	1504.48	1485.19	1.301	0.174
19	1543.05	80.316	6.864	1554.63	1527.62	1.948	0.382
20	1595.13	67.513	20.197	1643.35	1556.55	9.19	4.305
21	1710.86	68.373	6.56	1718.58	1666.5	4.857	0.359
22	2139.06	96.585	1.181	2167.99	2121.7	0.567	0.125
23	2837.29	92.417	1.98	2858.51	2783.28	1.946	0.215
24	2931.8	90.954	1.739	2958.8	2914.44	1.661	0.183

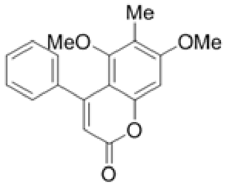

3b

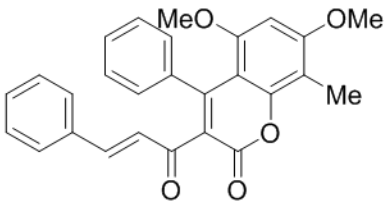

4a (^{1}H-NMR)
300 MHz, $CDCl_3$

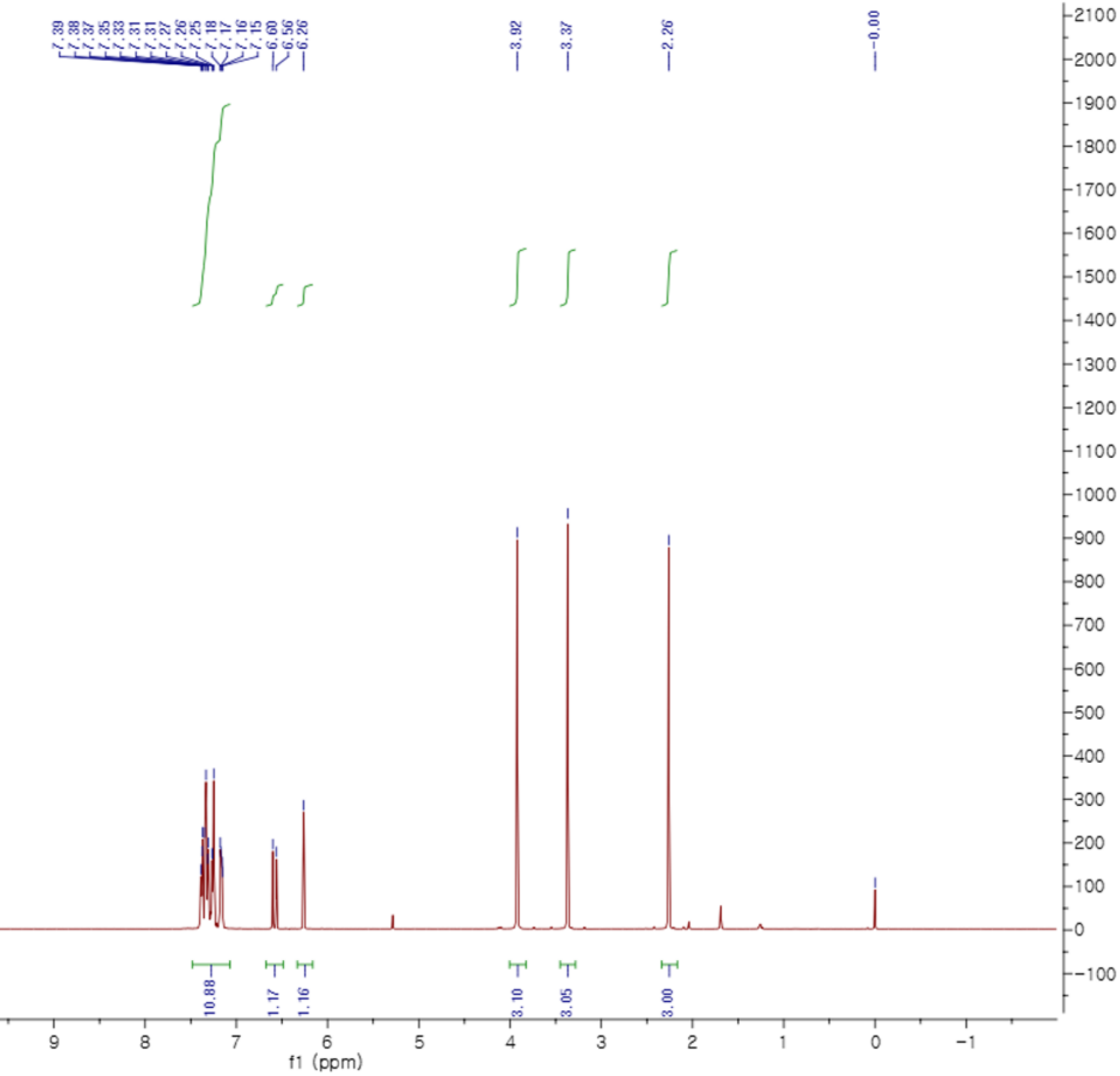

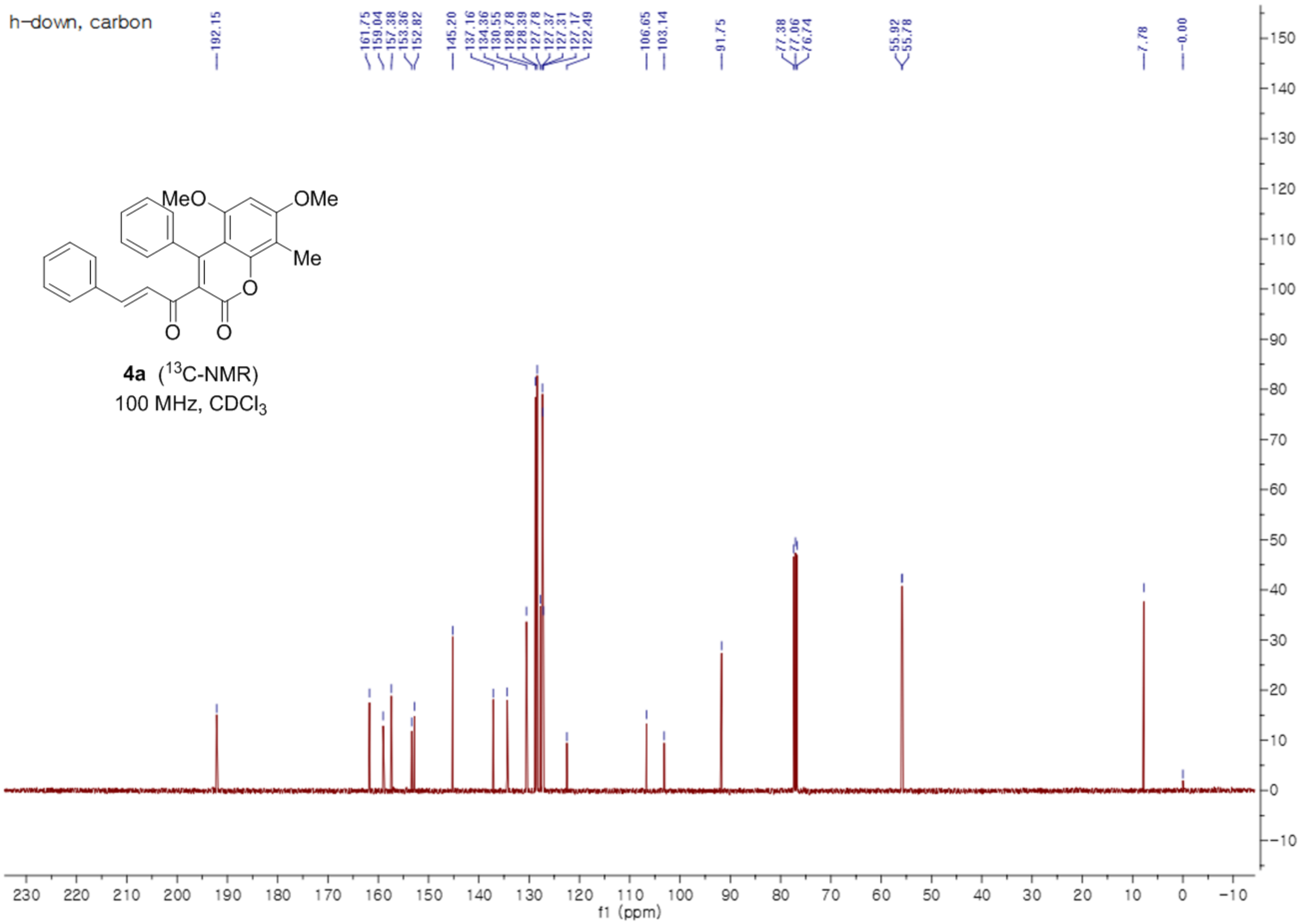

4a (^{13}C-NMR)
100 MHz, $CDCl_3$

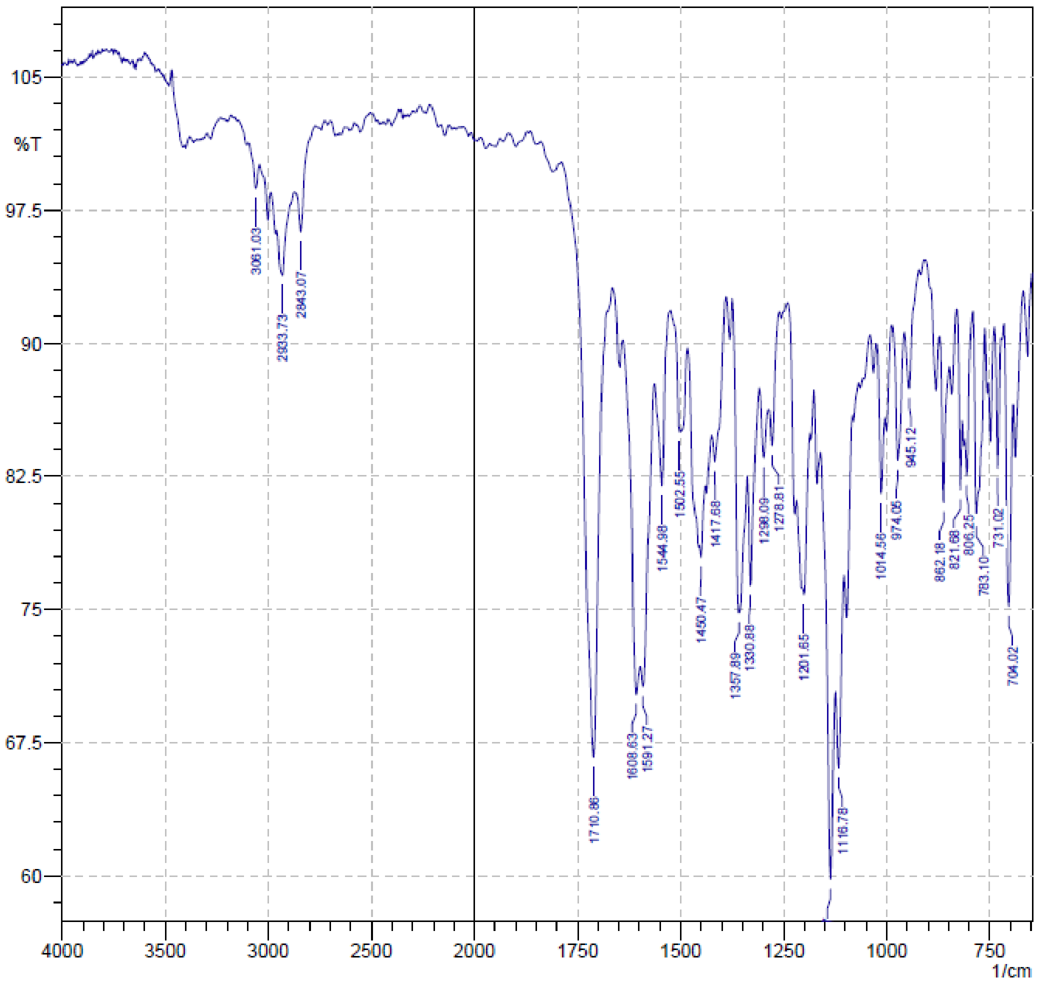

No.	Peak	Intensity	Corr. Inte	Base (H)	Base (L)	Area	Corr. Are
1	704.02	75.164	13.441	715.59	694.37	1.958	0.841
2	731.02	82.956	7.658	738.74	723.31	0.912	0.252
3	783.1	80.206	11.095	790.81	763.81	2.035	0.913
4	806.25	82.537	3.51	810.1	790.81	1.136	0.077
5	821.68	81.743	5.685	831.32	817.82	0.813	0.112
6	862.18	81.047	8.625	871.82	848.68	1.505	0.392
7	945.12	87.446	4.363	956.69	921.97	1.441	0.244
8	974.05	83.398	7.462	987.55	956.69	1.803	0.515
9	1014.56	81.512	6.182	1026.13	1004.91	1.451	0.258
10	1116.78	66.079	6.769	1124.5	1105.21	2.99	0.435
11	1136.07	59.819	14.251	1159.22	1126.43	5.065	1.183
12	1201.65	75.835	7.326	1219.01	1186.22	3.328	0.679
13	1278.81	84.227	3.62	1286.52	1259.52	1.517	0.148
14	1298.09	83.544	3.334	1309.67	1286.52	1.594	0.179
15	1330.88	76.355	6.767	1336.67	1309.67	2.33	0.406
16	1357.89	74.816	13.09	1373.32	1338.6	3.38	1.273
17	1417.68	83.328	2.776	1423.47	1390.68	2.026	0.337
18	1450.47	77.958	1.622	1454.33	1440.83	1.34	0.051
19	1502.55	85.009	5.583	1523.76	1485.19	2.14	0.494
20	1544.98	81.977	7.672	1562.34	1525.69	2.237	0.538
21	1591.27	70.662	3.938	1597.06	1564.27	3.54	0.346
22	1608.63	70.22	5.948	1639.49	1598.99	4.195	0.401
23	1710.86	66.693	29.012	1788.01	1666.5	7.681	5.821
24	2843.07	96.275	3.353	2872.01	2775.57	0.187	0.337
25	2933.73	93.84	3.089	2958.8	2893.22	1.271	0.433
26	3061.03	98.733	1.759	3097.68	3041.74	0.007	0.173

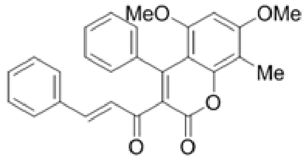

4a

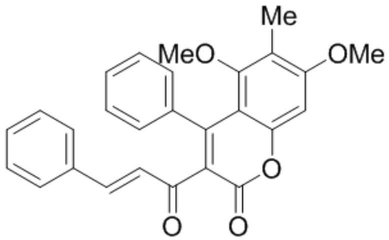

4b (^{1}H-NMR)
300 MHz, $CDCl_3$

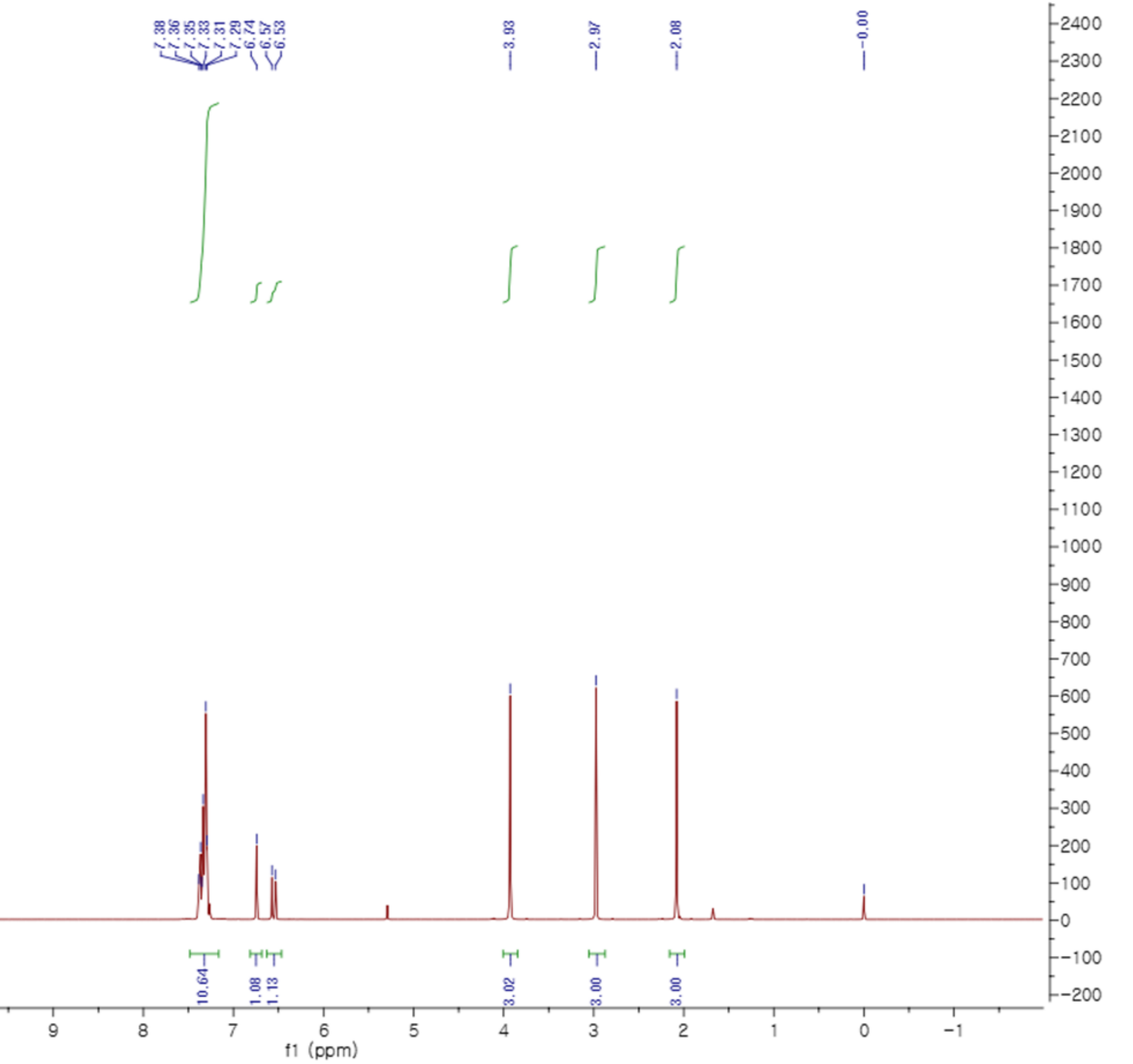

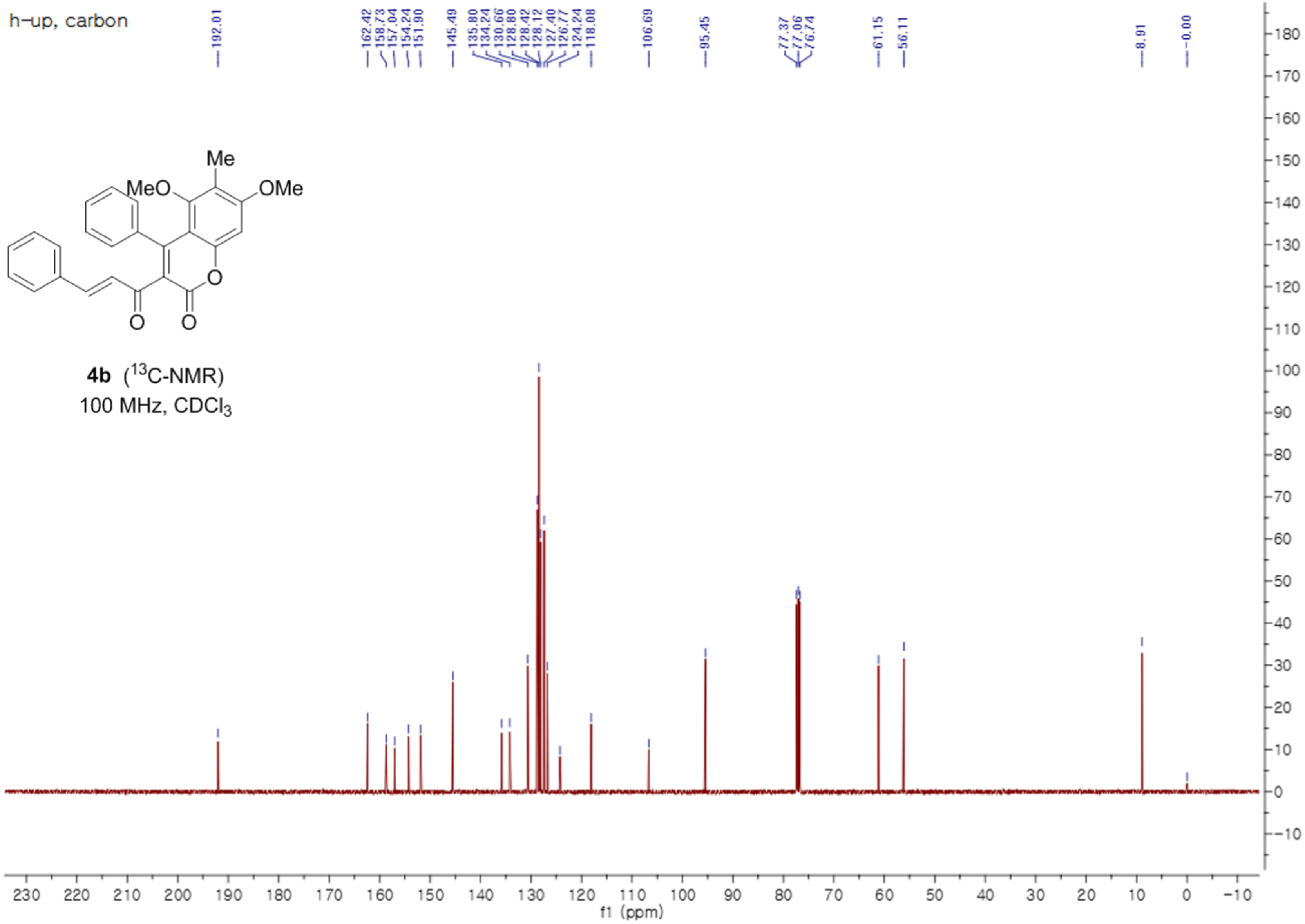

4b (^{13}C-NMR)
100 MHz, $CDCl_3$

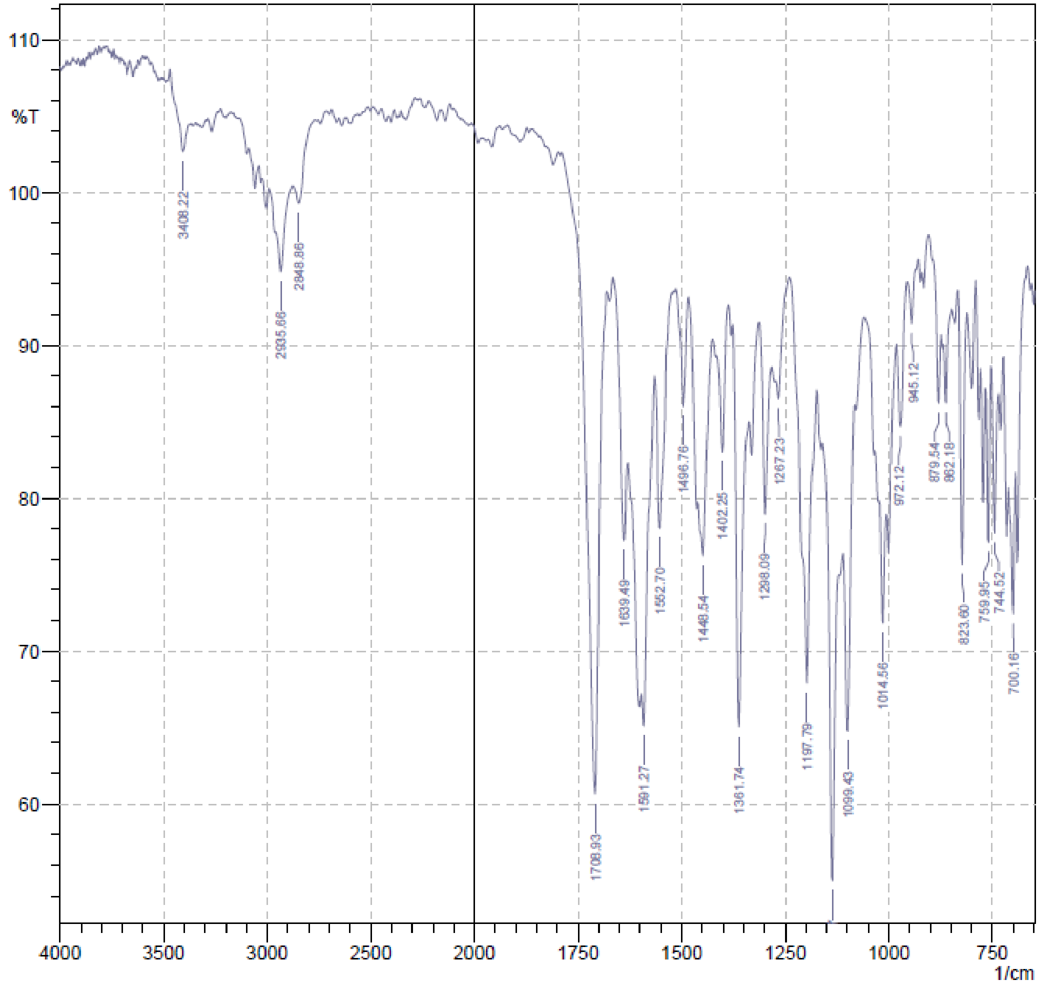

No.	Peak	Intensity	Corr. Inte	Base (H)	Base (L)	Area	Corr. Are
1	700.16	72.436	8.929	711.73	694.37	1.934	0.364
2	744.52	77.727	10.022	754.17	734.88	1.501	0.406
3	759.95	77.129	10.893	767.67	754.17	1.112	0.357
4	823.6	75.682	17.248	833.25	812.03	1.481	0.798
5	862.18	86.235	4.507	867.97	848.68	0.895	0.123
6	879.54	86.216	5.704	904.61	873.75	1.018	0.175
7	945.12	91.418	3.187	956.69	935.48	0.639	0.124
8	972.12	84.689	6.944	981.77	956.69	1.315	0.417
9	1014.56	71.851	7.501	1024.2	1006.84	2.068	0.329
10	1099.43	64.764	15.743	1111	1083.99	3.621	1.207
11	1136.07	54.98	23.097	1159.22	1122.57	5.72	1.942
12	1197.79	67.904	21.823	1238.3	1174.65	5.746	2.999
13	1267.23	86.51	2.356	1273.02	1240.23	1.396	0.053
14	1298.09	78.981	10.899	1313.52	1284.59	2.025	0.683
15	1361.74	65.052	23.502	1375.25	1338.6	4.18	2.077
16	1402.25	83.013	7.878	1415.75	1386.82	1.639	0.452
17	1448.54	76.257	7.083	1460.11	1427.32	2.839	0.536
18	1496.76	86.009	7.403	1514.12	1483.26	1.358	0.449
19	1552.7	78.053	11.358	1564.27	1525.69	2.67	1.022
20	1591.27	65.122	5.963	1597.06	1566.2	3.685	0.299
21	1639.49	77.25	7.787	1664.57	1631.78	2.329	0.49
22	1708.93	60.655	35.152	1788.01	1681.93	7.061	6.057
23	2848.86	99.302	2.252	2875.86	2771.71	-0.88	0.27
24	2935.66	94.809	3.44	2958.8	2875.86	0.776	0.38
25	3408.22	102.675	2.372	3444.87	3379.29	-1.135	0.289

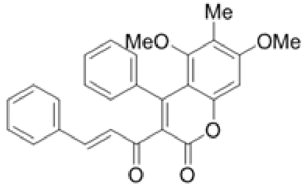

4b

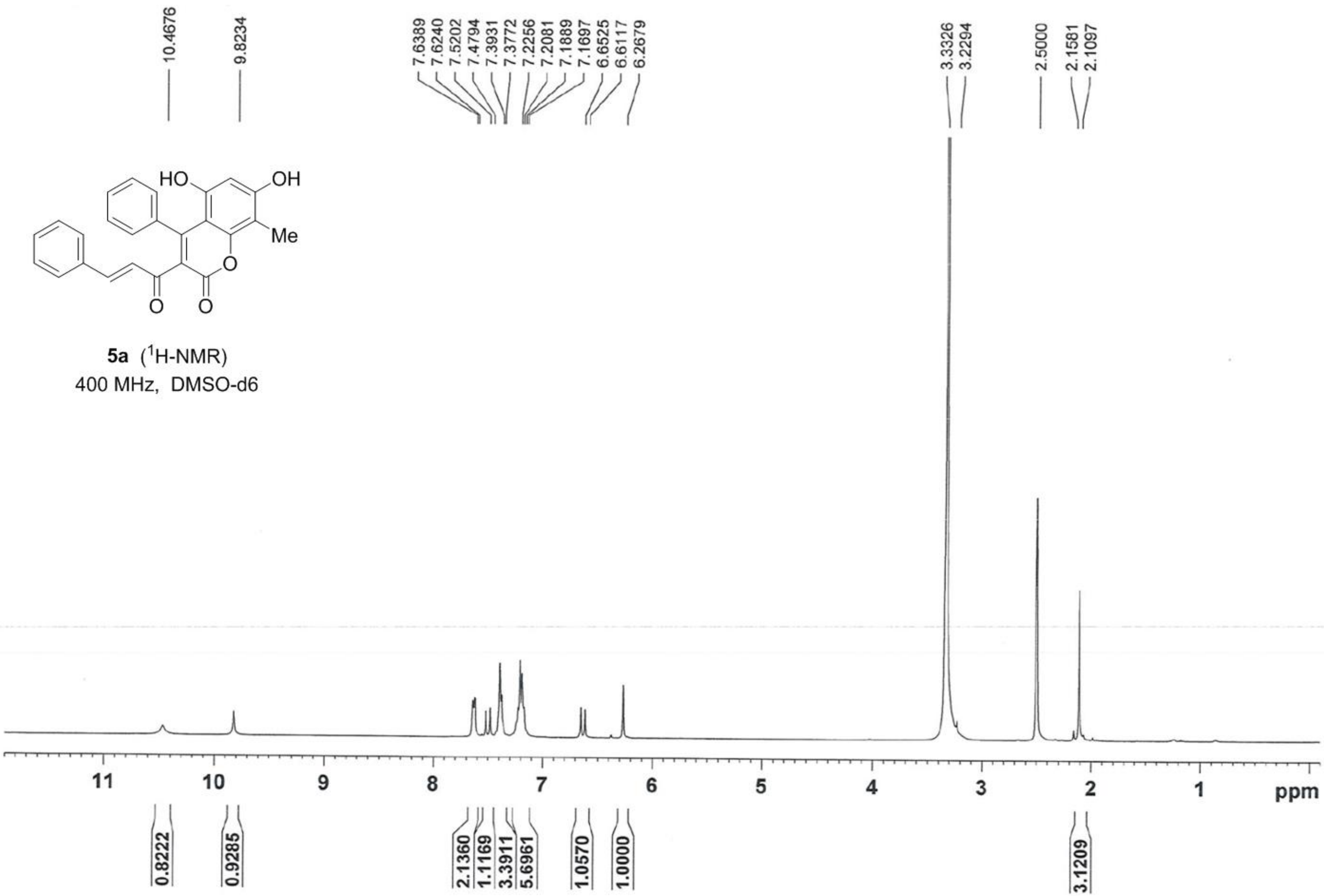
10.4676
9.8234
7.6389
7.6240
7.5202
7.4794
7.3931
7.3772
7.2256
7.2081
7.1889
7.1697
6.6525
6.6117
6.2679
3.3326
3.2294
2.5000
2.1581
2.1097
HO
OH
Me
O
O
O
5a (1H-NMR)
400 MHz, DMSO-d6
11
10
9
8
7
6
5
4
3
2
1
ppm
0.8222
0.9285
2.1360
1.1169
3.3911
5.6961
1.0570
1.0000
3.1209

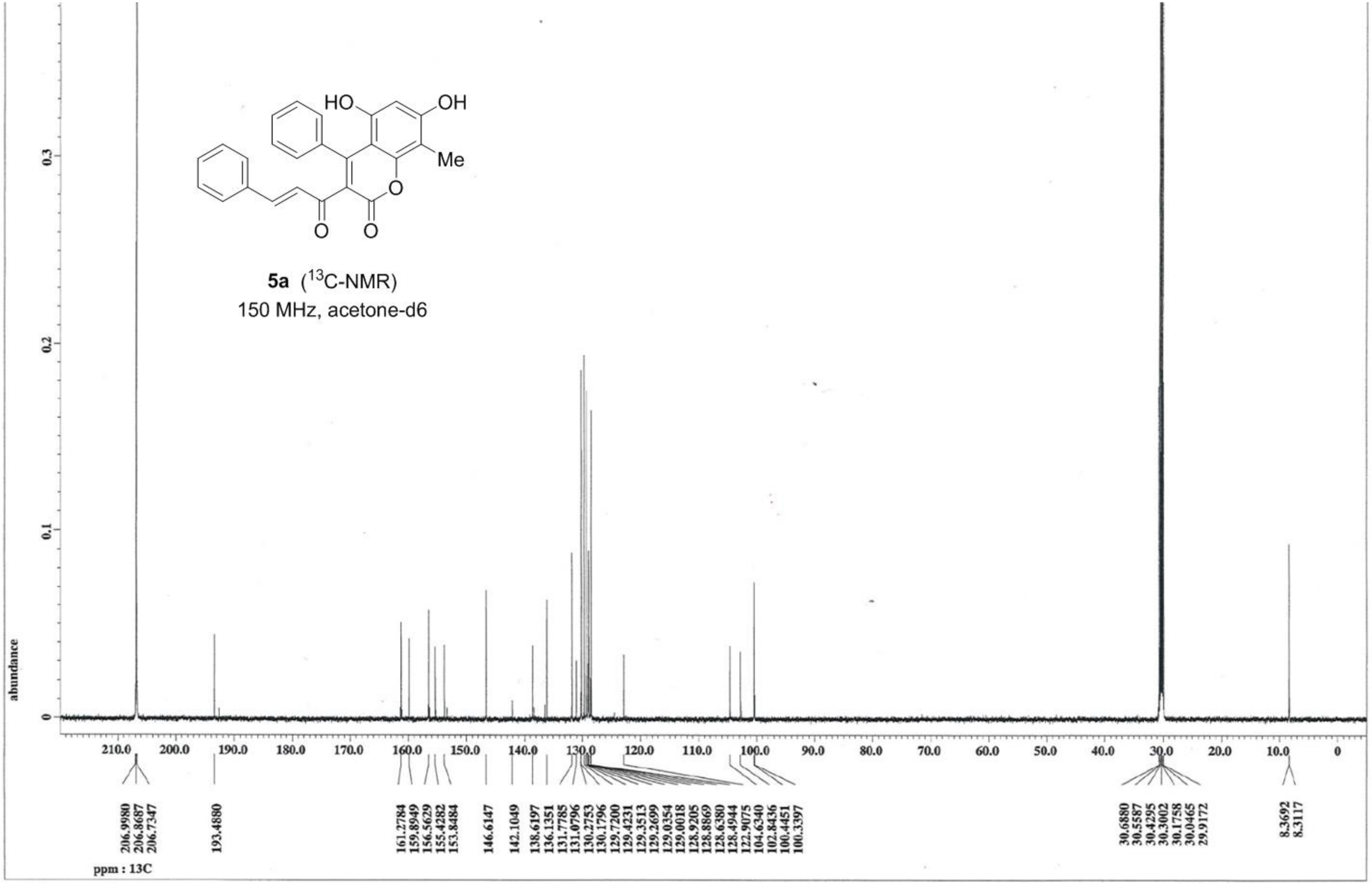
HO
OH
Me
O
O
O
5a (13C-NMR)
150 MHz, acetone-d6
abundance
0
0.1
0.2
0.3
210.0 200.0 190.0 180.0 170.0 160.0 150.0 140.0 130.0 120.0 110.0 100.0 90.0 80.0 70.0 60.0 50.0 40.0 30.0 20.0 10.0 0
206.9980
206.8687
206.7347
193.4880
161.2784
159.8949
156.5629
155.4282
153.8484
146.6147
142.1049
138.6197
136.1351
131.7785
131.0796
130.2753
130.1796
129.7200
129.4231
129.3513
129.2699
129.0354
129.0018
128.9205
128.8869
128.6380
128.4944
122.9075
104.6340
102.8436
100.4451
100.3397
30.6880
30.5587
30.4295
30.3002
30.1758
30.0465
29.9172
8.3692
8.3117
ppm : 13C

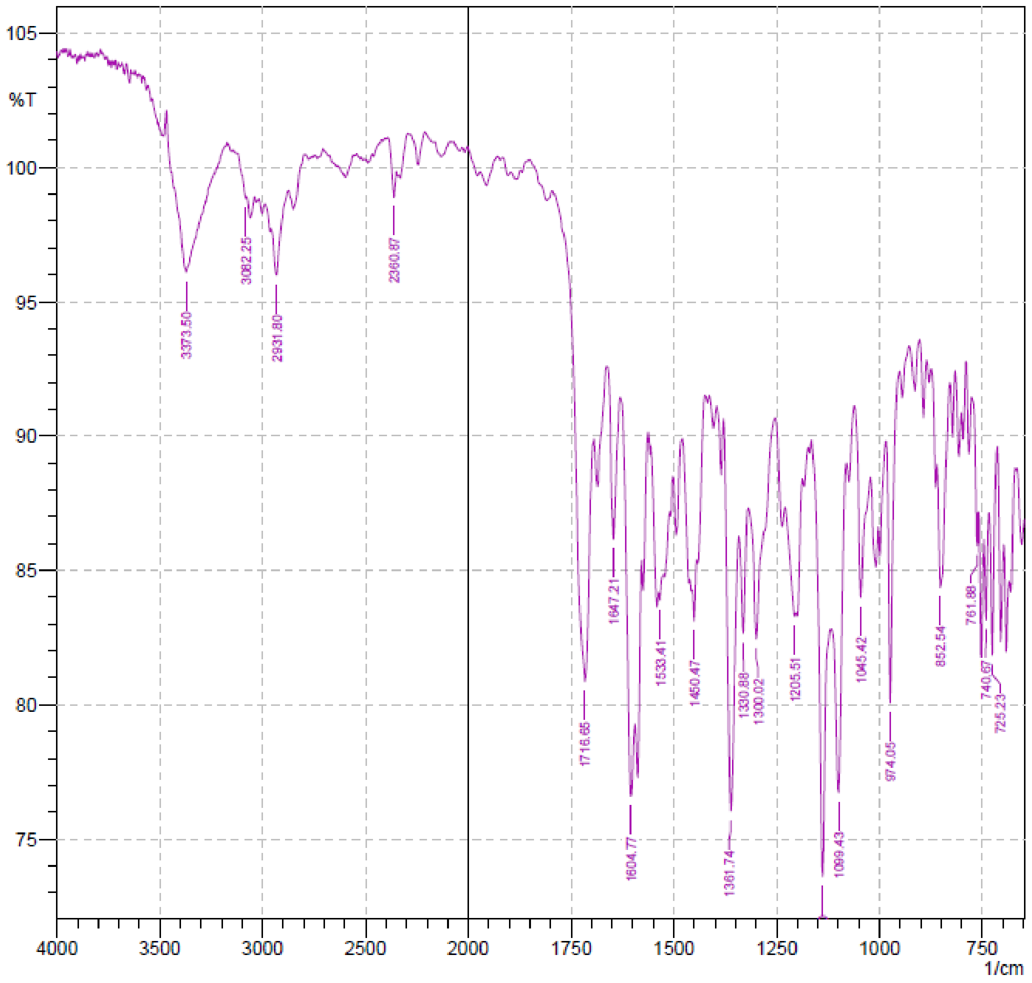

No.	Peak	Intensity	Corr. Inte	Base (H)	Base (L)	Area	Corr. Are
1	725.23	81.849	6.278	732.95	713.66	1.273	0.237
2	740.67	83.16	3.417	746.45	732.95	0.934	0.094
3	761.88	85.917	1.735	777.31	759.95	0.849	-0.006
4	852.54	84.371	5.342	860.25	829.39	1.788	0.444
5	974.05	80.068	10.503	983.7	950.91	1.893	0.565
6	1045.42	83.994	6.069	1060.85	1022.27	2.267	0.45
7	1099.43	76.724	9.141	1116.78	1082.07	3.033	0.756
8	1138	73.595	12.151	1165	1118.71	4.018	1.012
9	1205.51	83.271	0.427	1230.58	1203.58	1.873	0.018
10	1300.02	82.448	5.861	1319.31	1253.73	4.054	0.746
11	1330.88	82.655	4.138	1342.46	1319.31	1.627	0.203
12	1361.74	76.042	12.427	1377.17	1342.46	3.053	1.146
13	1450.47	83.123	1.604	1454.33	1444.68	0.729	0.04
14	1533.41	83.882	0.503	1537.27	1523.76	0.99	0.005
15	1604.77	76.581	6.075	1629.85	1595.13	2.832	0.4
16	1647.21	86.176	5.869	1660.71	1631.78	1.418	0.371
17	1716.65	80.85	11.043	1788.01	1695.43	4.018	1.67
18	2360.87	98.887	1.311	2391.73	2349.3	0.004	0.093
19	2931.8	95.997	2.208	2960.73	2873.94	0.905	0.31
20	3082.25	98.856	0.258	3124.68	3076.46	0.066	0.008
21	3373.5	96.095	0.652	3410.15	3363.86	0.625	0.08

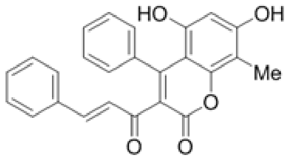

5a

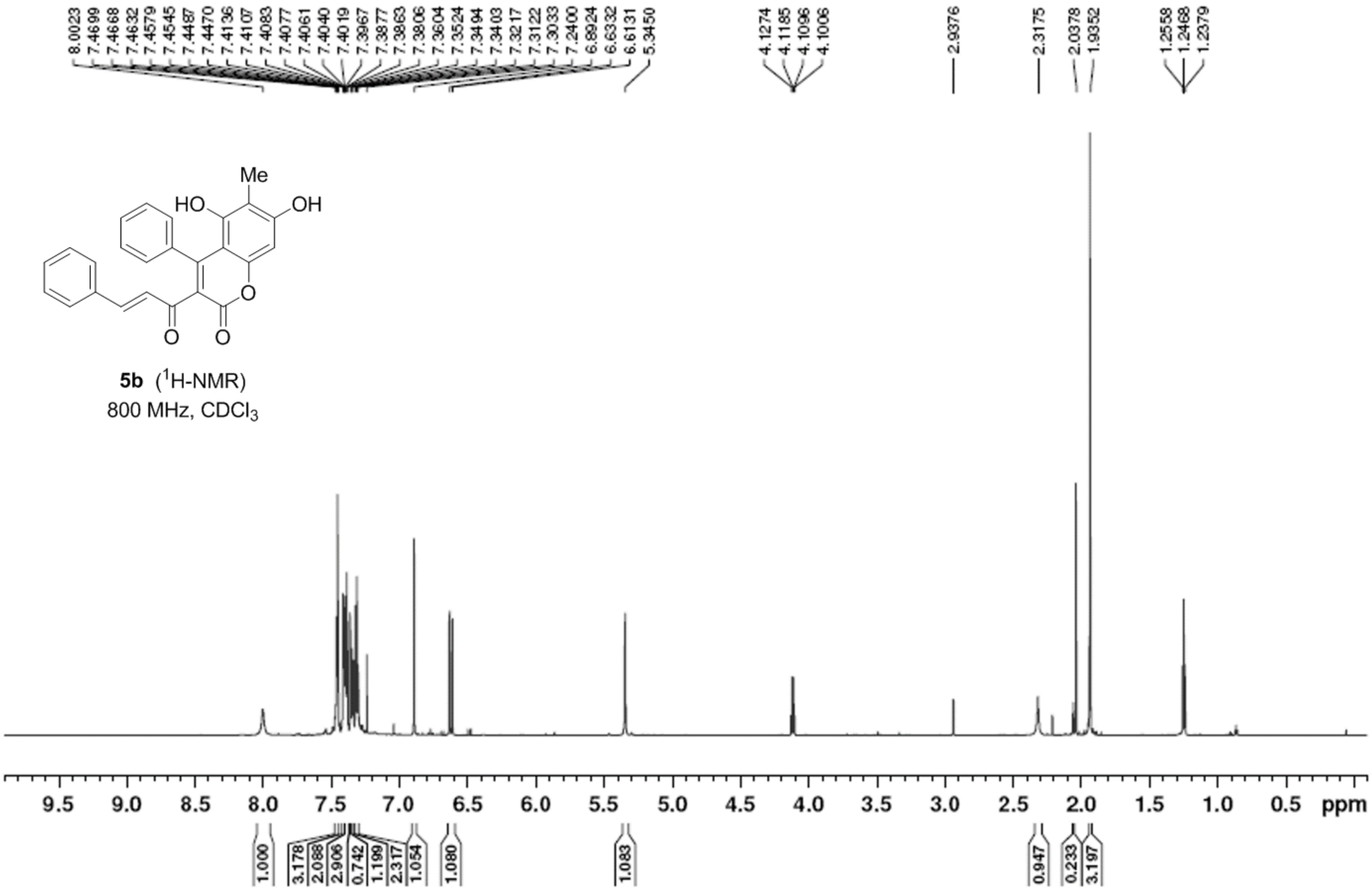

5b (^{1}H-NMR)
800 MHz, $CDCl_3$

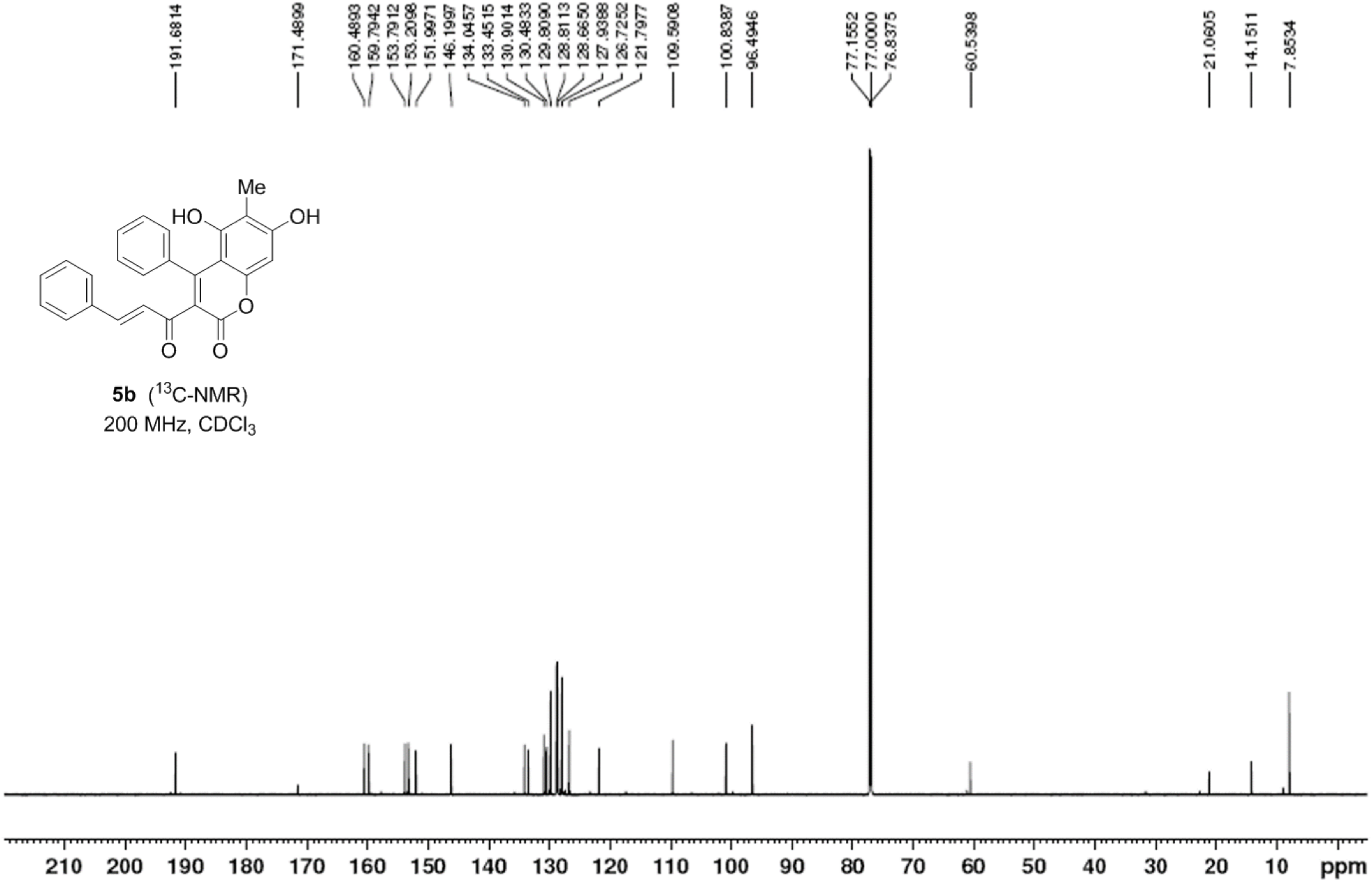

5b (^{13}C-NMR)
200 MHz, $CDCl_3$

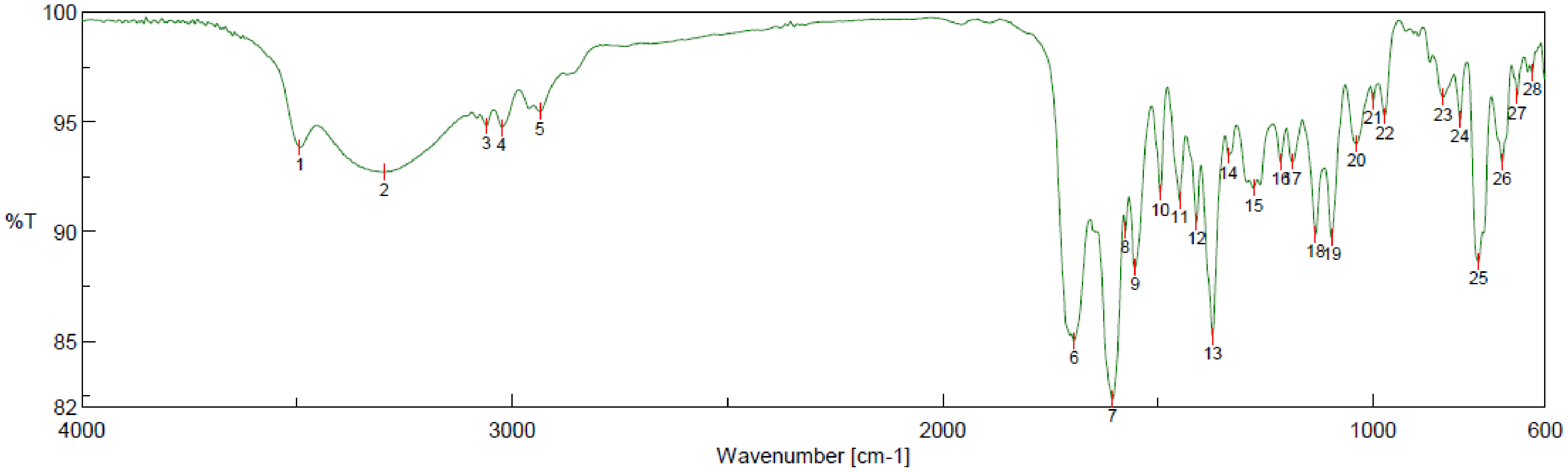

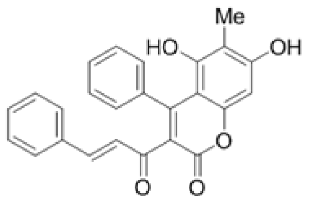

5b

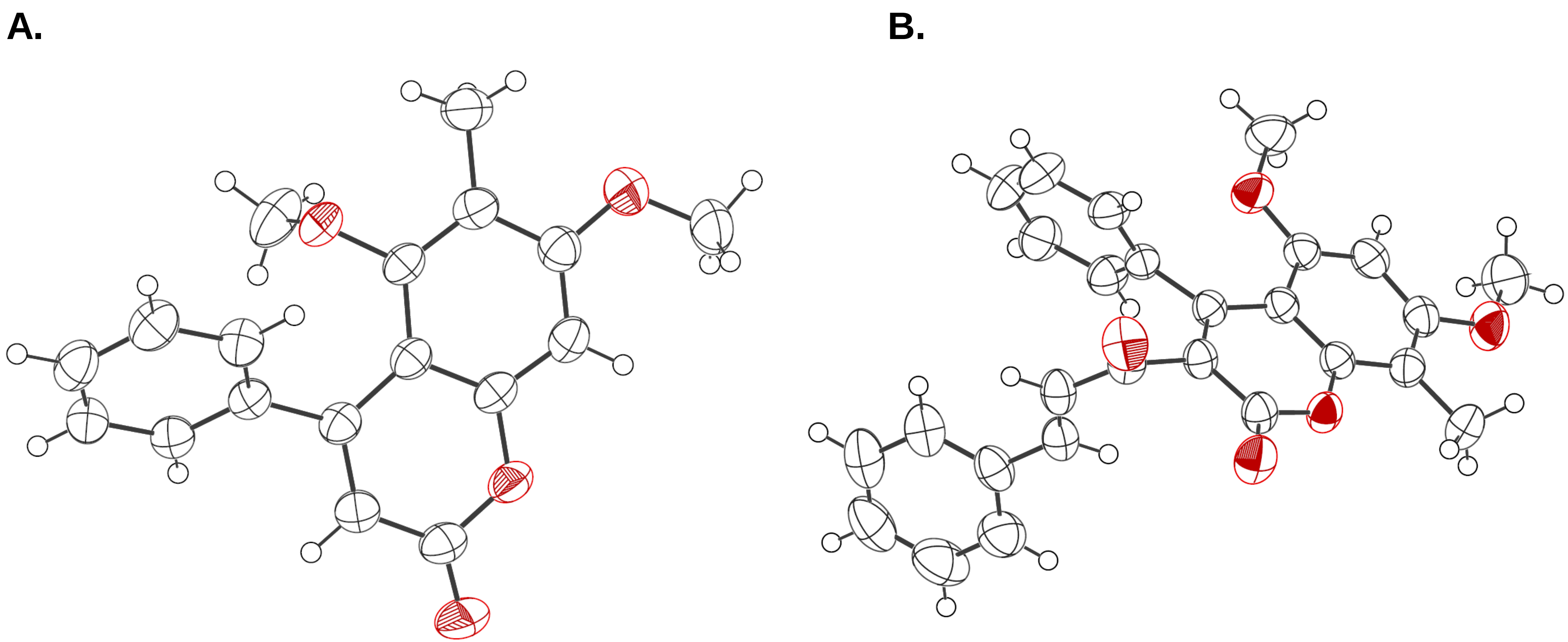

Supplementary Fig. 1. Kim et al

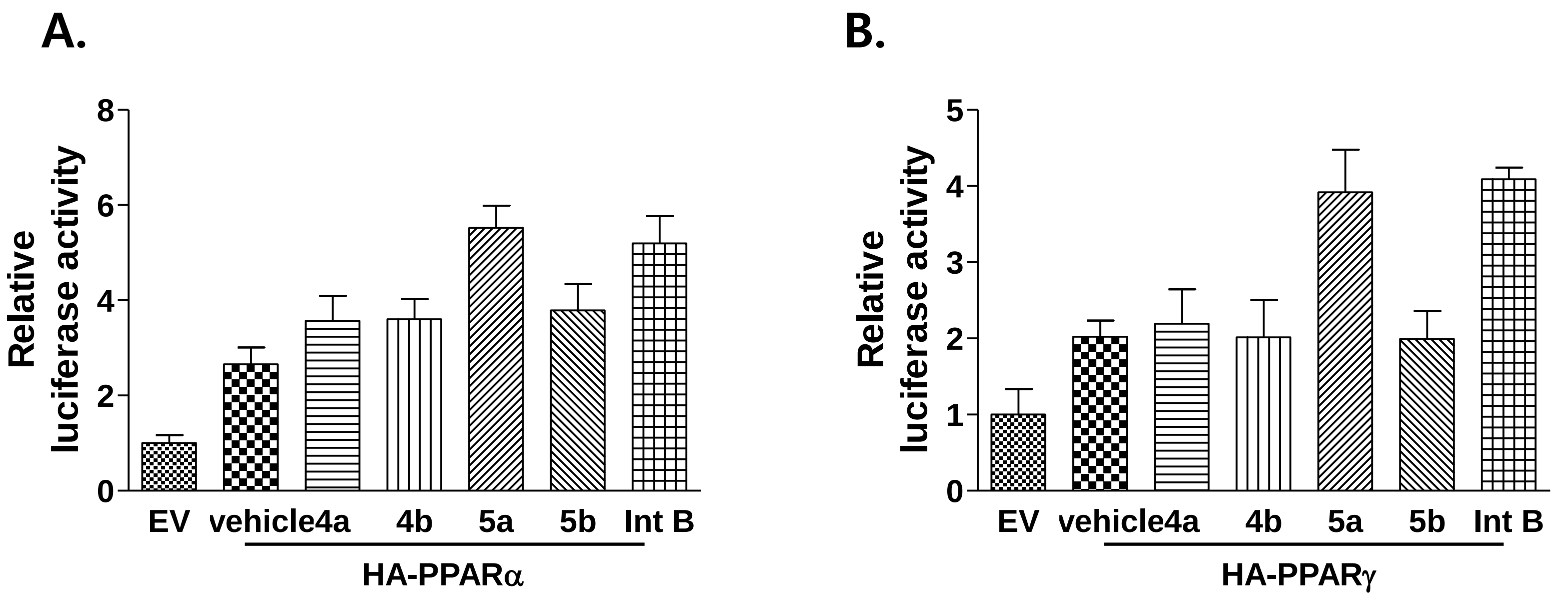

Supplementary Fig. 2. Kim et al

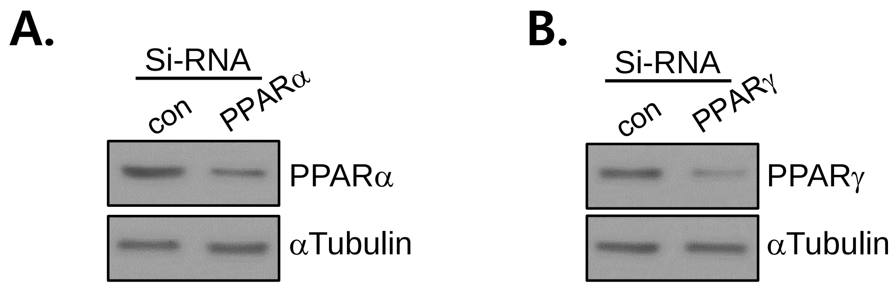

Supplementary Fig. 3. Kim et al

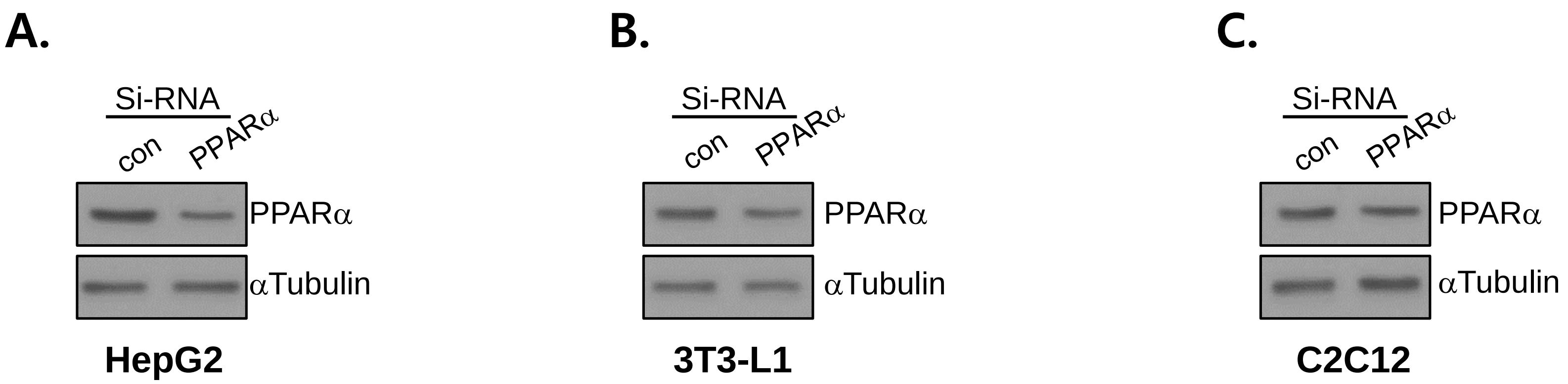

Supplementary Fig. 4. Kim et al

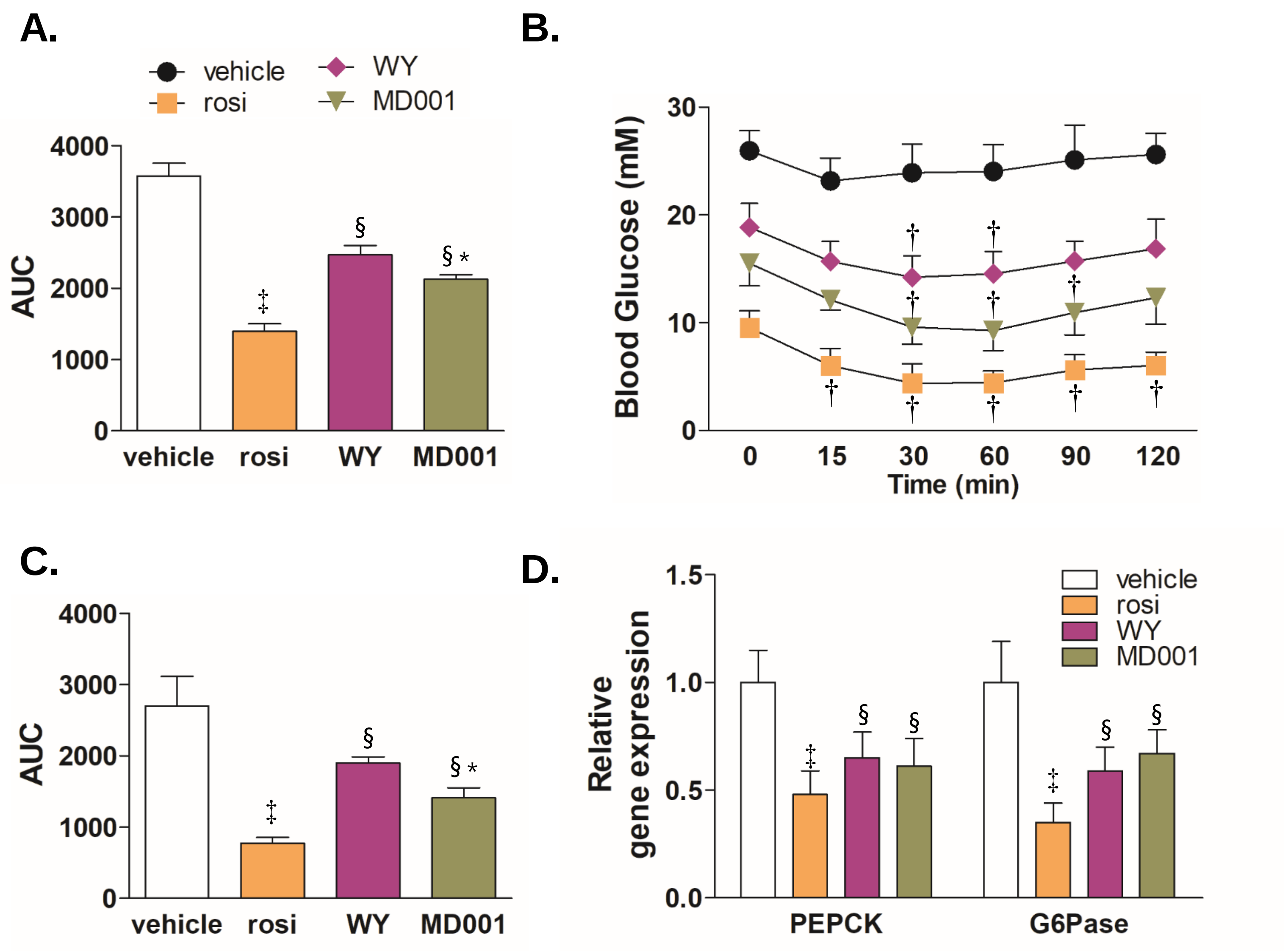

Supplementary Fig. 5. Kim et al

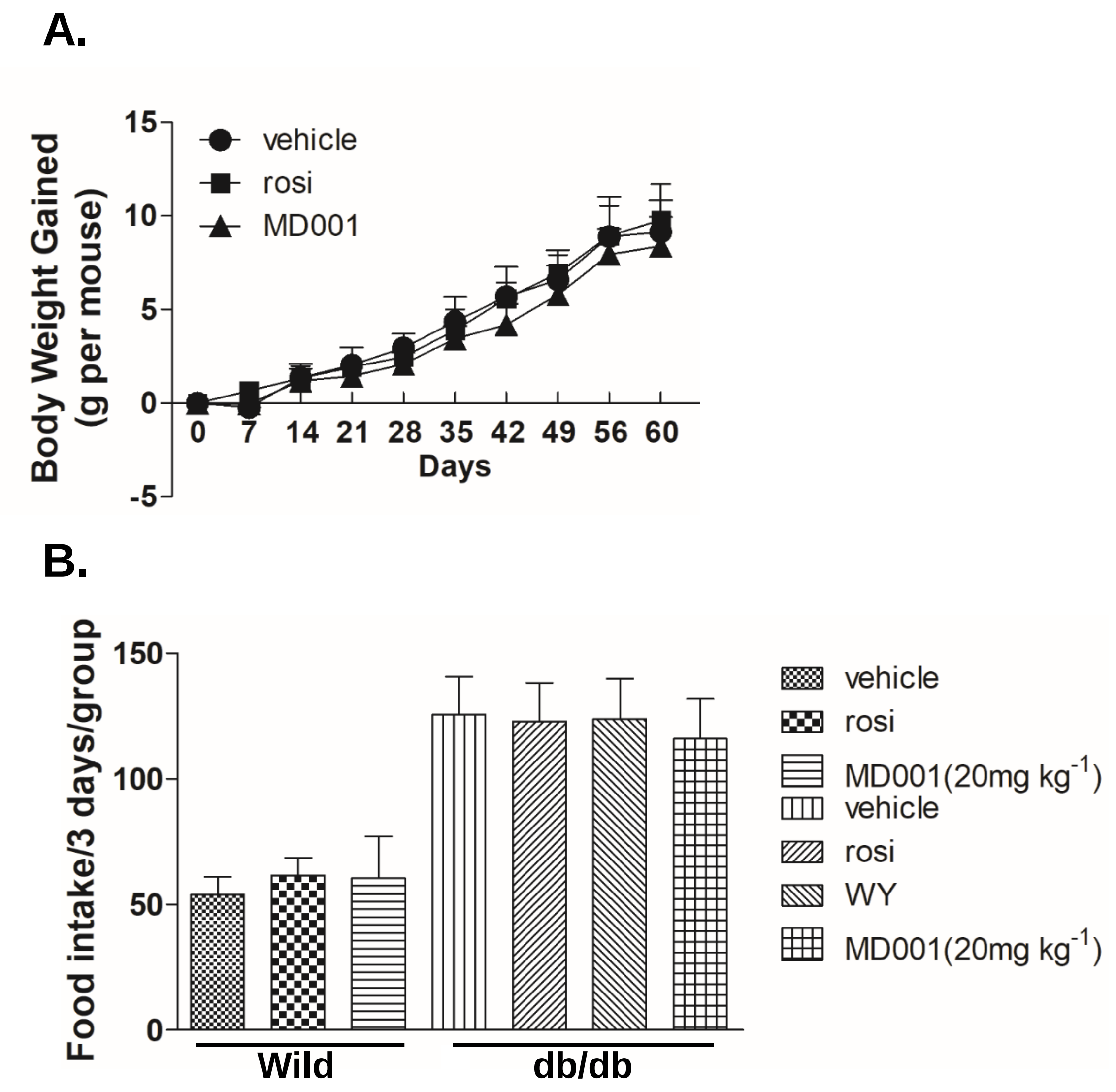

Supplementary Fig. 6. Kim et al.

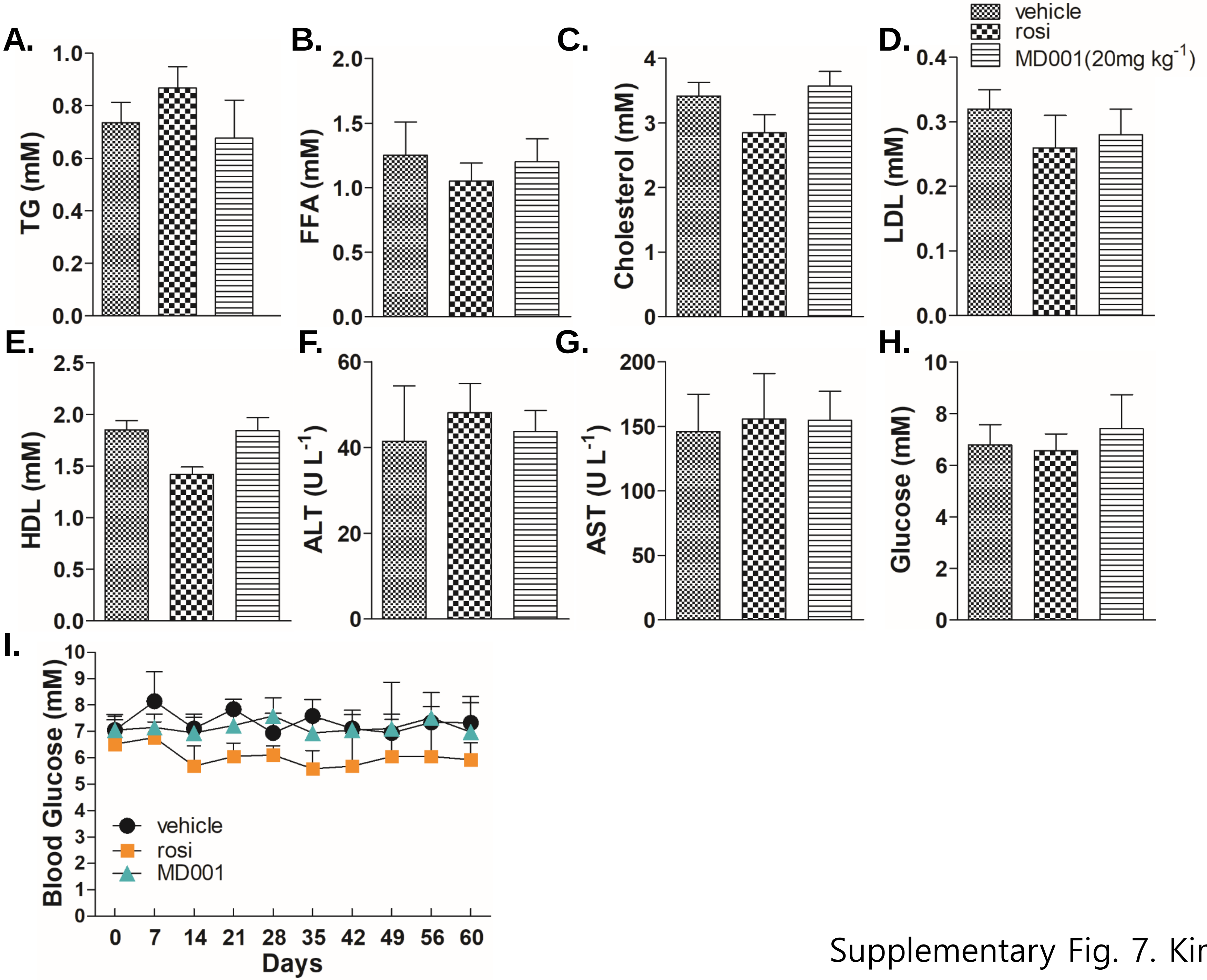

Supplementary Fig. 7. Kim et al

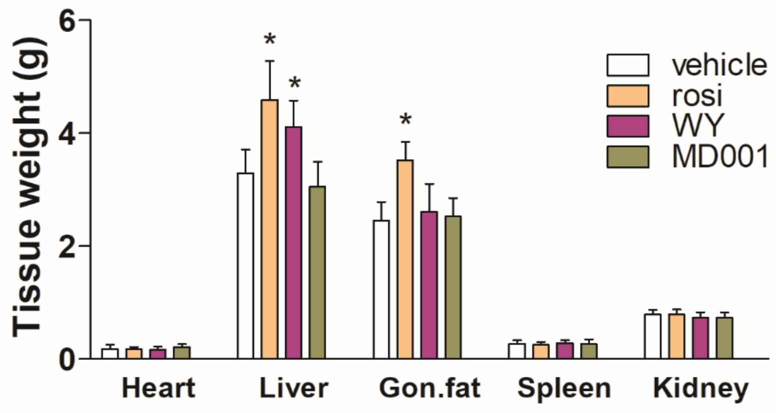

Supplementary Fig. 8. Kim et al.

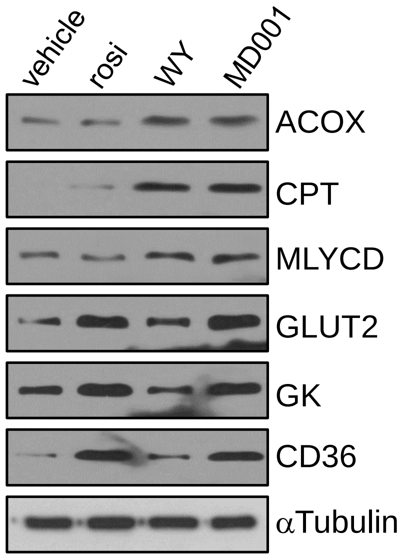

Supplementary Fig. 9. Kim et al.

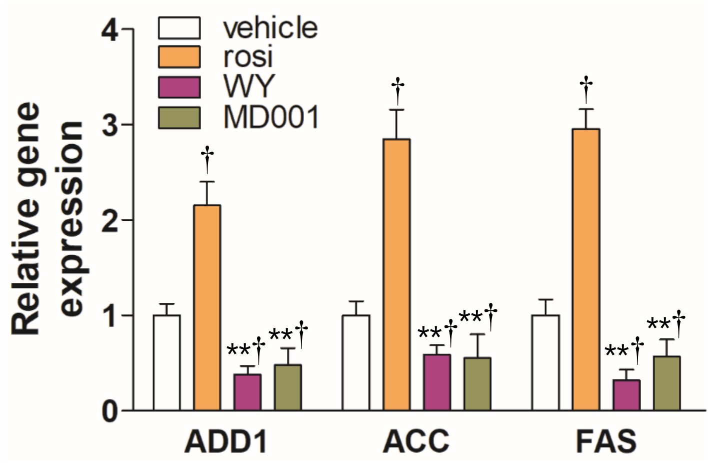

Supplementary Fig. 10. Kim et al.

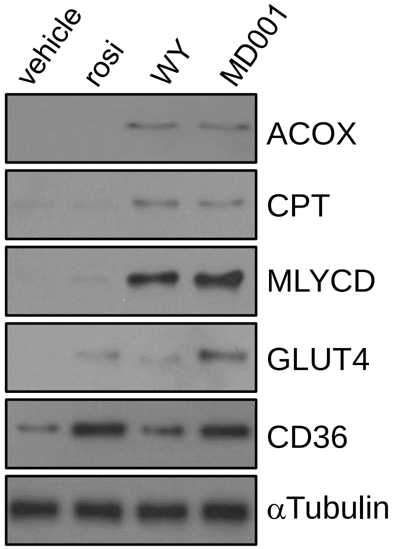

Supplementary Fig. 11. Kim et al.

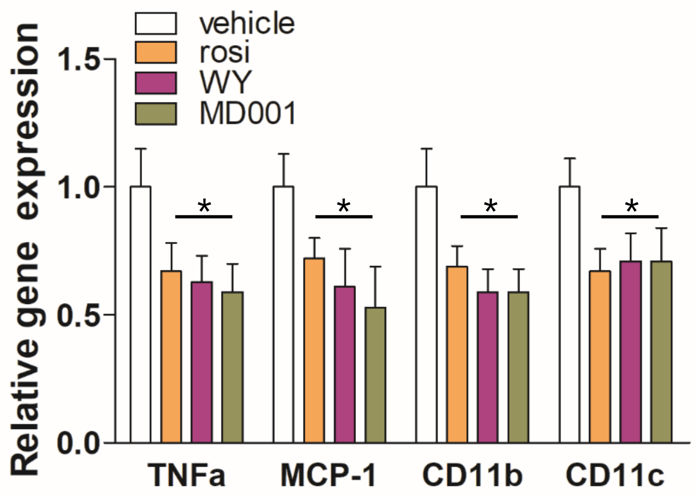

Supplementary Fig. 12. Kim et al.

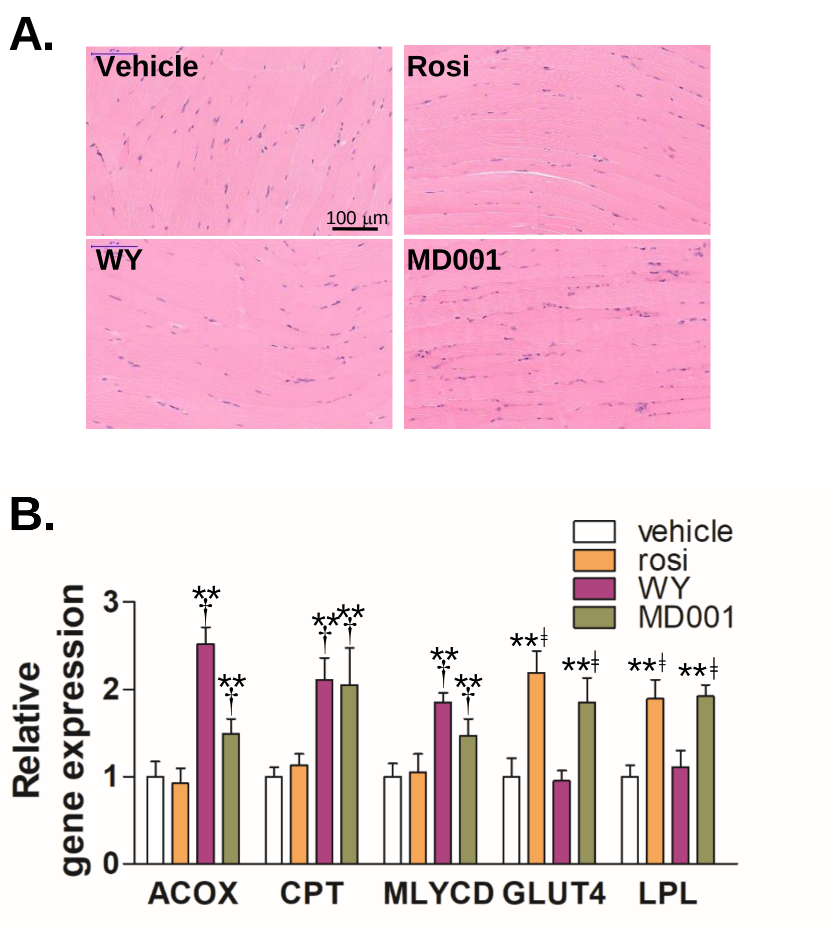

Supplementary Fig. 13. Kim et al.

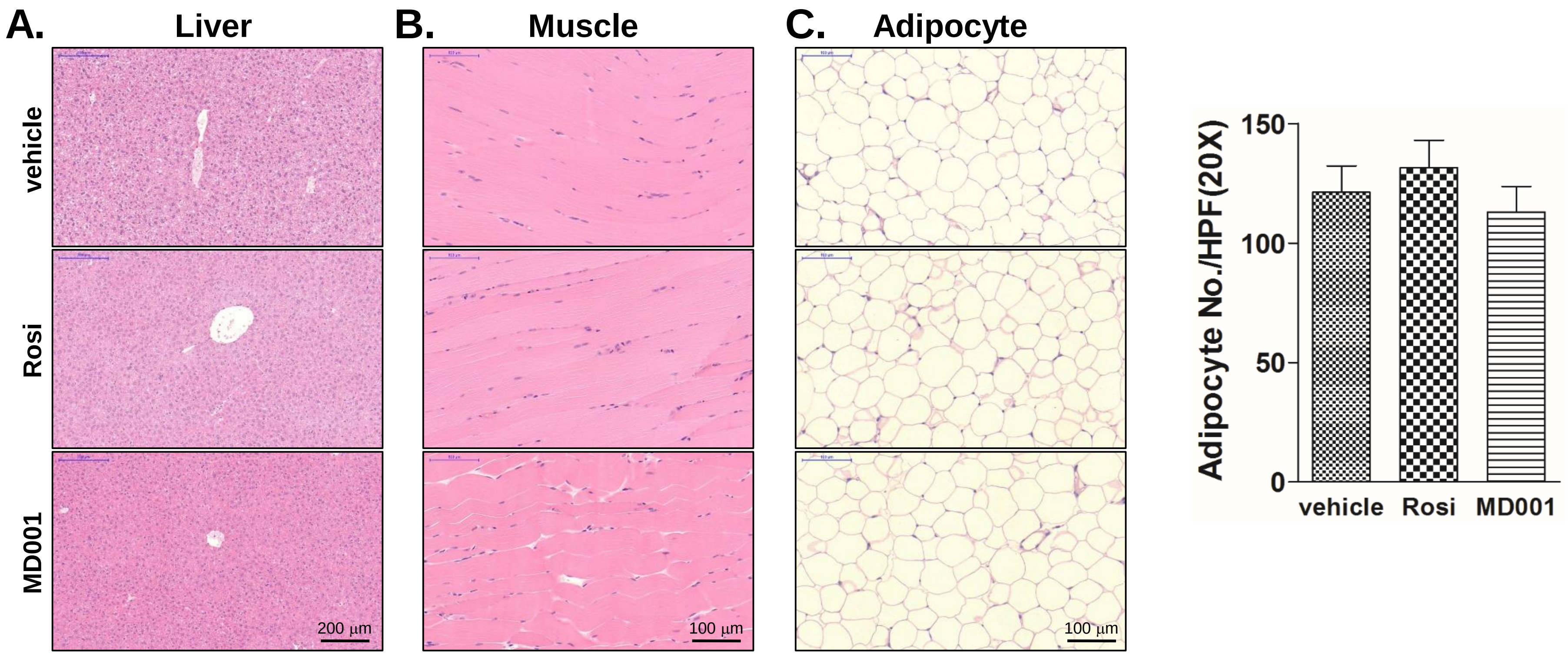

Supplementary Fig. 14. Kim et al.

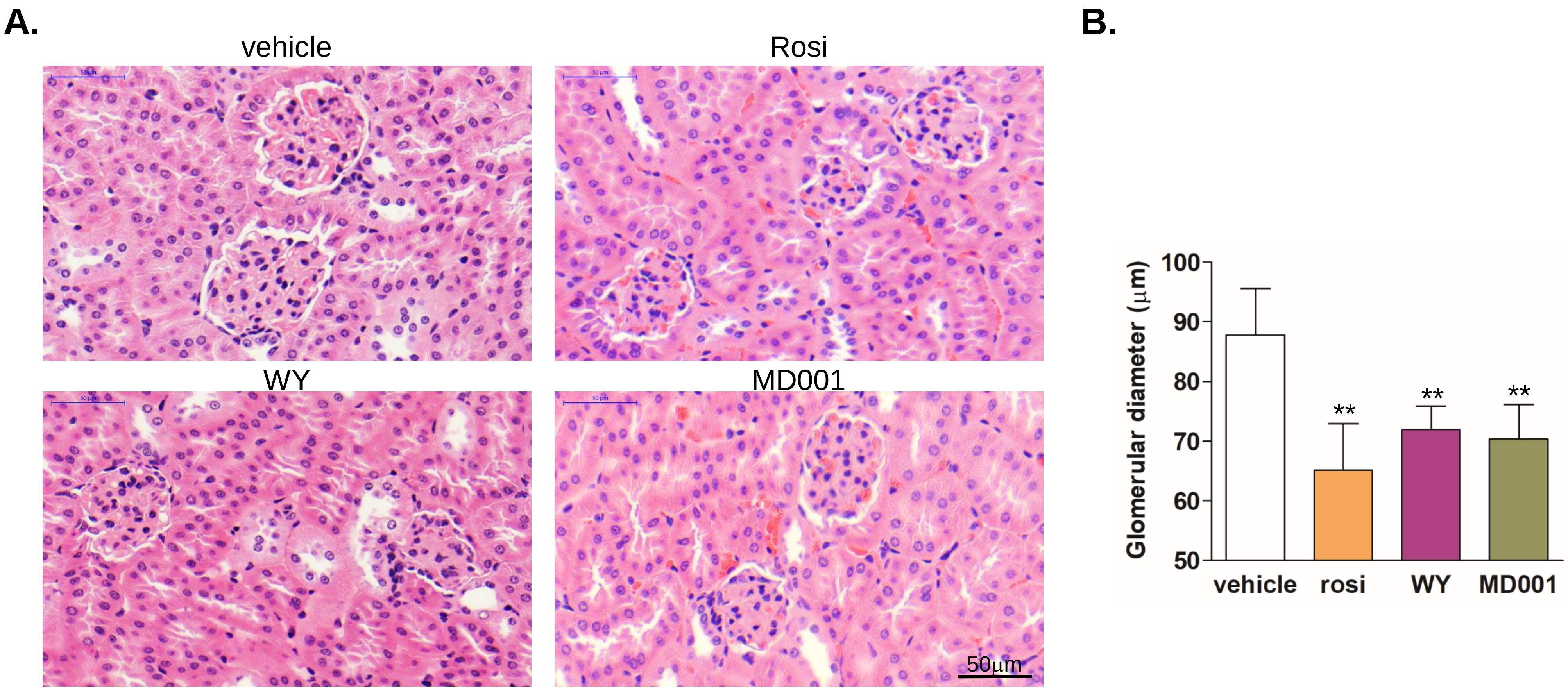

Supplementary Fig. 15. Kim et al.

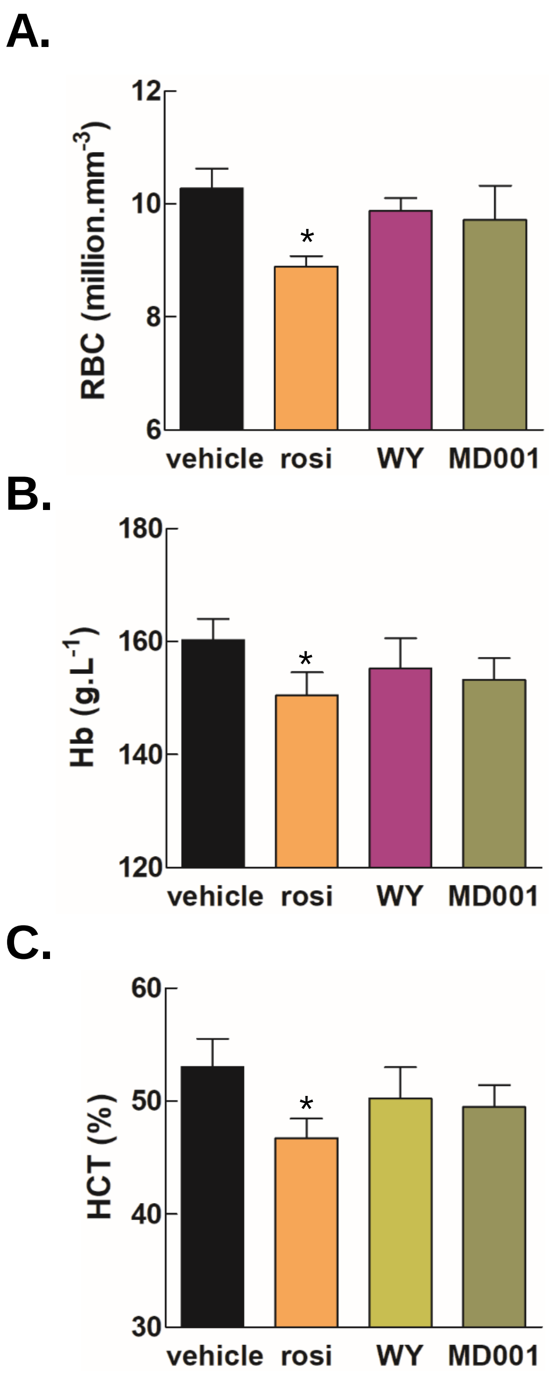

Supplementary Fig. 16. Kim et al.

Whole blot image

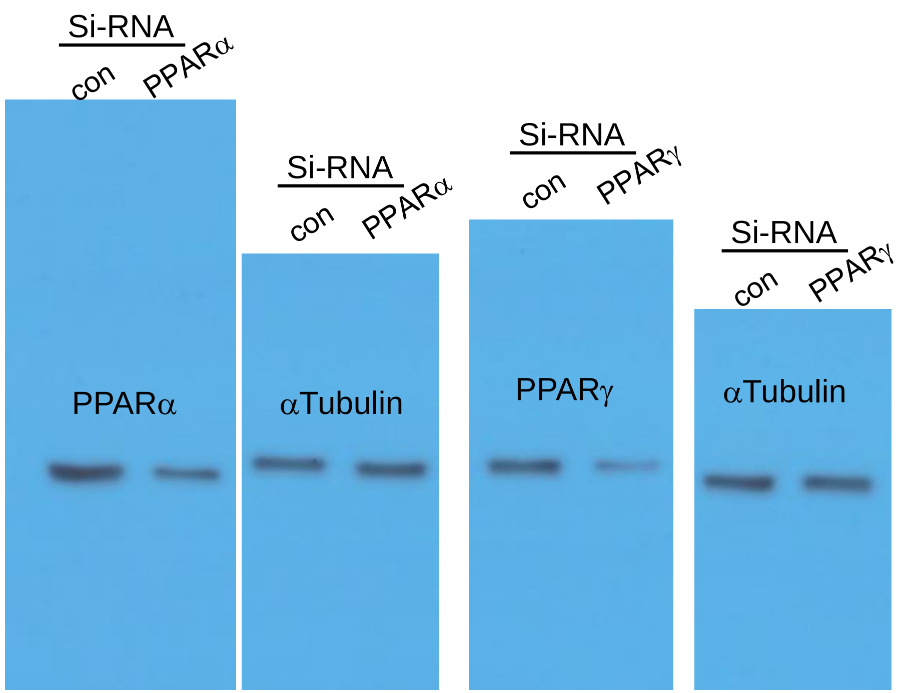

Supplementary Fig. 3.

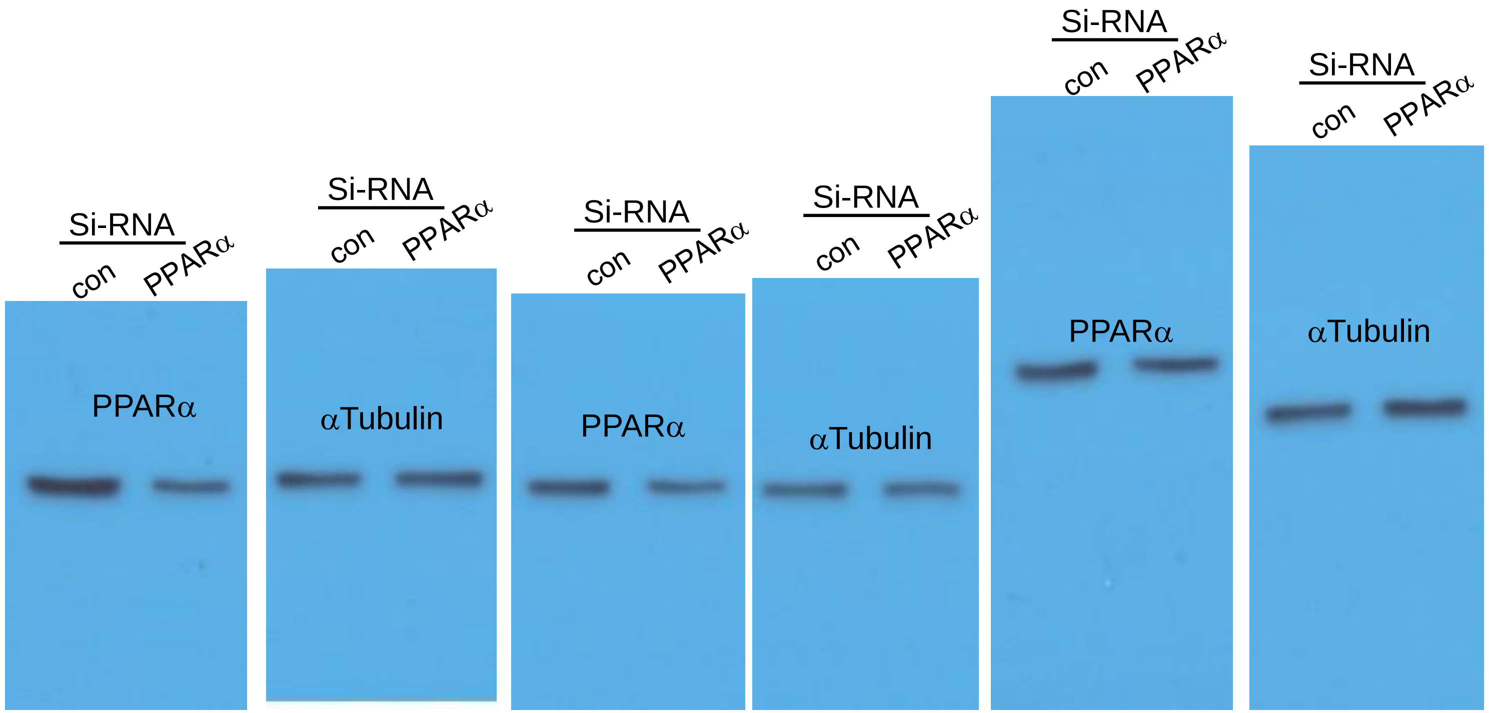

Supplementary Fig. 4.

Whole blot image

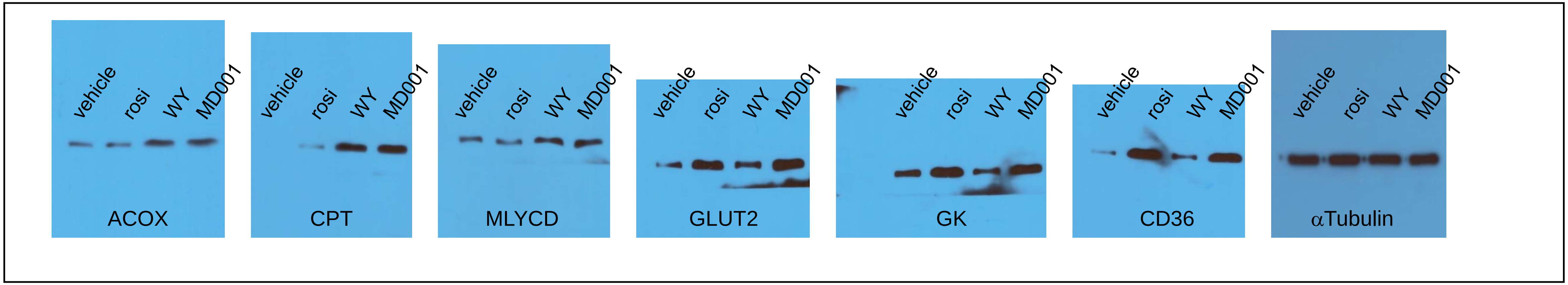

Supplementary Fig. 9.

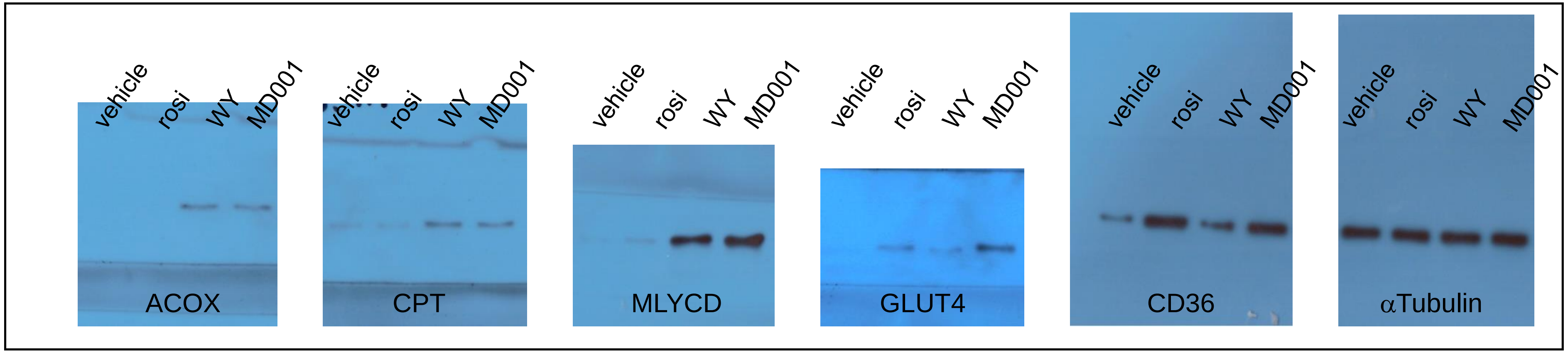

Supplementary Fig. 11.
